# Induction of cellular senescence by androgen receptor agonist or antagonist is mediated via two novel common DYRK1A-DREAM and cyclin G2 signaling pathways in castration-resistant prostate cancer

**DOI:** 10.1016/j.jare.2025.05.019

**Published:** 2025-05-12

**Authors:** Golnaz Atri Roozbahani, Mehdi Heidari Horestani, Katrin Schindler, Julia Kallenbach, Aria Baniahmad

**Affiliations:** Institute of Human Genetics, Jena University Hospital, Jena, Germany

**Keywords:** Androgen receptor, Antiandrogens, Supraphysiological androgen levels, Cellular senescence, Castration-resistant prostate cancer

## Abstract

**Background:**

Castration-resistant prostate cancer (CRPC) is a deadly disease that in addition to being resistant to androgen deprivation often exhibits also resistant to androgen receptor (AR)-antagonists. Supraphysiological androgen levels (SAL) are used in the bipolar androgen therapy in clinical phase trials to inhibit CRPC.

**Methods:**

Co-immunoprecipitation, immunofluorescence, growth and cell senescence assays, mouse xenografted CRPC, AR target gene analyses of organs, analyses of RNA-seq, ChIP-seq and patient-derived xenografts, qRT-PCR, 3D tumor spheroids, knockdown experiments.

**Results:**

Both AR-antagonists and –agonist at SAL induce cellular senescence despite acting oppositely on AR transcriptional activity. Here, we identified two common growth inhibitory pathways induced by SAL and AR-antagonists. Using the novel AR-antagonist compound 28 (C28), that inhibits also those AR mutants mediating therapy resistance, represses growth including CRPC tumor spheroids and mouse xenografted tumors. Mechanistically, C28 reduces phosphorylation of AR at serine 81 and HSP27 required for AR transcriptional activity and enhances AR-p130 interaction. Notably, increased p130 levels were also detected by SAL treatment leading to activation of DREAM complex signaling and induction of cellular senescence indicating that both AR-agonist and –antagonist use the same pathway for growth repression. Inhibition of DYRK1A, a key kinase to activate DREAM complex, blocks C28- and SAL-induced cellular senescence. Both C28 and SAL also induces the atypical cyclin G2 (*CCNG2*), which also mediates cellular senescence. Induction of *CCNG2* is confirmed in CRPC tumor spheroids and xenograft tumors of treated mice. The second-generation AR-antagonist Darolutamide (Dar) also activates DREAM complex and *CCNG2*.

**Conclusion:**

Taken together, these findings suggest the identification of two common and independently acting pathways induced by AR-antagonists and SAL, used in bipolar androgen therapy, to mediate growth inhibition and induction of cellular senescence in CRPC.

## Highlights

• Both SAL and the AR-antagonist C28 induce cellular senescence in CRPC.
• C28 but not SAL inhibits AR translocation by reducing AR and HSP27 phosphorylation.
• Both C28 and SAL inhibit growth of tumor spheroids and CRPC xenografts in mice.
• Both SAL and C28 induce cellular senescence via DYRK1A and induction of CCNG2 in CRPC cells.
• C28 and SAL regulate distinctly DREAM complex target gene expression.

## Graphical abstract

**Figure:**
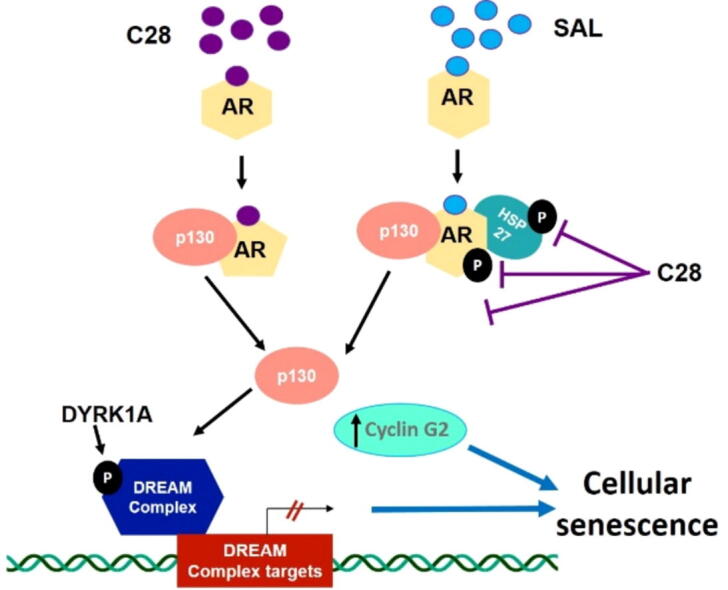

## Introduction

Prostate cancer (PCa) stands as one of the most prevalent malignancies among men and is the second leading cause of cancer-related deaths in Western countries [1]. The progression and development of PCa are primarily driven by androgen signaling mediated by the androgen receptor (AR) [2,3]. As a result, hormone therapy focuses on blocking this signaling pathway. This is typically achieved through androgen deprivation therapy, either alone or in combination with AR-antagonists, which reduce androgen levels and inhibit AR-signaling [4]. However, PCa frequently develops into a castration-resistant form but still relies on AR [[5], [6], [7]]. Therefore, targeting AR remains important in hormonally treating castration-resistant PCa (CRPC). Unfortunately, therapy resistance often arises, besides enhancing AR signaling also due to mutations in the AR ligand-binding domain (LBD) [[8], [9], [10]]. This challenge highlights the need for new AR-antagonists capable of effectively inactivating also AR mutants and impeding the progression of treatment-resistant PCa. Methyl 2-amino-5-chloro-3-idobenzoate, also known as C28, is a novel AR-antagonist with a novel chemical platform that we had introduced previously [11]. C28 effectively inactivates not only the wild-type AR but also those AR mutants that confer resistance to both first- and second-generation antagonists [11]. This suggests that C28 operates through another mode of antagonistic action compared to therapeutically used AR-antagonists.

Interestingly and paradoxically, supraphysiological androgen levels (SAL) using either DHT or the synthetic androgen R1881 potently inhibit PCa growth. This includes various PCa cell lines, tumor spheroids [12,13] or patients treated with SAL results also in suppression of PCa growth [14,15]. In line with this, bipolar androgen therapy (BAT) has emerged as a promising clinical approach, involving cycling serum testosterone levels between supraphysiological and near castrate levels using intermittent testosterone injection [16,17]. Recent clinical trials have demonstrated that BAT can suppress tumor progression and even re-sensitize tumors to AR-targeted therapies [[16], [17], [18]].

So far, C28 has been shown to inhibit the growth of the androgen-dependent PCa cell line LNCaP and induce cellular senescence [11]. However, the inhibitory effects of C28 on CRPC cells and CRPC tumors, as well as the underlying molecular mechanisms and pathways by which C28 inhibits tumor growth and induces cellular senescence remain to be elucidated.

In this study, we evaluated the activity of C28 across several cellular and molecular pathways in CRPC models and compared it to SAL activity that also induce cellular senescence [13]. The obtained evidence suggest that, in contrast to SAL, C28 reduces both the phosphorylation of AR at Serin 81 and HSP27, known to be required for nuclear translocation. On the other hand and similar to SAL, C28 suppresses growth of tumor spheroids derived from CRPC and xenografted CRPC tumors *in vivo*. Both SAL and C28 induce cellular senescence in spheroids as well as *in vivo* in xenograft tumors.

Here, we revealed two novel pathways that mediate cellular senescence used by both C28 and SAL. On one hand we identified the interaction between p130 and AR and activation of the DREAM complex pathway by both SAL and C28. On the other hand, both treatments induce the atypical growth inhibitory cyclin, Cyclin G2, to mediate cellular senescence. Interestingly, the knockdown or the treatment with a small molecule inhibitor of DYRK1A, a key kinase required for DREAM complex assembly, represses cellular senescence mediated by either C28 or SAL. Thus, here we suggest two novel pathways including DYRK1A and cyclin G2 are used in common that are activated by the AR-antagonist C28 and agonist at SAL in CRPC.

## Materials and methods

### Cell culture and treatments

The androgen-independent growing C4-2 cells, serving as a model for CRPC with high levels of AR which harbor the AR T878A mutation, were obtained from Thalmann et al. in 2007 [19]. This cell line was cultured in DMEM (Gibco Life Technologies) supplemented with 20 % F12 medium, 5 % fetal bovine serum (FBS), 1 % penicillin/streptomycin and 2.5 % HEPES 1 M (pH 7.5). The AR-negative PC3 cells and PC3-AR cells expressing the wild-type AR were cultured in RPMI (Gibco Life Technologies) supplemented with 10 % FBS, 1 % penicillin/streptomycin, 1 % sodium pyruvate and 2.5 % HEPES 1 M (pH 7.5). In addition, the 22Rv1 cell line was cultured in RPMI (Gibco Life Technologies) supplemented with 5 % FBS, 1 % penicillin/streptomycin, 1 % sodium pyruvate and 2.5 % HEPES 1 M (pH 7.5). The LNCaP Abl EnzaR cell line, obtained from Prof. Helmut Klocker [20], was cultured in RMPI Glutamax (Gibco Life Technologies) supplemented with 10 % dscFBS and 1 % penicillin/streptomycin. All used cell lines were incubated at 37 °C in 5 % CO_2_. For cell treatments, the following conditions were applied: 3 µM C28, 10 µM C28, 30 µM C28, 100 µM Atraric Acid (AA), 10 µM Darolutamide (Dar), 1 nM R1881 (previously defined as SAL) [13], 800 nM AZ191 (DYRK1A inhibitor, Hycultec, HY-12277) or 0.1 % DMSO as a solvent control.

### Immunofluorescence staining

After 72 h incubation with treatments, cells were washed with HBSS buffer (Gibco, 14025–050) and fixed with 4 % formalin for 15 min at room temperature (RT). Post-fixation, cells were washed, stained with wheat germ agglutinin (WGA), permeabilized with 0.2 % Triton X100 and blocked with 5 % normal goat serum for 1 h. Cells were then incubated with primary antibodies against AR (1:600, Millipore, #06–680) and secondary anti-rabbit IgG Alexa 546 antibody. Nuclei were stained by incubating the cells with Hoechst (1:10,000, Invitrogen, H3569). The coverslips were then mounted on glass slides using Fluoromount G (BIOZOL, SBA-0100–01). Images were captured using a Zeiss LSM 880 microscope. Quantification was performed using Fiji software [21].

### Protein extraction and Western blotting

Protein extraction and Western blotting were performed as described earlier [22]. In short, cells were lysed with lysis buffer containing proteinase and phosphatase inhibitors. Signal detection was performed by ImageQuant™ LAS 4000 (GE Healthcare Bio-Sciences AB) with ECL reagents (GE Healthcare). Band quantification was performed via the LabImage D1 software. The antibodies used are listed in supplementary Table S1.

### RNA extraction and qRT-PCR

qRT-PCR assays were performed as described earlier [13]. The primers used are listed in supplementary Table S2. The data plots show the mean of the expression levels with error bars representing the SEM.

### Co-immunoprecipitation

Co-immunoprecipitation (Co-IP) experiments followed an established protocol [22]. Briefly, either a specific antibody against AR or normal rabbit IgG (as a negative control) was incubated with protein A magnetic beads. After thorough washing, whole cell lysates from cells treated with DMSO, C28 or SAL were added to the antibody-bound beads and incubated at 4 °C for 2  h to capture protein–protein interactions. Following three to five washes with 1x PBS, the beads were resuspended in SDS buffer and boiled at 99 °C. The eluted protein complexes were separated on 12 % SDS-PAGE gels and detected by Western blotting. Details of the used antibodies are provided in supplementary Table S1.

### Growth assays and senescence-associated β-galactosidase (SA β-gal) staining

Experiments followed an established protocol [13,23,24]. For details see supplemental information.

### Tumor spheroid generation, SA β-gal activity staining and immunofluorescence assays

The spheroid formation assay was performed based on previous protocols [12,25]. In detail, 1000 C4–2 cells or LNCaP Abl EnzaR cells per well were seeded in 96-well ultralow attachment plates (PerkinElmer). The plates were centrifuged 3 times at 300  rpm for 3  min at RT to promote spheroid formation, followed by incubation at 37 °C, 5 % CO_2_. Slices were imaged using a bright field microscope CellObserverZ1 (Carl Zeiss). For Ki67 staining or AURKB detection, spheroid slices were permeabilized and blocked for 1 h at RT with 5 % normal goat serum (NGS) (Biozol; Germany; ENG9010-10), incubated with primary anti-Ki67 antibody or anti-AURKB antibody overnight and after washing with secondary anti-rabbit IgG Alexa 546 antibody for 1 h at RT in the dark. DAPI (Life Technologies; USA; H3569) solution (1 μg/ml in 1x PBS) was applied for 10 min to stain nuclei. Spheroid slices were mounted with Fluoromount-G® (SouthernBiotech; USA; 0100–01) and coverslips. Images were acquired using confocal laser scanning microscope (Carl Zeiss LSM 880). Quantification was conducted using Fiji software [21].

### Xenograft experiments

Approval for *in vivo* mouse experiments was obtained from Thüringer Landesamt für Lebensmittelsicherheit und Verbraucherschutz, Germany (Reg.-Nr.: UKJ-23–013). Statistical power analyses were performed to calculate the number of required mice [26]. To generate C4-2 xenografts, 10^6^ C4-2 cells per 50 μl 1x PBS, mixed with 1:1 Matrigel (CORNING; USA; 356231), were injected subcutaneously into both flanks of intact (non-castrated) nude mice (8-week-old male athymic nude mice, Janvier Labs, France). Tumor size was measured with a caliper every 48 h, beginning when tumors became visible. Tumor volumes were calculated using the formula: (length × width^2^) × 0.52). When the tumors reached a size of approximately 80 mm^3^, mice were randomly assigned to treatment groups and administered either vehicle (0.5 % Tween 80), C28 (100 mg/kg), or dihydrotestosterone (DHT, 50 mg/kg) corresponding SAL [[27], [28], [29]] (AbMol BioScience; USA; M6033) via daily subcutaneously injection. General body condition of mice is then observed daily after the start of subcutaneous injection of vehicle or AR-ligands. Mice were weighed every other day and euthanized when the tumor size reached about 800 mm^3^, weight loss surpassed 20 % of the initial weight, or after 5 weeks of treatment. After freezing tumors in liquid nitrogen, RNA and protein were extracted according to the protocol described previously [30]. Outliers identification and exclusion were performed using the ROUT method (Q = 1 %) with Graph Pad Prism 8.0. Two-way ANOVA and student *t*-test were conducted using Graph Pad Prism 8.0. Anti-tumor activity was calculated using the formula (ΔT/ΔC)*100 which is defined as: [(mean tumor volume in the treated group on final day − mean tumor volume in the treated group at start day of treatment)/(mean tumor volume in the vehicle control group on final day − mean tumor volume in the vehicle control group at start day of treatment)]*100 [31].

### In silico pharmacokinetic and toxicity predictions

The SMILES structure of C28 was generated using the CACTUS chemical identifier resolver and used as an input for further analysis. The PROTOX-3.0 webtool [32] was employed to predict the toxicity profile of C28 and pharmacokinetic properties were assessed using the SwissADME webtool [33].

### Hematoxylin-eosin (H&E) staining

For histological analyses H&E staining was performed on paraffin sections of mouse prostate using standard procedures. In brief, slices were stained in hematoxylin for 45 sec and washed with tap water for 10 min. The sections were then counterstained in alcoholic-eosin for 45 sec, dehydrated through an ascending ethanol series, cleared in xylene, and mounted with Entellan^TM^Neu (Sigma-Aldrich, Germany). Slices were imaged using a bright field microscope CellObserverZ1 (Carl Zeiss).

### Flow cytometry

40,000 cells were seeded and, after 48 h, treated with AR-antagonists, SAL, or DMSO for 3 days. Then incubated with 50 µl RNase A (100 µg/ml) and 200 µl propidium iodide solution (50 µg/ml) for 30 min in a dark room at RT. Fluorescence was measured using flow cytometry (BD ACCURI C6 plus), and 10,000 cells per sample were analyzed.

### Identifying gene network connection

To investigate the network connection between RBL2 and the targets of the DREAM complex Cytoscape [34] software was used. Network was generated by GenMANIA [35] from Cytoscape and STRING webtool [36]. CytoHubba [37] in Cytoscape was used for ranking calculation and visualization of network. MCC method was used for showing the ranking of the protein in the network. Darker color represents higher rank in the network.

### Transfection with siRNA

To modulate the expression of DYRK1A and CCNG2, the C4-2 cell line was subjected to knockdown using siRNA. ON-TARGETplus Human *DYRK1A* siRNA (Dharmacon; USA; L-004805-00-0005) at a final concentration of 25 nM, along with a negative control, ON-TARGETplus nontargeting control siRNA with the sequence ‘UGGUUUACAUGUUGUGUGA’ (Dharmacon; USA; D-001810-04-20) were used. Additionally, ON-TARGETplus Human *CCNG2* siRNA (Dharmacon; USA; L-003217-00-0005) was used for *CCNG2* knockdown. Transfection was performed using DharmaFECT reagent (Dharmacon; USA; T-2003-02) based on the manufacturer’s protocol, 24 h before treatment.

### Ligand-protein interaction modeling

The crystal structure of the AR ligand binding domain complexed with agonist was obtained from RCSB Protein Data Bank (PDB ID 1E3G). After removing the ligand, the protein was used for docking. The 3D structure of C28 was generated using the MolView web application and the 3D structure of Dar was obtained from PubChem (CID 67171867). Docking was performed using SwissDock, specifically the AutoDock vina section [38,39]. Visualization of results was carried out using PyMOL version 1.8.0.0.

### Statistical analysis

Graph Pad Prism 8.0 was used for statistical analysis. All results are presented as the mean ± SEM, based on at least two independent experiments, with N representing biological replicates and n representing technical replicates. The significance of the results was evaluated using either a two-tailed unpaired *t*-test or two-way analysis of variance (ANOVA). A p-value of less than 0.05 was considered significant. Significance levels were identified as follows: *p < 0.05; **p < 0.01; ***p < 0.001; ****p < 0.0001; ns: not significant.

### Data availability

The p130 ChIP-Seq, AR ChIP-seq and Rb ChIP-seq datasets [28] from C4-2 cells under accession number GSE179684, along with E2F1 ChIP-seq datasets [40] with accession number GSE154191 from C4-2 cells were obtained from Gene Expression Omnibus (GEO). Our RNA-seq datasets from C4-2 cells, and those from VCaP cells, C4-2 xenografts and PDX xenografts are accessible in GEO under accession numbers GSE172205, GSE148397, GSE179687, and GSE188174, respectively. The GEPIA dataset was used for correlation analysis. Kaplan-Meier survival plots were generated using data from human protein atlas database. TNM web tool, updated in June 2023, was utilized [41] to compare the expression of DREAM complex targets between normal and PCa patient samples.

## Results

### C28 inhibits AR translocation by downregulation of AR and HSP27 phosphorylation

**Fig. 1:**
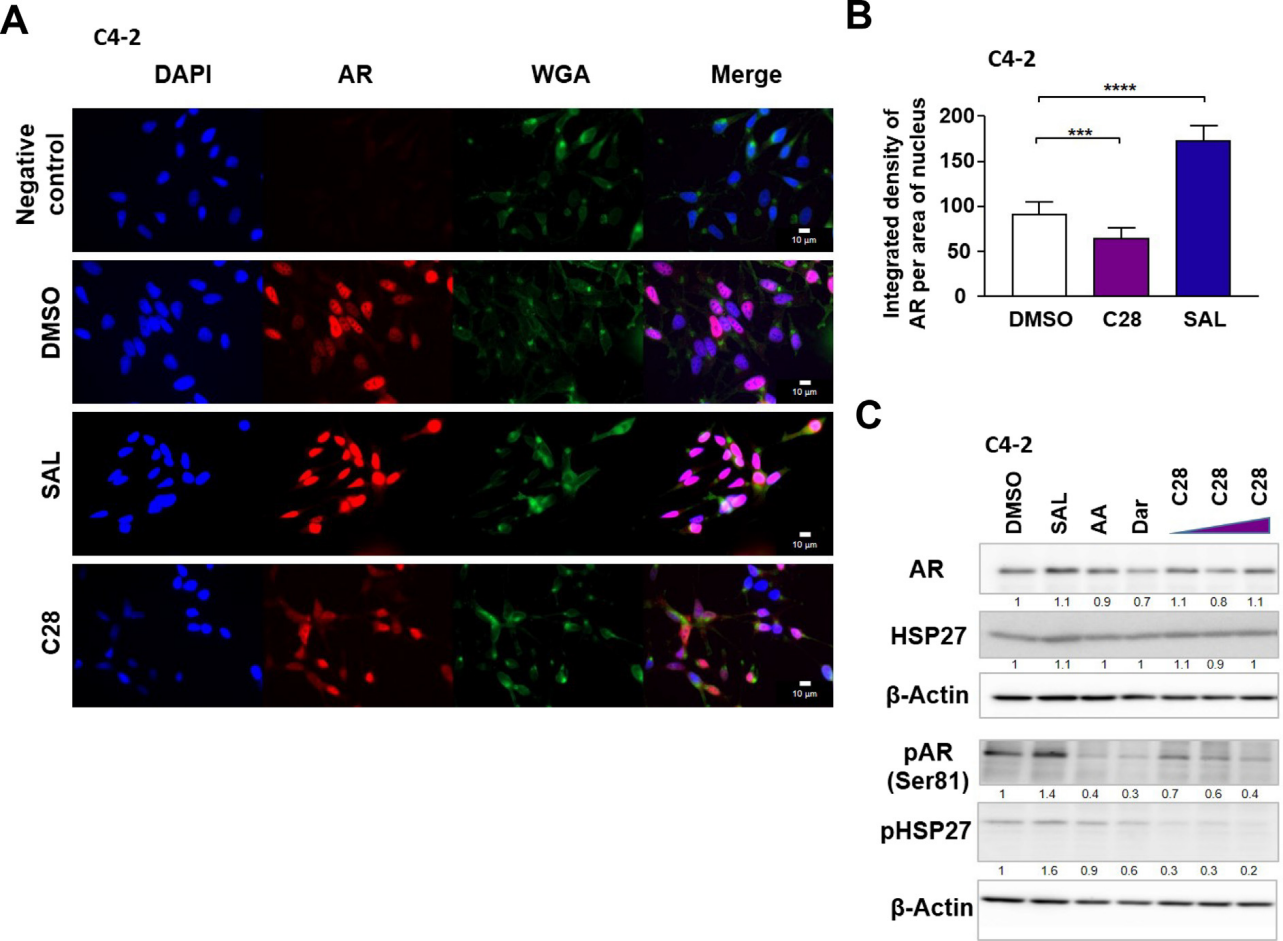

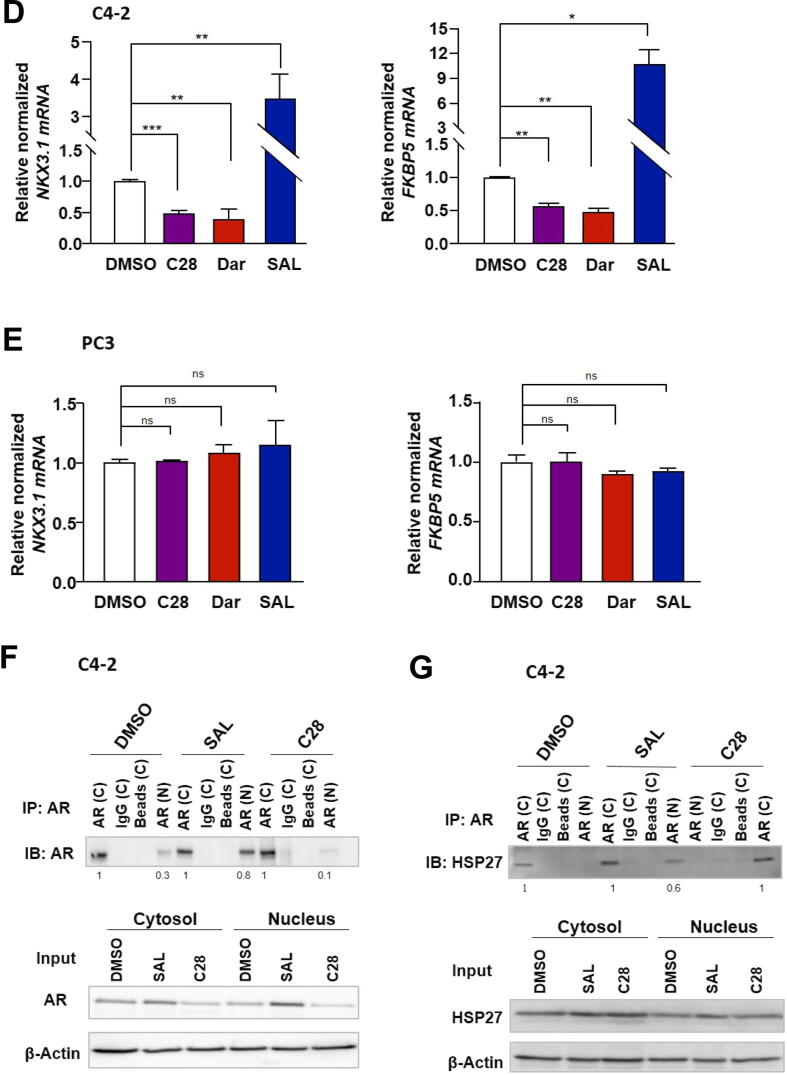
In contrast to SAL, C28 reduces AR nuclear translocation associated with inhibition of phosphorylation of both AR and HSP27. **A:** Immunofluorescence using anti-AR antibody to analyze the intracellular localization of AR (red) in C4-2 cells treated with SAL (1 nM R1881) [13], C28 (30 µM), or DMSO as solvent control. Samples without the primary antibody serve as a negative control. DAPI staining was used to detect nuclei (blue), anti-wheat germ agglutinin antibody is used to detect cell membrane (WGA, green). Scale bars represent 10 µm. **B:** Quantification of the integrated density of AR in the nuclei. Data are presented as mean ± SEM (n = 10 technical replicates). **C:** Western blot analysis of treated C4-2 cells with AR-antagonists with increasing concentrations of C28 (3, 10, and 30 µM) and SAL analyzing levels of AR, pAR (Ser81), HSP27, and pHSP27 with β-Actin that serves as loading control (N = 3). The numbers indicate the band intensities normalized to β-Actin and relative to DMSO control. **D** and **E:** mRNA levels of AR-target genes *NKX3.1* and *FKBP5* measured by qRT-PCR. D: C4-2 cells, bar graphs are shown as mean ± SEM from nine technical replicates (n = 9) of three independent experiments. E: PC3 cells, bar graphs are shown as mean ± SEM from six technical replicates (n = 6) of two independent experiments. *TBP* and *alpha-Tubulin* were used as housekeeping genes for normalization. **F:** Immunoprecipitation of AR and detection of AR. **G:** Co-Immunoprecipitation (Co-IP) of AR and detection of HSP27. Cytosol (C), Nucleus (N). (N = 3). Band intensities are shown relative to the cytosol. Statistical analysis was performed using a two-tailed unpaired Student *t*-test. (For interpretation of the references to color in this figure legend, the reader is referred to the web version of this article.)

Using transfected HeLa cells with a GFP-AR fusion indicated as a first evidence that C28 inhibits AR nuclear translocation [11]. However, whether this is also observed in CRPC cells with the endogenously expressed AR and which underlying molecular pathway is used by C28 to inhibit nuclear translocation remain unclear. To address this, immunofluorescence staining with an anti-AR antibody using C4-2 cells as an established model for CRPC that express endogenously the AR mutant T878A that mediates resistance to the AR antagonists flutamide and hydroxyflutamide [42], indicates that treatment with C28 inhibits significantly AR nuclear import (Fig. 1A, B). As negative control for immunofluorescence only the secondary antibody was used. Wheat germ agglutinin (WGA) for membrane detection and DAPI for nuclear staining were included. As negative control for ligand treatments DMSO was used as solvent control, and as positive control R1881 as the synthetic and less metabolizable androgen that, as expected, increased significantly nuclear translocation of AR (Fig. 1A, B).

For AR translocation the phosphorylation of both AR at serine 81 [43] and HSP27 [44,45] is required, which is also associated with PCa progression [46]. Therefore, we hypothesized that C28 interferes with phosphorylation of either HSP27. C4-2 cells were treated with various AR ligands including the AR-antagonists C28, Atraric acid (AA) or Darolutamide (Dar), and as androgen 1 nM R1881 (SAL) was used. DMSO is used as a solvent control. As expected and in line with enhanced translocation, SAL induces AR phosphorylation at Ser81. Interestingly, the phosphorylation levels of AR Ser81 (pAR) is decreased in the presence of C28 and by other antagonists. Also, the data suggest that the phosphorylation levels of HSP27 (pHSP27) are downregulated by C28 and the other employed AR-antagonist (Fig. 1C). Notably, the phosphorylation levels of both AR and HSP27 in the presence of C28 is reduced to a similar level observed with Dar, a well-established second-generation AR-antagonist for treatment patients with castration-resistant PCa [47]. This data suggests that C28 reduces the phosphorylation levels of both HSP27 and AR at Ser81 and can explain the observed reduced AR nuclear translocation.

Accordingly as expected, SAL treatment upregulates whereas AR-antagonists downregulate the mRNA levels of the direct positively-regulated AR target genes *NKX3.1, FKBP5*, *KLK3* encoding the diagnostic marker prostate specific antigen (PSA), and *TMPRSS2* (Fig. 1D and Fig. S1A). To further validate these findings, PC3-AR cells expressing the wild-type AR and also 22Rv1 cell line expressing the AR and the AR-V7 splice variant were utilized. Consistent with previous results, SAL treatment increased the mRNA levels of *FKBP5* and *NKX3.1* (Fig. S1B, E), while C28 treatment led to their downregulation (Fig. S1B, E). These results confirm that C28 inhibits AR transactivation in both AR T878A mutant and wild-type AR, aligning with the previous findings from our group showing that C28 suppresses the transactivation of AR point mutants overexpressed in transfected CV-1 cells [11]. As a negatively-regulated AR target gene being repressed by SAL treatment [12] *TERT* expression was analyzed in both C4-2 and PC3-AR cell lines. Interestingly, both AR-agonist and −antagonists reduce *hTERT* expression levels (Fig. S1C, D) suggesting that AR-antagonists do not purely inhibit all AR signaling rather AR-antagonists activate part of AR signaling such as cellular senescence.

To depict the AR specificity, the AR-negative PC3 cell line was employed, which showed neither an androgen-dependent induction of positively-regulated AR target genes nor transcriptional repression by C28 or Dar (Fig. 1E). Similarly, no response of treatment was observed for the expression of the known negatively-regulated AR target gene *hTERT* [12] in PC3 cells (Fig. S1F).

To determine whether there is an interaction between AR and HSP27 in the presence of C28 co–immunoprecipitation (Co-IP) experiments were performed. Protein extracts from treated C4-2 cells were immunoprecipitated with an anti-AR antibody and Western blotting was performed to detect HSP27 (Fig. 1F, G). In the presence of C28, the majority of AR protein was detected in the cytosolic fraction compared to control and SAL (Fig. 1F), while in the presence of SAL more AR was detected in the nuclear fractions. With both C28 and SAL treatments an AR-HSP27 interaction was detected in the cytosolic fraction. The data further suggest that C28 stabilizes the AR-HSP27 interaction in the cytosol and reduces their interaction in the nucleus compared to SAL (Fig. 1G), indicating that unlike C28, SAL promotes nuclear translocation of the AR-HSP27 complex. Taking together, these findings suggest that C28 inhibits nuclear translocation of endogenous AR in CRPC cells by reducing phosphorylation of both AR and HSP27 and stabilizing the AR-HSP27 complex in the cytosol.

### C28 represses growth and induces cellular senescence in CRPC 2D adherent cell culture

**Fig. 2:**
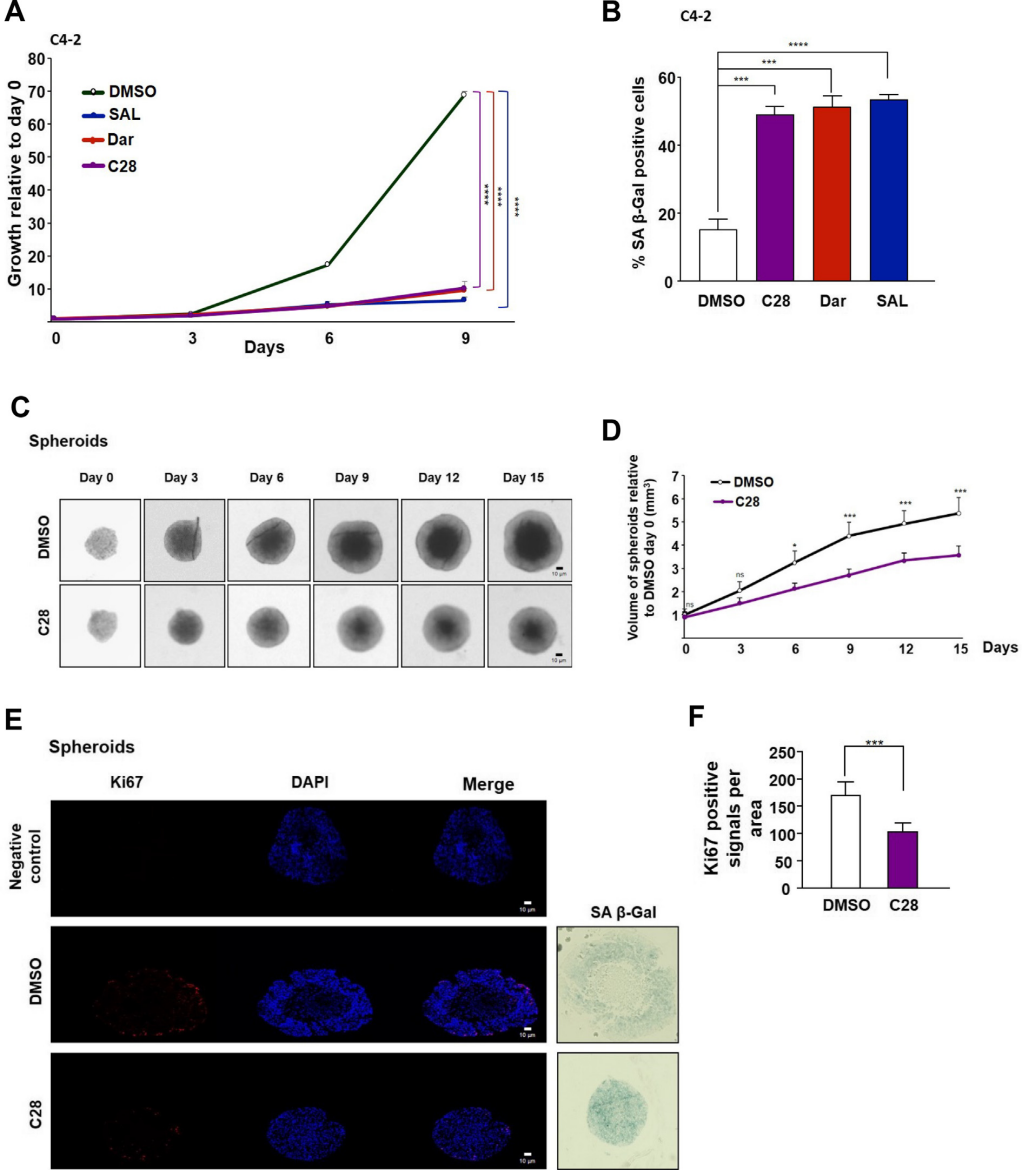

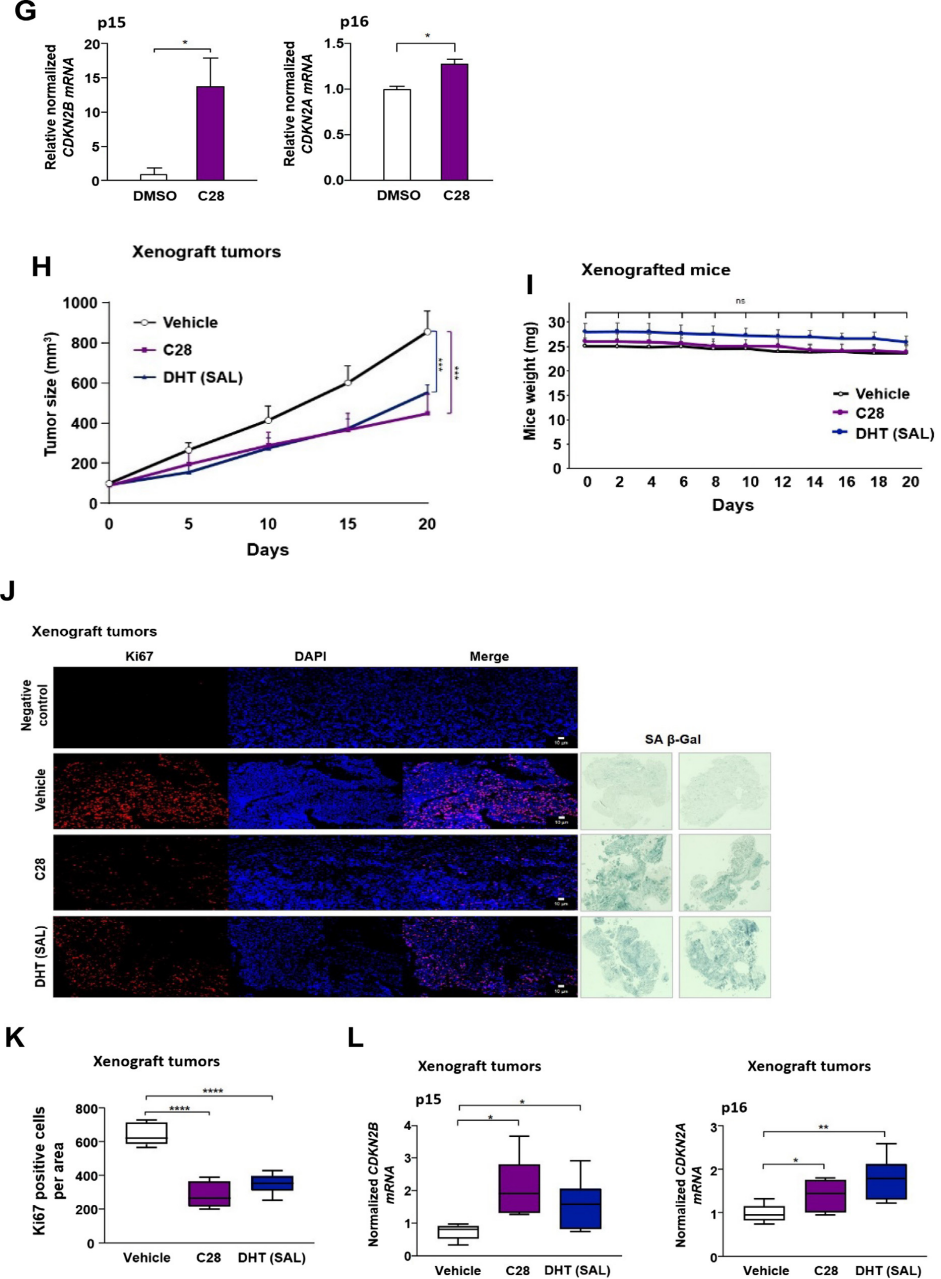
C28 induces cellular senescence and inhibits growth of CRPC adherent culture, 3D tumor spheroids and xenografted CRPC tumors in mice. **A:** 9 days growth curve of adherent C4-2 cells treated with AR-ligands. Crystal violet absorbance at OD 590 nm was normalized to day 0. Bar graphs are shown as mean ± SEM from four technical replicates (n = 4) of two independent experiments. **B:** Percentage of SA β-Gal positive C4–2 cells. Data are presented as mean ± SEM from twelve biological replicates (n = 12) of three independent experiments. **C:** Generation and treatment of C4-2 tumor spheroids with C28 (30 µM) or DMSO as a solvent control for 15 days. Scale bars indicate 10 µm. **D:** Reduction of tumor spheroid volume by C28 treatment. Bar graphs are shown as mean ± SEM from twenty technical replicates (n = 20) of two independent experiments. **E:** Ki67 immunofluorescence staining, as a proliferation marker, and SA β-Gal activity staining as a cellular senescence marker in C4-2 spheroids. Samples without primary antibody serve as a negative control. Scale bars indicate 10 µm. **F:** Quantification of Ki67 positive signals per area. Bar graphs are shown as mean ± SEM from ten technical replicates (n = 10) of two independent experiments. **G:** qRT-PCR analysis of *CDKN2B* and *CDKN2A*, encoding p15^INK4b^ and p16 ^INK4a^, respectively in C4-2 spheroids from six technical replicates (n = 6) of two independent experiments. **H:** Growth responses of C4-2 xenografts in mice to a 20-day course of C28 (100 mg/kg/day, N = 6), DHT (SAL, 50 mg/kg/day, N = 7), or vehicle control (0.5 % Tween 80, N = 5). Tumor volume was measured during treatment, showing growth inhibition by C28 and DHT (SAL). **I:** Mice body weight was measured every two days during the treatment period. **J:** Ki67 and SA β-Gal staining of C4-2 xenograft tumor slices from C28, DHT (SAL), or vehicle-treated mice. Scale bars indicate 10 µm. **K:** Quantification of Ki67 positive signals per area in CRPC xenograft tumors. **L:** qRT-PCR analysis of *CDKN2B* and *CDKN2A* in xenograft tumors treated with C28 (N = 6) or DHT (N = 7) compared to vehicle control (N = 5). *TBP* and *alpha-Tubulin* were used as housekeeping genes for normalization in all qRT-PCR analysis. Two-way ANOVA was used for analyses of growth curves and volume of the tumors, and two-tailed unpaired Student *t*-test was used for comparing treatments with the control. (For interpretation of the references to color in this figure legend, the reader is referred to the web version of this article.)

To investigate whether C28 inhibits the growth of CRPC cell line, adherent C4-2 cells were treated for 9 days with the AR-ligands C28, Dar, or R1881 at SAL. Growth analyses indicate that C28 inhibits growth of CRPC cells (Fig. 2A and Fig. S2A). Notably, C28 suppresses CRPC cells growth as potently as Dar. Since SAL was shown previously to induce cellular senescence [12,13,48], the SA β-Gal activity staining of treated C4-2 cells suggests that C28 and Dar also induce cellular senescence within 72 h of treatment (Fig. 2B and Fig. S2B).

Treating the AR-negative PC3 cells indicates neither growth inhibition nor induction of cellular senescence following treatment C28 suggesting that C28 targets specifically the AR. Similarly, no significant changes of growth or induction of cellular senescence were observed when PC3 cells were exposed to Dar or SAL (Fig. S2G, H). However, in PC3-AR cells, which express wild-type AR, treatment with C28 and SAL resulted in significant growth inhibition and induction of cellular senescence (Fig. S2J, K). Notably, both treatments led to the upregulation of *CDKN2B* as a senescence marker, encoding p15^INK4b^ (Fig. S2L). These results further support the notion that C28 suppresses AR-driven CRPC growth while inducing senescence. In line with induction of cellular senescence in C4-2 cells, C28 upregulates both the mRNA and protein levels of p15^INK4b^ and p16^INK4a^, as markers of cellular senescence (Fig. S2C, D and Fig. S2E, F) to a similar extent as observed by Dar treatment. These results suggest that C28 effectively inhibits the growth of C4-2 cells and induces cellular senescence, similar to Dar and SAL in CRPC cells. In contrast, in PC3 cells p15^INK4b^ mRNA levels remained unchanged in response to these AR-ligands (Fig. S2I). These findings indicate the measured cellular responses by C28 and Dar are AR-dependent.

Interestingly, while both C28 and SAL enhanced the percentage of SA β-Gal stained in 22Rv1 cells, there was no significant impact on cell proliferation (Fig. S2M, N). Furthermore, in contrast to C4-2 and PC3-AR cells, p15^INK4b^ mRNA levels remained unchanged in 22Rv1 cells following treatment (Fig. S2O). These findings suggest that presence of AR-V7 may contribute to resistance against C28 and SAL.

### C28 suppresses proliferation and induces cellular senescence in CRPC 3D tumor spheroids and CRCP xenograft tumors in mice

A 3D cell culture systems offers a better model system for tumor complexity compared to adherent cell culture [49]. Therefore, to verify the inhibitory effects of C28, PCa spheroids were generated from C4–2 cells and treated with C28 or DMSO as solvent control. Similar to the result observed for adherent 2D cultures, C28 significantly reduced spheroid growth (Fig. 2C, D). Accordingly, the detection of the proliferation marker Ki67 by immunofluorescence showed a significant reduction in Ki67 positive cells following C28 treatment (Fig. 2E, F). This inhibition of proliferation was corroborated by increased senescent cell levels (Fig. 2F) as well as upregulation of p15^INK4b^ and p16^INK4a^ mRNA levels in C28–treated spheroids (Fig. 2G). Expression analysis of the direct AR target genes *NKX3.1* and *FKBP5* in C28-treated spheroids confirmed the repression of AR-transactivation (Fig. S3A). Also, in line with the adherent cell culture, the *hTERT* mRNA was repressed also in spheroid model by C28 (Fig. S3A). In addition, to assess whether C28 inhibits the growth of an enzalutamide-resistant model, LNCaP Abl EnzaR spheroids were employed, which present castration-resistant cells being also resistant to enzalutamide. Treatment with C28 failed to inhibit spheroid growth or induce cellular senescence (Fig. S4A-C). The mRNA levels of p15^INK4b^ also remained unchanged upon C28 treatment (Fig. S4D) indicating lack of cell senescence induction. These results suggest that enzalutamide resistant CRPC cells are also resistant against C28 treatment.

To extend these findings, C4-2 xenograft mice model with subcutaneous tumors [30] was used to verify whether C28 inhibits growth of CRPC *in vivo* and whether cellular senescence can be induced by C28 *in vivo* in tumors. For that purpose, mice were mock-treated, treated with C28 (100 mg/kg/day), or DHT (50  mg/kg/day) as SAL previously established in multiple studies [[27], [28], [29]]. The C28 dosage was selected based on a comparable dose previously used for AA, a natural AR-antagonist, for treatment of C4-2 xenografted mice [30] and Dar for VCaP xenografted mice [50]. Both C28 and SAL treatments inhibit C4-2 tumor growth in mice (Fig. 2H). The calculated relative growth rate corresponding to antitumor activity [31] of both treatments is depicted in the supplementary figure S3C. Importantly, mice body weight was unaffected during treatment (Fig. 2I), suggesting that C28 and SAL may not have measurable side-effects. In line with this, *in silico* analysis of C28 is predicted to have favorable pharmacokinetic properties and low toxicity risks. C28 exhibits favorable oral bioactivity, good absorption, and low skin permeability. C28 is not predicted to inhibit CYP1A2, CYP2C19, CYP2D6, or CYP3A4 suggesting a reduced likelihood of drug-drug interactions via these enzymes (Table S3). However, the inhibition of CYP2C9 could lead to metabolic interactions. Toxicity analysis using PROTOX-3.0 classifies C28 to have moderate to mild toxicity with potential neurotoxicity due to its ability to cross the blood–brain barrier. Notably, C28 is predicted not to exhibit any cardiotoxicity, immunotoxicity, cytotoxicity or mutagenicity.

Ki67 staining in tumor samples was reduced in tumors of C28- or SAL-treated mice, and enhanced SA β-Gal staining being in accordance with the reduced tumor growth (Fig. 2J, K). This finding was further supported by the significant upregulation of *CDKN2B* and *CDKN2A* encoding the cell cycle inhibitors p15^INK4b^ and p16^INK4a^, respectively, in the xenograft tumors (Fig. 2L).

Similar to the adherent cells and tumor spheroids, C28 treatment reduced the mRNA levels of *NKX3.1*, *FKBP5,* and *hTERT* in xenograft tumors (Fig. S3B). As expected, SAL treatment upregulated *NKX3.1* and *FKBP5* whereas *hTERT* transcription was downregulated (Fig. S3B), consistent with previous observation (Fig. S1C).

In addition to CRPC tumors, various mouse organs were analyzed including mouse prostate, testes, livers, kidneys and the levator ani muscles. Of note, C28 did not alter the weight of the androgen-responsive levator ani muscle [51] (Fig. S3D) suggesting C28 may not act as an AR-antagonist in these muscles. The analysis of mouse prostate morphology using H&E staining demonstrated no alterations in nuclear or cellular architecture between vehicle control and C28-treated groups (Fig. S3E) indicating that C28 does not induce significant histological changes in the prostate tissue. This observation further supports the absence of major side effects associated with C28 treatment.

To analyze the action of C28 on AR signaling in mouse tissues, the expression of known mouse AR target genes in various mouse organs was analyzed. In the kidney, the expression of the glucuronidase gene (*Gus*) was not significantly affected by C28 treatment, while SAL treatment significantly upregulated *Gus* expression (Fig. S3F). In the prostate, probasin (*Pbsn*) and sex determining region Y-box 9 (*Sox9*) levels were unaffected by C28, similar to SAL treatment (Fig. S3G). The expression of glutathione S-transferase pi 1 gene (*Gstp1*) in the liver (Fig. S3H) and hydroxysteroid dehydrogenase 3 beta-6 (*Hsd3b6*) or reproductive homeobox 5 (*Rhox5*) in testis (Fig. S3I) was not significantly affected but trended towards a decrease with C28 treatment. Similar results were observed for SAL treatment, although *Hsd3b6* expression was significantly downregulated by SAL (Fig. S3I). These results suggest that C28 effectively inhibits tumor growth, induces cellular senescence and mildly modulates AR targets expression in both 3D tumor spheroid models and xenograft tumors with low detected side-effects of AR target genes in mice.

### SAL preferentially arrests cells at G1/S and C28 at G1/S/G2

To investigate which phase of cell cycle is inhibited by C28, flow cytometric analysis of the cellular DNA content using PI staining was performed. When AR-antagonists or AR-agonist at SAL were applied, a higher proportion of cells were observed in G1 phase compared to DMSO, indicating that all employed AR-ligands cause a cell cycle arrest at G1 to S phase transition (Fig. S5A, B). Similar observation were obtained using C28 indicating a G1/S arrest. Notably, C28 treatment increased the percentage of the cells in the S phase, accompanied by a decrease in the number of cells in G2 phase (Fig. S5A). This suggests that C28 also induces cell cycle arrest at S/G2 phase transition. While SAL and Dar induce predominantly a G1/S arrest, these findings indicate that C28 induces cell cycle arrest at G1/S/G2 phase transitions.

### Expression levels of p130 is increased by C28

**Fig. 3:**
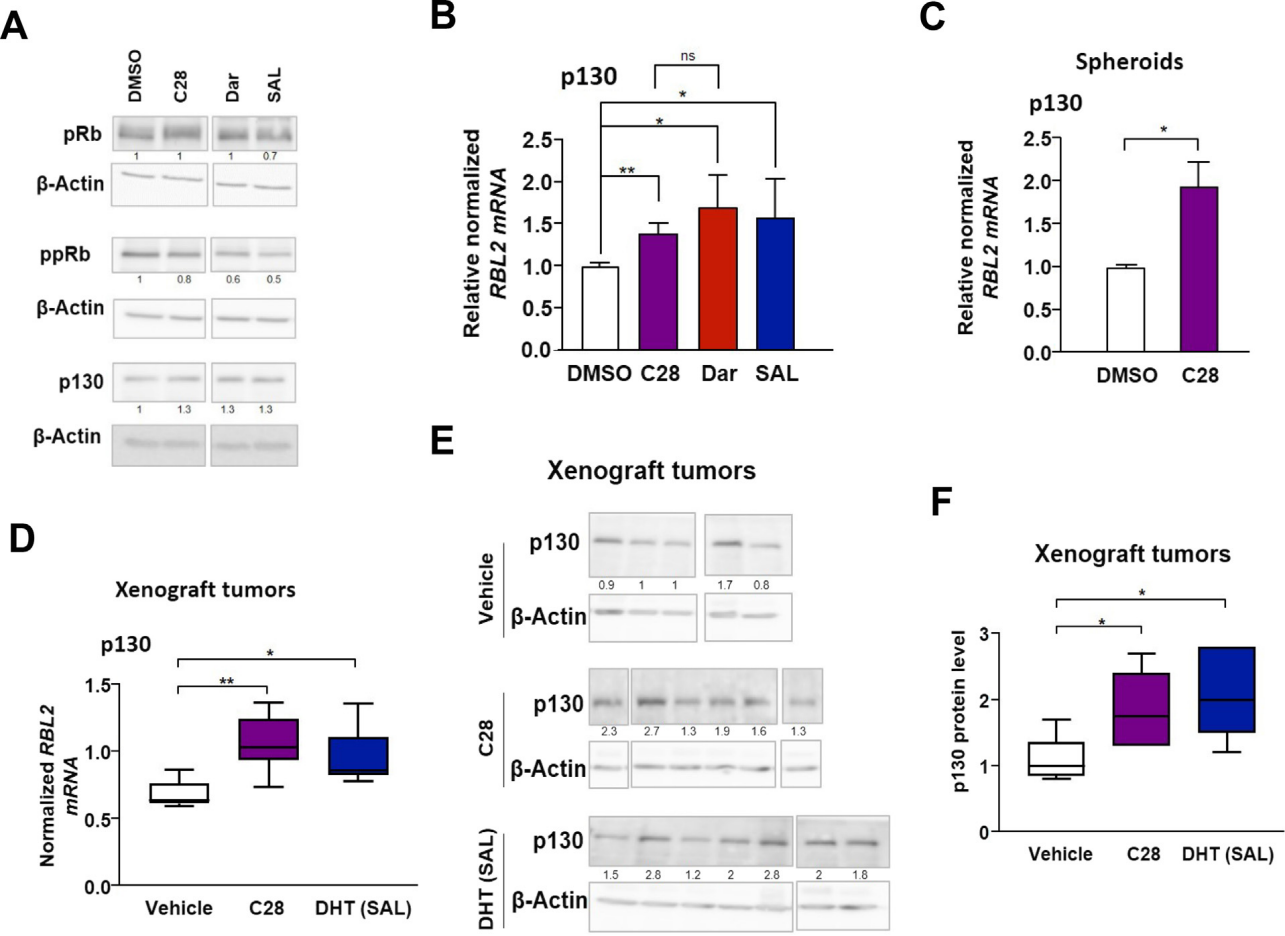

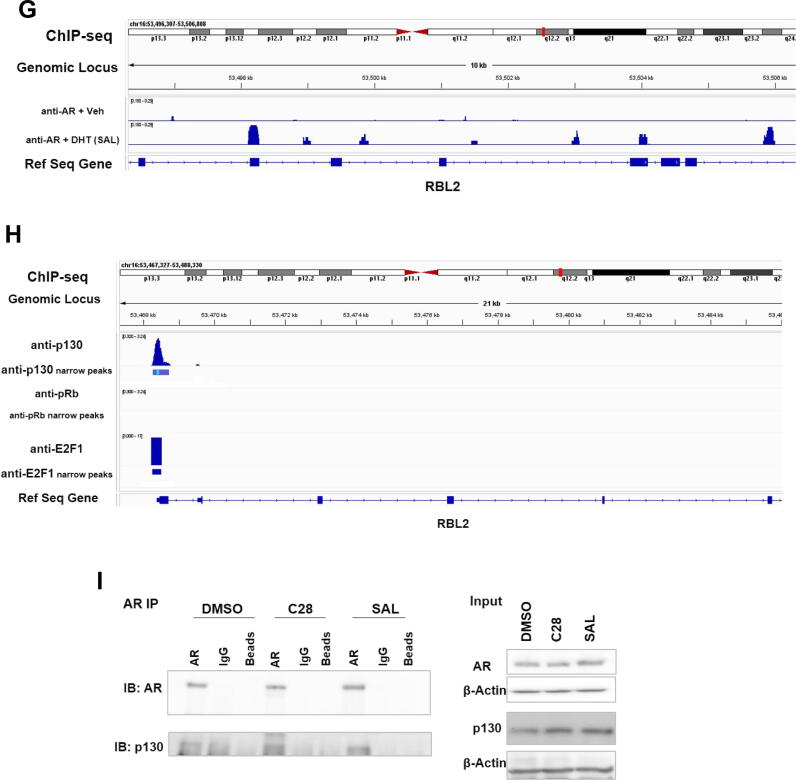
Both C28 and Sal upregulate p130 levels. **A:** Western blot analysis of pRb, phospho-pRb (ppRb) and p130 in treated C4-2 cells (N = 3). The expression was normalized to β-Actin as loading control. Numbers indicate normalized band intensities relative to DMSO control. **B:** qRT-PCR examination of *RBL2* expression, encoding p130, in CRPC adherent cultures treated with AR-ligands. Bar graphs are shown as mean ± SEM from nine technical replicates (n = 9) of three independent experiments. **C**: qRT-PCR analysis of *RBL2* expression in CRPC 3D spheroids treated with C28 or DMSO from six technical replicates (n = 6) of two independent experiments. **D:** qRT-PCR of *RBL2* expression in xenograft tumors of mice treated with C28 (N = 6), DHT (SAL, N = 7), or vehicle control (N = 5). *TBP* and *alpha-Tubulin* were used as housekeeping genes for normalization. **E:** Protein levels of p130 in xenograft tumors were analyzed by Western blotting. Numbers show band intensities normalized to β-Actin relative to the vehicle control. **F:** Protein levels are represented as mean ± SEM values. **G:** ChIP-seq data indicates AR occupancy at *RBL2* in C4-2 cells. **H:** ChIP-seq data indicates p130 and E2F1 occupancy at the regulatory regions of *RBL2* in C4-2 cells. IGV software was used for visualization. **I:** AR-p130 Co-IP immunoblot of lysates from AR and IgG immunoprecipitations using protein extracts from C4-2 cells treated with C28, SAL or DMSO for 72 h. A two-tailed unpaired Student *t*-test was used for comparing each treatment with the control.

Previously we reported that SAL treatment induces hypophosphorylation of pRb [13]. To analyze factors involved in cellular senescence regulated by C28, the level of pRb protein and its hyperphosphorylation level (ppRb), were analyzed in cell extracts. Both SAL and Dar strongly reduce ppRb levels, whereas C28 leads to a lesser reduction in hypophosphorylation of pRb (Fig. 3A). This promoted the hypothesis that C28 might induce cellular senescence possibly through another member of the Rb-like pocket proteins, such as p130 [52]. The analysis revealed that both protein (Fig. 3A and Fig. S6A) and mRNA levels of p130 (*RBL2*) were increased in the presence of C28 (Fig. 3B) supporting our hypothesis. Interestingly, the results further indicate that both AR-antagonists, Dar and C28, enhance p130 levels to a similar extent as well as the AR-agonist enhance p130 protein and its mRNA levels (Fig. 3A, B). Further analysis in PC3-AR cells revealed that both C28 and SAL upregulated *RBL2* mRNA levels (Fig. S6B). However, *RBL2* expression remained unchanged in 22Rv1 cells and LNCaP Abl EnzaR spheroids (Fig. S6C, D) correlating with the lack of growth inhibitory effects observed in these two cell lines. In PC3 cells, the expression levels of *RBL2* did not significantly alter by C28, Dar or SAL treatment (Fig. S6E) indicating the AR-dependent regulation of this gene. Notably, analyzing patient PCa samples retrieved from GEPIA dataset revealed a strong and significant positive correlation between *AR* and *RBL2* expression levels (Fig. S6F). This correlation suggests that the expression of these genes is indirectly or directly co-regulated in PCa. The correlation analysis between *AR* and *FKBP5*, as well as between *AR* and *NKX3.1*, as positive controls, also indicates positive correlations in PCa samples (Fig. S6G, H).

Further analysis of *RBL2* levels in C4-2 tumor spheroids and xenograft tumors indicate that C28 upregulates *RBL2* mRNA levels in both tumor spheroids and CRPC tumors *in vivo* (Fig. 3C, D). Also at protein level p130 was significantly enhanced by C28 and SAL in xenograft tumor samples (Fig. 3E, F). These data suggest that C28 enhances the p130 pocket domain protein levels. Interestingly, our AR ChIP-seq from LNCaP and ChIP-seq analysis of C4-2 cells suggests that AR is recruited to the p130 gene loci (Fig. 3G and Fig. S6I) indicating a possible mechanism for upregulation of *RBL2* by SAL. p130 itself is known to repress target genes by binding to E2F4 and enhance gene expression by binding to E2F1 [53]. Analyzing ChIP-seq datasets of p130 [28] and E2F1 [40] reveals that interestingly both factors are recruited to the same genomic locus of the *RBL2* promoter, indicating p130 may bind to its own gene loci through E2F1 (Fig. 3H). This data suggest an auto-regulatory mechanism by which p130 regulates its own expression likely through an interaction with E2F1.

Next, we investigated whether there is a protein–protein interaction between p130 and AR. Using Co-IP assays, p130 was detected in cell protein extracts with immunoprecipitated AR under DMSO, C28 or SAL treatments (Fig. 3I). The intensity of the p130 band was higher in the presence of C28 or SAL compared to DMSO suggesting that either C28 or SAL treatment stabilize the interaction between p130 and AR. Taken together, the results propose that C28 and SAL enhance p130 protein levels and protein–protein interaction of AR with p130.

### DREAM target genes are downregulated by C28 *in vitro* and *in vivo*

p130 is a constituent of the DREAM protein complex (Dimerization partner, RB-like, E2F and MUVB) [54], that functions as a transcriptional repressor of both G1/S and G2/M cell cycle regulatory genes [55]. Previous studies demonstrated that the DREAM complex regulates cell cycle by repressing genes controlling cell cycle progression and mitosis, such as *ATAD2*, *AURKB*, *CCNA2*, *MYBL2* and *FOXM1* [56]. In line with this, Kaplan-Meier survival plots obtained from the human protein atlas database indicate that high expression of these genes in PCa is associated with reduced survival (Fig. S7A). Moreover, the expression of these genes is significantly upregulated in prostate adenocarcinoma patient samples (n = 498) compared to normal samples (n = 204) [41] (Fig. S7B). Analyzing ChIP-seq dataset [28] suggests that p130 specifically binds to these DREAM complex target gene loci (Fig. S8), while no recruitment of pRb was observed [28].

Furthermore, the network connections between RBL2 and DREAM complex targets were examined using the cytoHubba from Cytoscape, and the STRING [34,35,37]. The data reveal that RBL2 and DREAM complex targets are interconnected through multiple ways and are also linked to cell cycle regulators and chromosome condensation factors (Fig. S9).

**Fig. 4:**
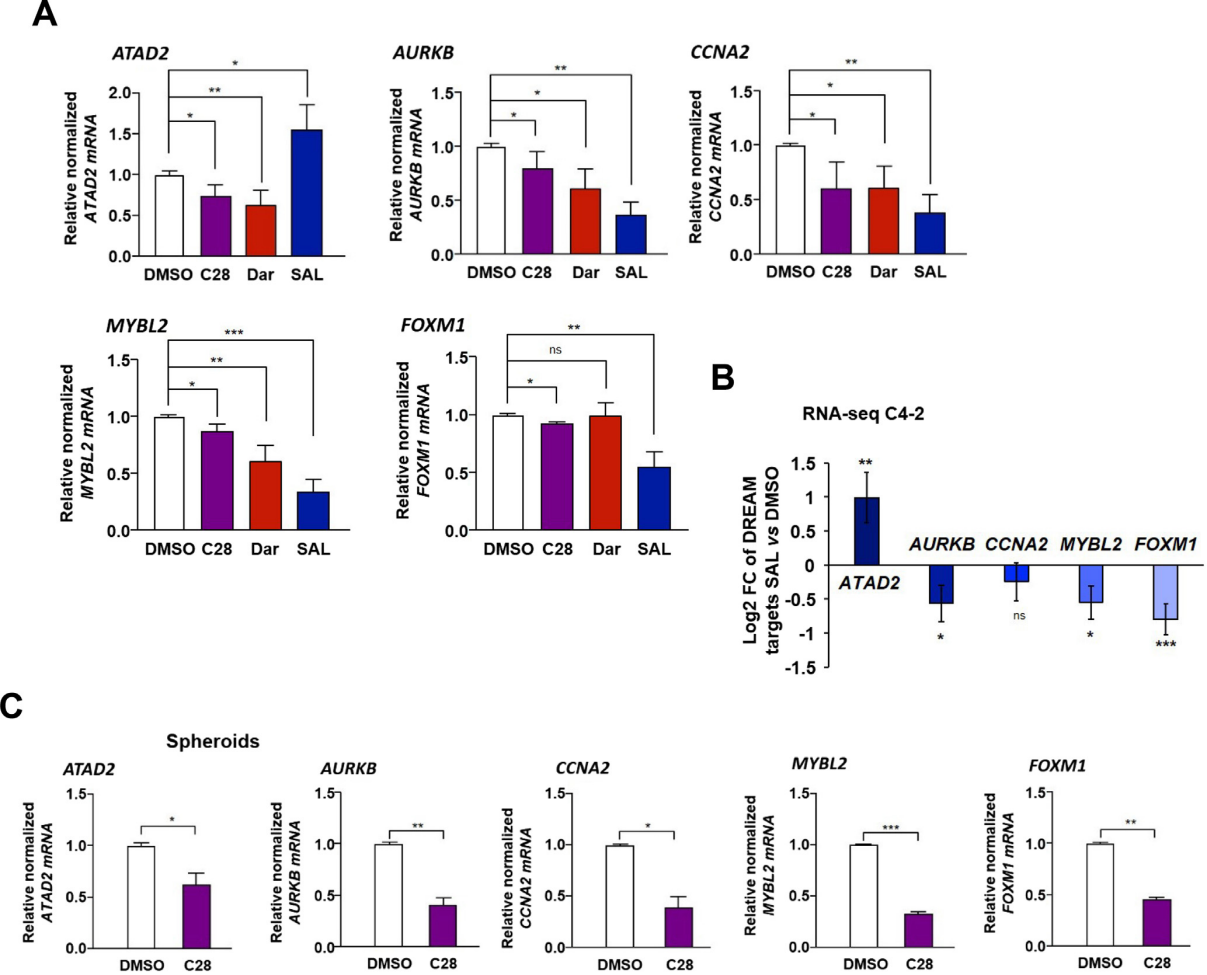

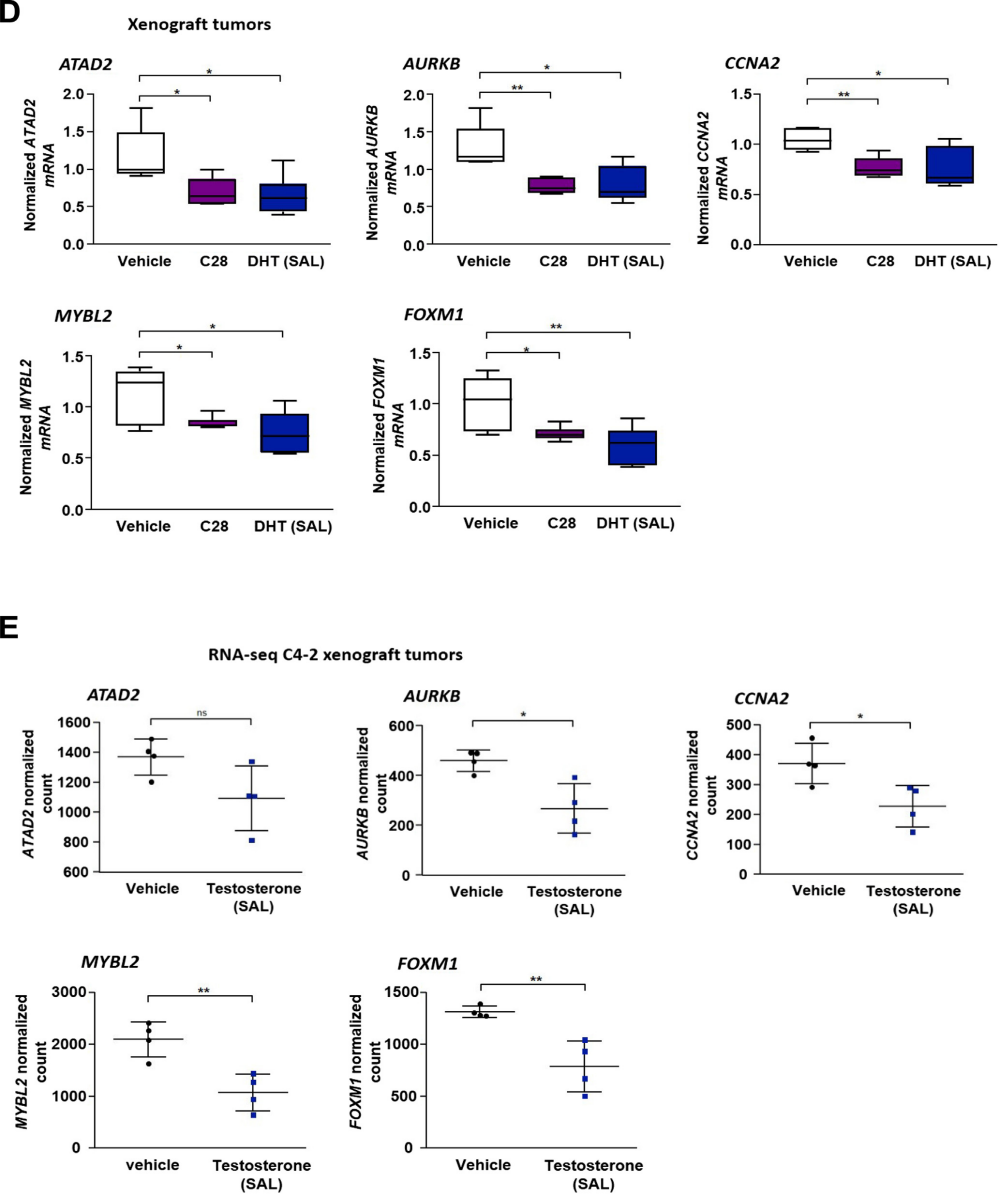

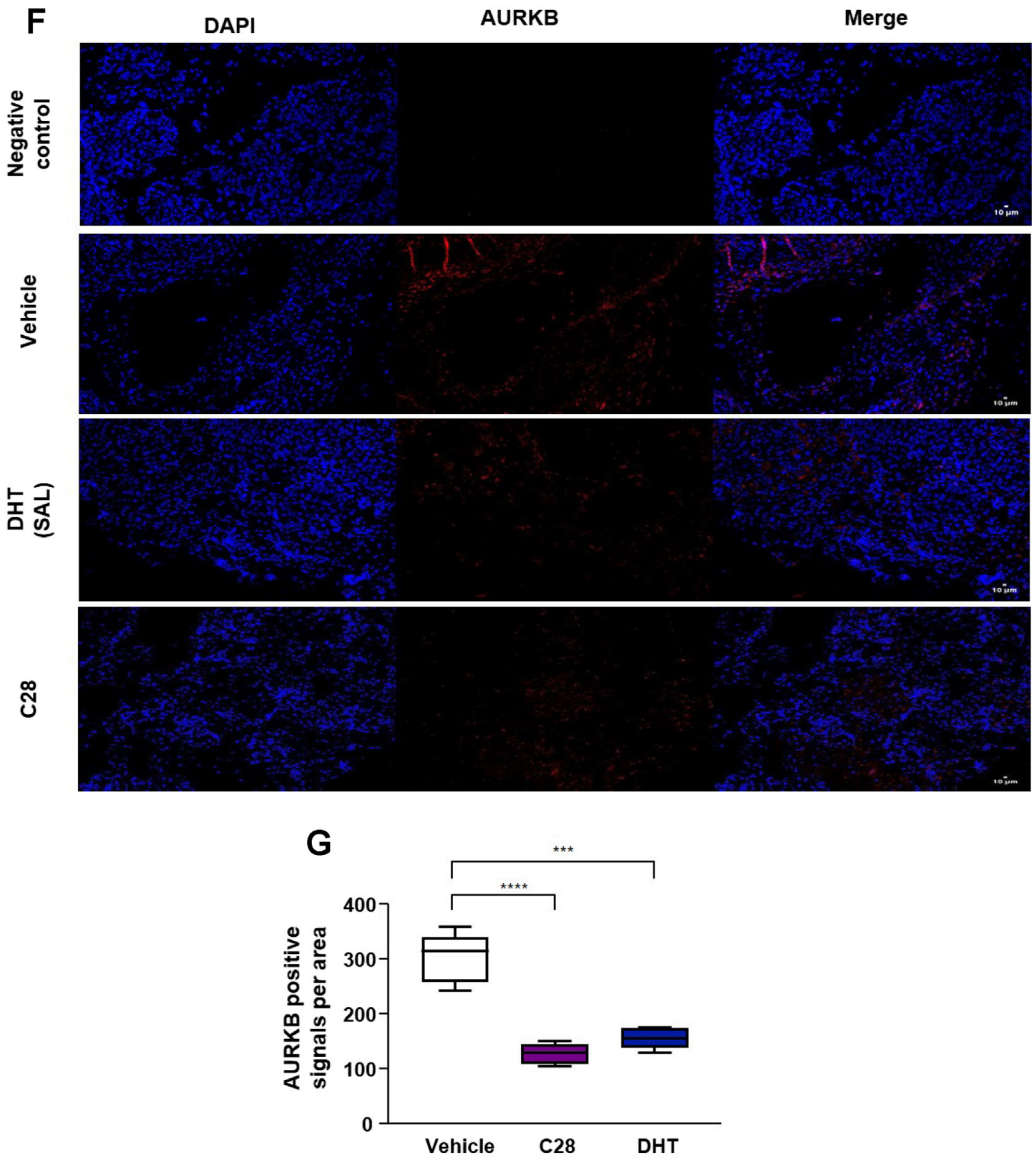
Both AR-agonist and −antagonists downregulate DREAM complex target genes. **A:** qRT-PCR analysis of the DREAM protein complex regulated genes *ATAD2*, *AURKB*, *CCNA2*, *MYBL2* and *FOXM1* in C4-2 cells treated with C28 (30 µM), Dar (10 µM), SAL or DMSO for 72 h. Bar graphs are shown as mean ± SEM from nine technical replicates (n = 9) of three independent experiments. **B**: RNA-seq gene expression analysis of DREAM complex gene targets in C4-2 cells treated with SAL. **C:** qRT-PCR analysis of the selected genes expression in C4-2 spheroids from six technical replicates (n = 6) of two independent experiments. **D:** qRT-PCR determination of DREAM complex target mRNA levels in C4-2 xenograft tumors treated with C28 (N = 6), DHT (SAL, N = 7), or vehicle control (N = 5). *TBP* and *alpha-Tubulin* were used as housekeeping genes for normalization. **E:** RNA-seq gene expression analysis of DREAM complex targets in C4-2 xenograft tumors treated with vehicle or SAL. All analyzed genes are significantly downregulated (*p < 0.05*), except *ATAD2* which shows a downward trend with *p =* 0.06. **F:** AURKB immunofluorescence staining of C4-2 xenograft tumor slices from C28, DHT (SAL), or vehicle-treated mice, with N = 5 for each treatment group. Scale bars indicate 10 µm. **G:** Quantification of AURKB positive signals per area in CRPC xenograft tumors. A two-tailed unpaired Student *t*-test was used for comparing each treatment with the control.

Based on induced p130 levels in C4-2 and PC3-AR cells by treatment with C28 or SAL, we hypothesized that the signaling by the DREAM complex is activated by AR agonists and antagonist. Therefore, the expression of DREAM complex target genes was analyzed following AR-antagonists and −agonist treatments. In line with our hypothesis, exposure of cells to C28 decreased the mRNA levels of *ATAD2*, *AURKB*, *CCNA2*, *MYBL2* and *FOXM1* (Fig. 4A). Similarly, Dar treatment significantly downregulated the expression of the selected DREAM complex targets, except *FOXM1*. Also, SAL inhibits the expression of most of these DREAM target genes. However, since ATAD2 was shown to be a direct and positively regulated AR target gene [57], as expected, SAL upregulates *ATAD2* mRNA levels in 2D adherent cells as well as in 3D tumor spheroids (Fig. 4A and Fig. S10A, B). This trend was recapitulated in PC3-AR cells, where C28 downregulated the expression levels of both *ATAD2* and *AURKB* whereas SAL upregulated the mRNA levels of *ATAD2* but repressed *AURKB* (Fig. S11A). Furthermore, *CCNA2* expression decreased upon both C28 and SAL treatment, but this reduction did not reach statistical significance (Fig. S11A). In 22Rv1 cells and LNCaP Abl EnzaR spheroids following C28 or SAL treatment no significant changes in the mRNA levels of these genes were observed in contrast to C4-2 and PC3-AR cells (Fig. S11C, D) indicating lack of transcriptional regulation of these genes by C28 and SAL. Analyzing published RNA-seq datasets from SAL-treated VCaP cells [58] also showed significant upregulation of *ATAD2* and downregulation of *AURKB* and *CCNA2* (Fig. S12A) confirming our observations. Moreover, re-analyzing our RNA-seq transcriptome from C4-2 cells [30] confirms significant downregulation of DREAM targets by SAL (Fig. 4B). These results suggest that also androgen treatment at SAL activates the DREAM signaling leading to repression of cell cycle promoting genes of the G2/M phase transition.

In summary, these findings suggest that both SAL and C28 regulate p130 levels and activate the DREAM complex, thereby negatively regulate cell cycle genes. This indicates a common pathway between AR-antagonist and −agonist to anastomose in activating the DREAM protein complex and aligns with the observation that both, C28 and SAL, induce cellular senescence. This observation also suggests that AR-antagonists do not completely inactivate the AR but activate a specific AR signaling.

The downregulation of DREAM target genes expression was also observed in tumor spheroids (Fig. 4C) and C4-2 xenograft tumors following C28 treatment (Fig. 4D). In line with this, in our xenograft tumors and published xenograft RNA-seq data [28], SAL reduced the mRNA levels of *AURKB*, *CCNA2*, *MYBL2* and *FOXM1* (Fig. 4D, E). Interestingly, in xenograft tumor samples, SAL treatment downregulated *ATAD2* mRNA levels (Fig. 4D), which is contrary to the result from the 2D culture (Fig. 4A, B) and may be due to a prolonged treatment of mice, the use DHT which is metabolized rapidly and its metabolites can activate the estrogen receptor beta [59], or a potential influence of the tumor microenvironment. A downward trend in the expression of DREAM targets was also observed by analyzing published RNA-seq dataset from patient-derived xenografts (PDX) [29] (Fig. S12B). To further validate the downregulation of DREAM complex target genes at protein level, AURKB as one of the key targets was analyzed. Immunofluorescence staining in C4-2 tumor spheroids revealed a significant reduction in AURKB levels following C28 treatment (Fig. S13A,B). This reduction was further corroborated by Western blotting analysis in C4-2 adherent cells (Fig. S13C). Additionally, immunofluorescence staining of C4-2 xenografted tumors revealed that both C28 and DHT (SAL) significantly reduced AURKB expression *in vivo* (Fig. 4F,G). Collectively, these data propose the novel finding that both AR-agonist and −antagonists can activate DREAM complex signaling. As control, in PC3 cells the expression of *ATAD2*, *AURKB* and *CCNA2* were also analyzed indicating no significant regulation (Fig. S11B), implying that the AR signaling controls DREAM complex activation.

### C28 and SAL induce growth repression and cellular senescence through DYRK1A-DREAM complex signaling

**Fig. 5:**
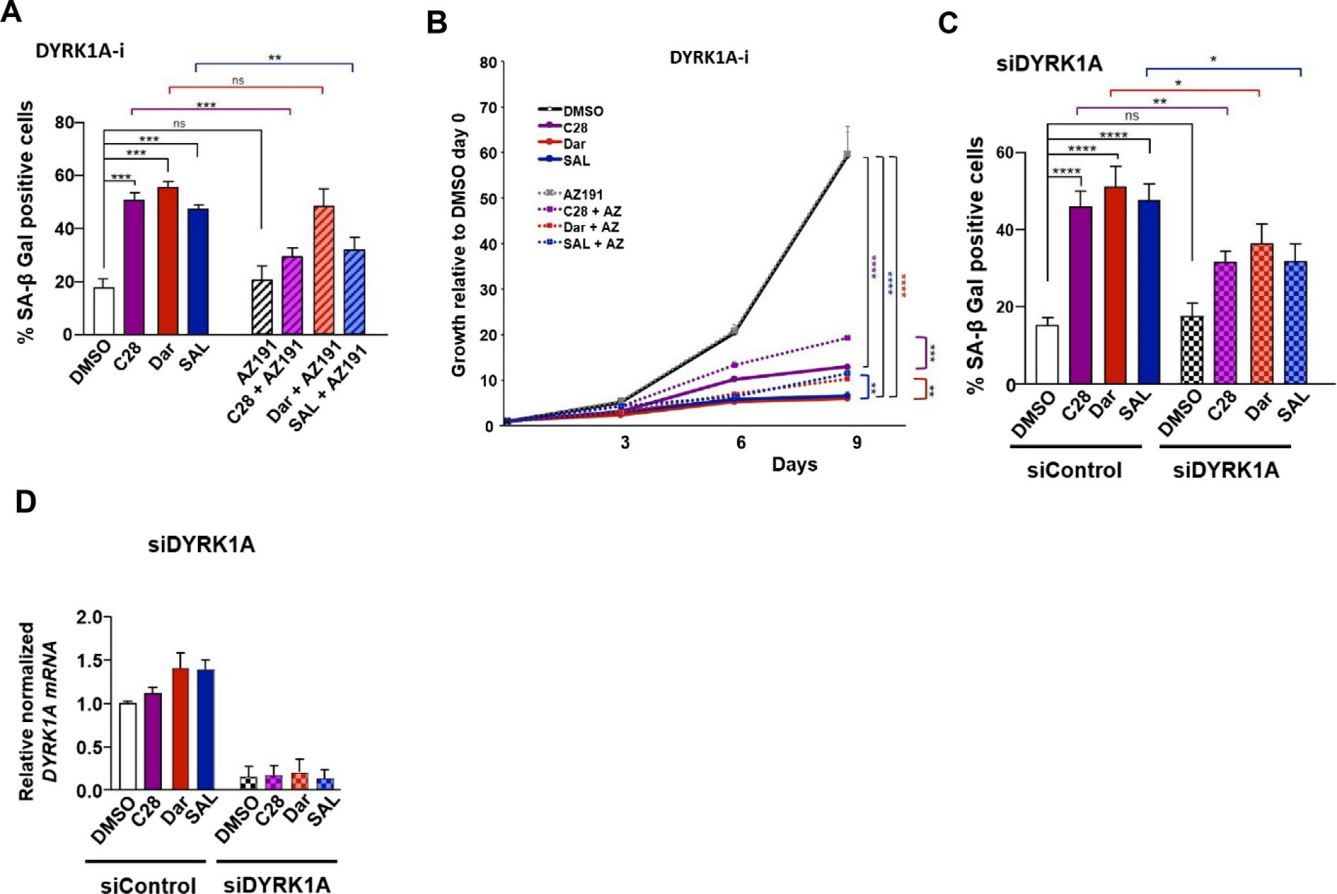

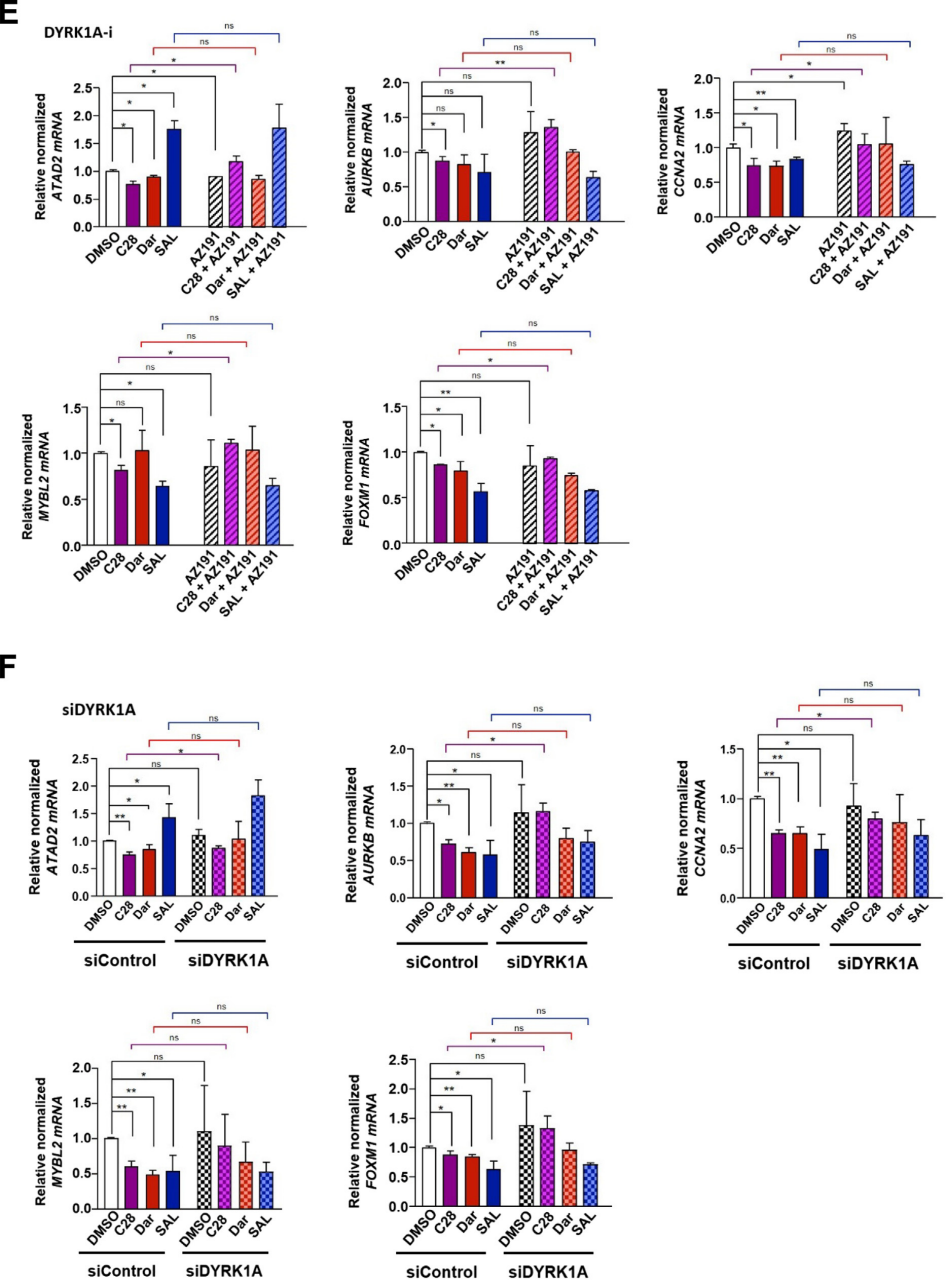
DYRK1A inhibition counteracts both cellular senescence induced by C28 or SAL and C28-mediated repression of DREAM target genes. **A:** Percentage of SA β-Gal positive cells in C4-2 treated cells with AZ191 (800 nM), a DYRK1A inhibitor (DYRK1A-i; AZ191), either alone or in combination with AR-ligands. Bar graphs are shown as mean ± SEM from twelve technical replicates (n = 12) of three independent experiments. **B:** Growth assay analysis over 9 days in C4-2 treated cells with or without DYRK1A-i (AZ) using crystal violet staining from four technical replicates (n = 4) of two independent experiments. **C:** Percentage of SA β-Gal positive cells in C4-2 cells transfected with siDYRK1A following AR-ligand treatments from twelve technical replicates (n = 12) of three independent experiments. **D:** Efficiency of DYRK1A knockdown (siDYRK1A). **E:** qRT-PCR to analyze the expression of DREAM complex target genes (*ATAD2*, *AURKB*, *CCNA2*, *MYBL2* and *FOXM1*) in C4-2 cells treated with the DYRK1A inhibitor AZ191 (DYRK1A-i, 800 nM) either alone or in combination with C28, Dar, SAL or DMSO, relative to DMSO. Bar graphs are shown as mean ± SEM from six technical replicates (n = 6) of two independent experiments. **F:** qRT-PCR analysis of DREAM complex target gene expression in C4-2 cells transfected with si-mediated knockdown of DYRK1A (siDYRK1A) following treating with C28, Dar, SAL or DMSO, relative to siControl DMSO from nine technical replicates (n = 9) of three independent experiments. *TBP* and *alpha-Tubulin* were used as housekeeping genes for normalization. Two-way ANOVA was used for all analysis for growth curves analyses, and a two-tailed unpaired Student *t*-test was used to compare each treatment with the control. (For interpretation of the references to color in this figure legend, the reader is referred to the web version of this article.)

Notably, the assembly of DREAM complex is dependent on the key kinase DYRK1A. It phosphorylates MuvB complex allowing p130 to bind and form the active DREAM complex [60,61]. Therefore, the role of DYRK1A for the induction of cellular senescence upon C28 and other AR ligands was examined. To assess this, both the DYRK1A inhibitor AZ191 or the knockdown of DYRK1A by si–RNA were employed to analyze the level of cellular senescence and growth. Both the AR-agonist and −antagonists induce cellular senescence (Fig. 5A and Fig. S14A) and inhibit growth (Fig. 5B and Fig. S14B) as expected. Single treatment with AZ191 (DYRK1A-i) alone did neither show a measurable effect on the level of cellular senescence nor for the growth rate (Fig. 5A, B and Fig. S14A, B). However, co-treatment of AZ191 with C28 or AR-agonist decreased the percentage of senescent cells compared to each ligand alone (Fig. 5A and Fig. S14A) suggesting that DYRK1A and the DREAM complex are part of AR signaling to mediate cellular senescence. To confirm that the observed effects are not solely due to potential off-target effects of the inhibitor, siRNA-mediated knockdown of DYRK1A was employed. While overexpression studies could serve as an alternative validation method, in some cases DYRK1A overexpression can lead to negative effects due to its kinase activity [62]. Excessive DYRK1A expression may cause excessive cell cycle arrest, limiting the efficacy of therapies targeting proliferative cancer cells [62]. Therefore, we opted for siRNA-mediated knockdown to provide a more specific assessment of DYRK1A's role in regulation of cellular senescence. Accordingly, the DYRK1A knockdown (siDYRK1A) alone did not induce cellular senescence in control treated cells (Fig. 5C and Fig. S14C). The efficiency of knockdown is shown in Fig. 5D. However, in combination with C28 and SAL, the siRNA-mediated DYRK1A knockdown reduces C28- and SAL-induced cellular senescence, confirming the data obtained with the DYRK1A inhibitor (Fig. 5C and Fig.S14C). Interestingly, while AZ191 co-treatment with Dar did not decrease significantly the percentage of senescent cells compared to Dar alone (Fig. 5A and Fig. S14A), knockdown of DRYK1A reduced Dar-induced senescence (Fig. 5C and Fig.S14C). Also, we noted that, co-treatment of AZ191 with Dar rather promoted cell growth compared to Dar alone (Fig. 5B and Fig. S14B), suggesting that Dar has additional targets to induce senescence and may suggest that not all AR-antagonists act only through DYRK1A. These findings indicate that both SAL and C28 use the DYRK1A-DREAM signaling to induce cellular senescence as a common pathway.

Since DYRK1A is an activator kinase of the DREAM complex [61], we analyzed the mRNA levels of DREAM complex target genes with treatment of the DYRK1A inhibitor AZ191 or knockdown by siDYRK1A transfected C4-2 cells in rescue experiments. Since C28 and SAL treatment activate the DREAM complex we hypothesized an increase of target gene expression in combination with DYRK1A inactivation. As expected, both C28 and SAL downregulated the mRNA expression of the analyzed DREAM target genes. The inhibition of DYRK1A enhanced mRNA levels of DREAM complex targets when C28 was applied (Fig. 5E, F) but interestingly not through SAL. This is accordance with the findings that SAL preferentially inhibits cell cycle at G1/S phase to induce cellular senescence whereas C28 inhibits G1/S/G2 phase and regulates DREAM complex those target genes that inhibit G1/S/G2 [54]. These findings suggest that C28, Dar and SAL induce cellular senescence through the DREAM complex whereas C28 inhibits in addition DREAM target genes that repress also S/G2.

### C28 induces cellular senescence in part through upregulated CCNG2 expression

**Fig. 6:**
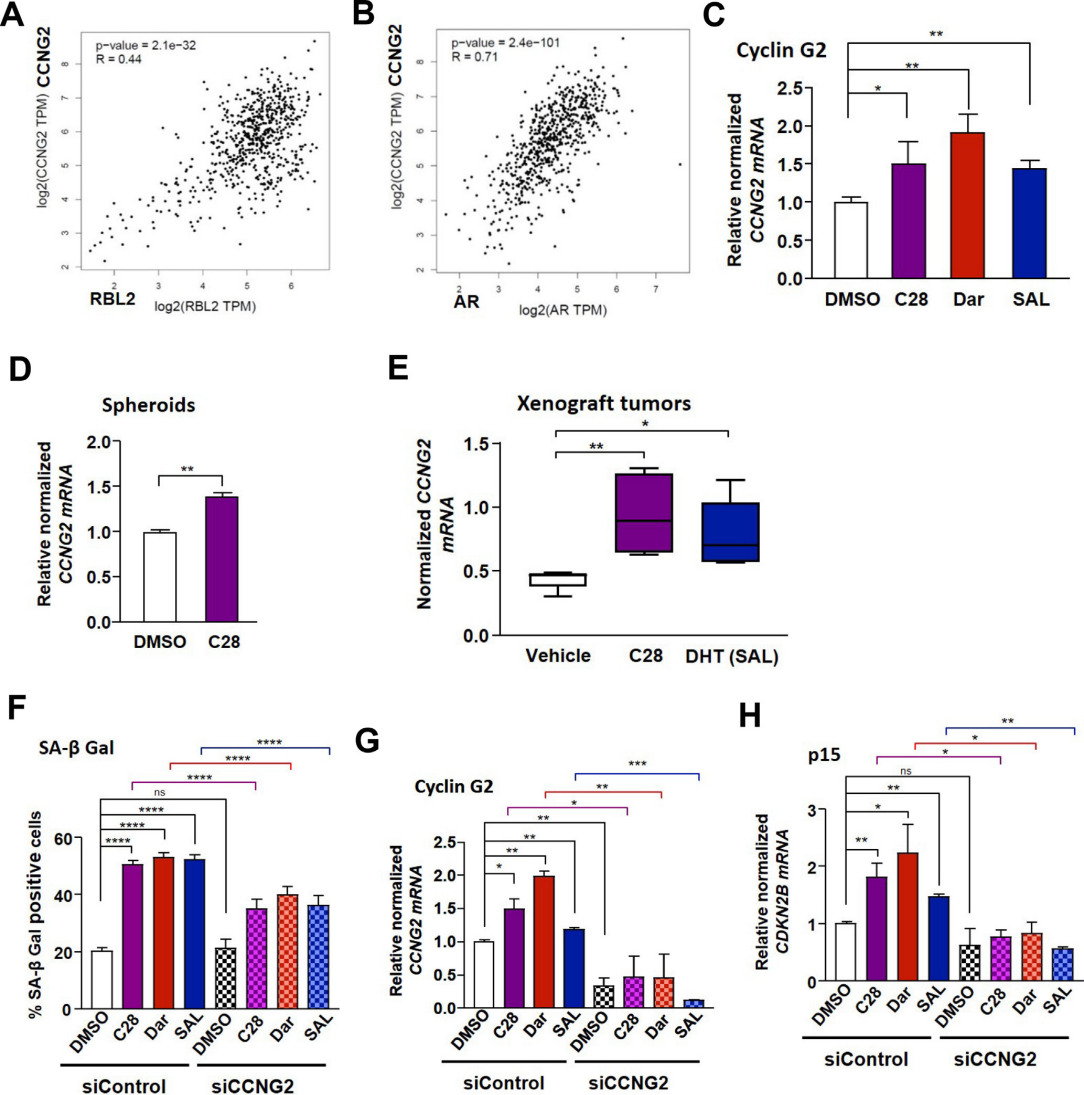
*CCNG2* is co-expressed with *AR* and *RBL2* and mediates cellular senescence induced by C28 or SAL**. A:** Correlation analysis between *RBL2* and *CCNG2* gene expression in PCa using GEPIA dataset. Values represent spearmen correlation coefficient, *p* < 0.05. **B:** Correlation analysis between *AR* and *CCNG2* gene expression in patients with PCa using GEPIA dataset. Values represent spearmen correlation coefficient. p < 0.05. **C:** qRT-PCR examination of *CCNG2* mRNA levels in treated C4-2 cells with C28, Dar, SAL or DMSO. Bar graphs are shown as mean ± SEM from nine technical replicates (n = 9) of three independent experiments. **D:** qRT-PCR analysis of *CCNG2* mRNA levels in C28 treated C4-2 spheroids from six technical replicates (n = 6) of two independent experiments. **E:** qRT-PCR analysis of *CCNG2* mRNA levels in C4-2 xenograft tumors of mice treated with C28 (N = 6), DHT (SAL, N = 7), or vehicle control (N = 5). **F:** Percentage of SA β-Gal positive cells in transfected C4-2 cells with siCCNG2 following AR-ligand treatments from eight technical replicates (n = 8) of two independent experiments. **G:** Efficiency of *CCNG2* knockdown. Bar graphs are shown as mean ± SEM from six technical replicates (n = 6) of three independent experiments. **H:** qRT-PCR analysis of *CDKN2B* mRNA levels in transfected C4-2 cells with siCCNG2 following AR-ligand treatments relative to siControl DMSO from six technical replicates (n = 6) of two independent experiments. For all qRT-PCR analyses, *TBP* and *alpha-Tubulin* were used as housekeeping genes for normalization. A two-tailed unpaired Student *t*-test was performed for statistical analysis.

Cyclin G2 is an unusual and a negative regulator of the cell cycle [63]. Interestingly, there are significant positive correlations in PCa between the expression levels of *RBL2* and that of *CCNG2* (Fig. 6A) as well as between *AR* and *CCNG2* (Fig. 6B) indicating that higher *RBL2* or *AR* expression could be associated with higher *CCNG2* expression. Survival association data of PCa patients suggests that low expression of *CCNG2* is associated with reduced survival of PCa patients (Fig. S15A), which is in line with its tumor suppressive activity.

In our previous study we observed that SAL enhances *CCNG2* mRNA levels [24], leading us to hypothesize that *CCNG2* expression is also regulated by AR-ligands and associated with AR-ligand induced cellular senescence. Interestingly, our data indicate that both AR-antagonists and AR-agonist elevate *CCNG2* mRNA levels in both C4-2 (Fig. 6C) and PC3-AR cell lines (Fig. S15B) suggesting a common pathway for AR-agonist at SAL and AR-antagonists with both types of ligand inhibit PCa tumor growth. Increased *CCNG2* mRNA levels were also detected in C28 treated C4–2 spheroids (Fig. 6D) as well as in xenograft CRPC tumors of mice treated with C28 (Fig. 6E). We also observed upregulation of *CCNG2* expression levels *in vivo* by SAL (Fig. 6E) consistent with our previous findings [24]. Importantly, no significant changes in *CCNG2* expression were observed in AR-negative PC3 cells upon C28 and SAL treatments (Fig. S15C) providing evidence of AR dependent regulation of this gene. Additionally, mRNA levels of *CCNG2* did not changed in 22Rv1 cells upon C28 or SAL treatment (Fig. S15D) and also in LNCaP Abl EnzaR spheroids in the presence of C28 (Fig. S15E), supporting the lack of growth inhibitory effects of C28 and SAL in these CRPC cell lines.

To address the question whether CCNG2 is part of AR signaling and mediates AR ligand-induced cellular senescence, siRNA-mediated knockdown targeting *CCNG2* was employed. The *CCNG2* knockdown itself did not significantly change the percentage of senescent cells (Fig. 6F, G and Fig. S15F) suggesting that the basal level or reduced basal level of CCNG2 is not part of senescence program in PCa cells. However, a significant reduction in cellular senescence using siCCNG2 following different AR-ligand treatments was observed (Fig. 6F and Fig. S15F). In line with this, enhanced growth was observed by the knockdown (Fig. S15G, H). Accordingly, p15^INK4b^ mRNA levels were downregulated after targeting *CCNG2* by siRNA in the presence of either AR-agonist or −antagonists (Fig. 6H). Of note, inhibition of DYRK1A did not modulate CCNG2 expression (Fig. S15I) suggesting that the induction of CCNG2 by C28 and SAL is independent of the DREAM complex pathway in order to induce cellular senescence. To further investigate whether the DYRK1A-DREAM and CCNG2 pathways act independently, the expression levels of DREAM targets following *CCNG2* knockdown were analyzed. Data show that knockdown of CCNG2 following treatment with C28 and SAL did not alter the expression of these genes (Fig. S15J) indicating that CCNG2 does not regulate DREAM complex targets. This further supports the notion that the induction of *CCNG2* by C28 and SAL occurs independently of the DREAM complex pathway in mediating cellular senescence. These findings suggest on one hand the identification of a novel DREAM-independent AR signaling pathway in CRPC cells mediated by CCNG2 and on the other hand that C28, Dar and androgen at supraphysiological levels induce cellular senescence through the same tumor suppressive CCNG2 pathway.

### Docking modeling suggests that C28 interacts with valine, glutamine and arginine residues of AR

**Fig. 7:**
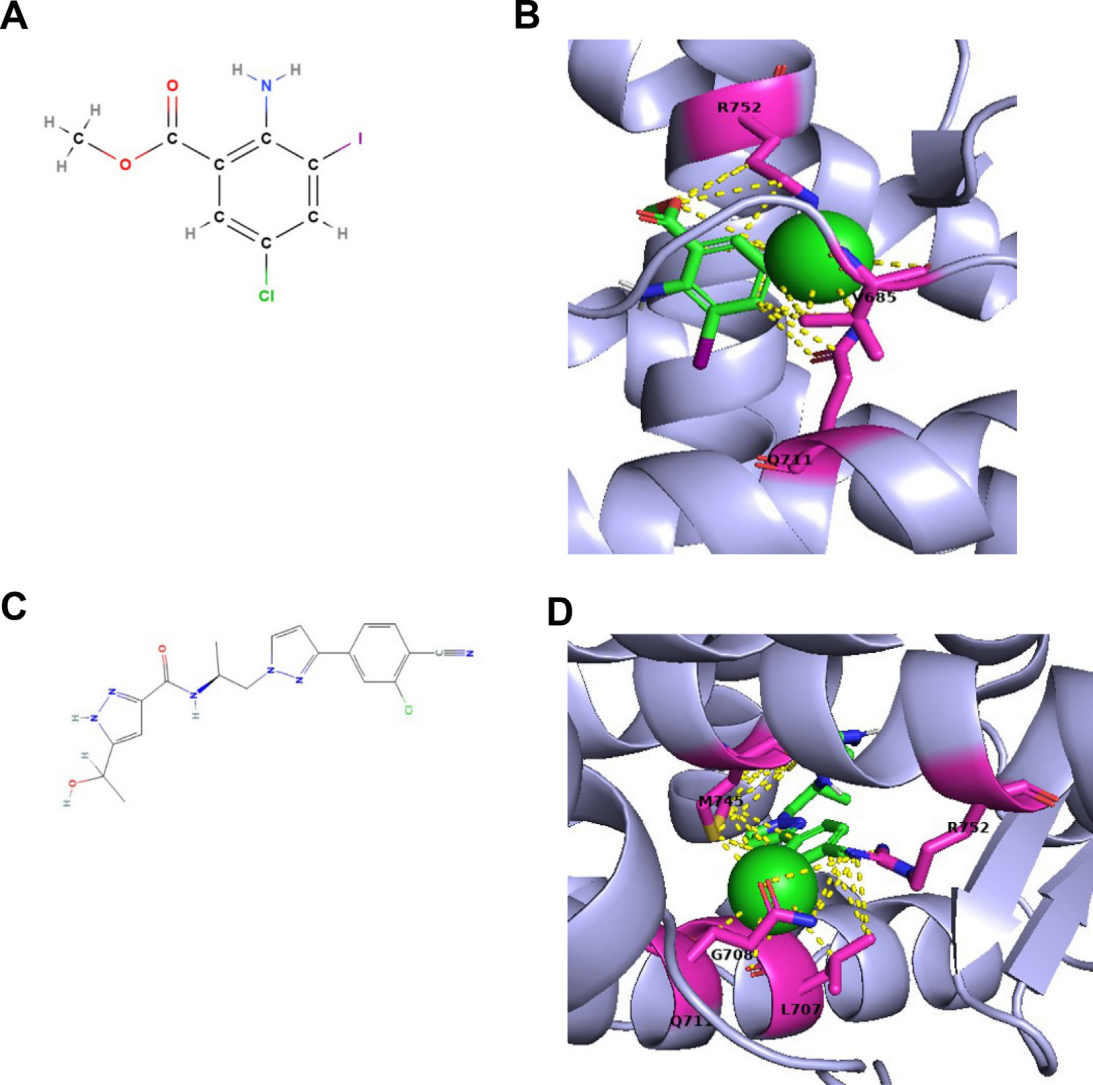
C28 interacts with valine, glutamine and arginine residues of AR being distinct from Dar. **A:** 2D structure of C28. **B:** Docking of C28 with AR, calculated affinity: −5.552 kcal/mol. **C:** 2D structure of Dar. **D:** Docking of Dar with AR, calculated affinity: −5.949 kcal/mol.

Previously, we showed that C28 binds to AR using competitive whole cell binding assays with radiolabeled androgen. Moreover, using AR deletion mutants, it suggests that C28 binds to and acts through the LBD of AR [11]. In contrast to Dar and androgens, C28 is a small molecule with only one benzene ring. To investigate potential interactions between AR residues and either C28 or Dar, ligand–protein docking modeling was performed. The predicted interaction between C28 (Fig. 7A) and AR with the highest binding affinity and lowest free energy (Fig. 7B) shows interaction of C28 with V685, Q711 and R752. Additionally, our docking results suggest that Dar (Fig. 7C) interacts with five AR residues (Fig. 7D), with only Q711 and R752 being common interaction residues for both C28 and Dar. Interestingly, R752 is a common residue in docking AR with other AR-antagonists, including Enz and AA [64,65] and may suggest a ligand specificity for V685 used as antagonism by C28. This prediction suggests that these three amino acids, though other amino acids may also be involved, mediate C28 interaction with AR. A different mode of interaction of C28 with AR compared to Dar and Enz is therefore suggested and might explain C28-mediated antagonism for those AR mutants that mediate therapy resistance.

## Discussion

Mutations in the LBD of AR are selected within the tumor evolution under pressure by AR-antagonists, leading to treatment resistance and development to CRPC [66], an aggressive form of PCa [30]. C28 inhibits both the wild-type AR and AR mutants resistant to clinically used AR-antagonists [11], indicating a different mode of interaction with AR and is suggested as a new class of AR-antagonists [67]. The chemical structure of C28 is a small molecule compared to Enz or Dar. Our docking results suggest that C28 interacts with the two AR residues R752 and Q711 also used by Enz or Dar AR-antagonists [31,64]. However, C28 also interacts with V685 not predicted by other AR-antagonists. Therefore, developing various types and chemical platforms of new AR-antagonists could be beneficial in overcoming therapy resistance that might arise from continuous treatment with only one AR-antagonist.

Mechanistically, C28 inhibits nuclear translocation of endogenous AR likely by inhibiting phosphorylation of both AR at Ser81 and HSP27, both being important for nuclear import of AR [43,45]. Notably, androgen-bound AR interacts with HSP27, leading to its phosphorylation and activation [68], thereby enhancing AR nuclear import and transcriptional activity [44,45]. Our results indicate that C28 reduces phosphorylation levels of HSP27 without necessarily disrupting the AR-HSP27 interaction in the cytosol. This suggests that C28 may alter the AR-HSP27 complex conformation, preventing HSP27 phosphorylation and indicates a novel pathway by which C28 inhibits AR nuclear translocation.

Here, the data suggest that C28 inhibits CRPC tumor growth both *in vitro* and *in vivo*. Notably, in most mouse tissues analyzed, both C28 and SAL did not change the expression of AR target genes suggesting minor side effects of AR-signaling pathways. Moreover, computational analysis predicted that C28 possesses favorable pharmacokinetic properties. These analyses also indicate that C28 can cross the blood–brain-barrier suggesting a potential for neurotoxic effects. While our *in vivo* studies did not reveal significant side effects, the possibility of central nervous system activity necessitates further investigation. SAL has progressed to clinical trials with some reported side effects including fatigue, nausea, and hematologic abnormalities [14,69]. These adverse events highlight the necessity for careful monitoring and management in clinical settings.

When comparing the growth inhibition effects of C28 with Dar, a potent second-generation AR-antagonist, the results are similar in extend. Similarly, treating xenograft mice the treatment with 100 mg/kg Dar reduces the castration-resistant VCaP xenograft tumor growth by 20 % [50] and 100 mg/kg C28 reduces tumor growth by approximately 50 % (Fig. 2H). Of note, both androgen at supraphysiological levels and various AR-antagonists induce cellular senescence in CRPC [13,70]. This clearly emphasizes that AR-antagonists not only inactivate the AR, rendering it non-functional, but induce cellular senescence in PCa suggesting that part of AR signaling is induced by antagonists. We hypothesize that C28 inhibits AR translocation and inhibits growth of CRPC cells *in vitro* and *in vivo* through stabilizing p130 non-genomically, thereby increases p130 protein level and induces cellular senescence.

Hypophosphorylation of the retinoblastoma protein by SAL treatment is associated with cellular senescence in castration-sensitive PCa being in line with enhancing CDK inhibitors such as p15^INK4b^ [48]. Similar findings are obtained for CRPC by SAL. Since C28 does not strongly hypophosphorylate pRb compared to SAL, it implies the involvement of alternative pathways. Previous studies by our group showed that SAL induces cellular senescence through enhanced AKT phosphorylation and downstream signaling. In contrast, C28 does not enhance AKT phosphorylation (data not shown), suggesting an alternative non-AKT pathway is used by C28. This also suggests that SAL or C28 use not only a common pathway through p130 but also a different signaling to induce cellular senescence. This may also suggest that there are different signaling pathways to induce cellular senescence regulated by distinct AR ligands and one common pathway of AR-agonist and −antagonist flows into p130 signaling in PCa.

The DREAM complex with p130 as a complexed factor is a tumor suppressor acting protein complex that controls numerous cell cycle genes and contributes to cell cycle arrest [71].

Therefore, we suggest that as an alternative pathway, p130 is induced by C28 and also by SAL in an AR-dependent manner. Interestingly, we identified an interaction between AR and p130 at protein levels upon C28 or SAL treatments. This suggests that p130 might have a common role in the growth inhibition and induction of cellular senescence by these treatments. ChIP-seq data suggest direct binding of AR to the *RBL2* gene, indicating that p130 is a direct AR target gene and may explain the induction of p130 by SAL.

Notably, p130 can act as a transcriptional activator binding to E2F1 [53]. ChIP-seq data analyses suggest that p130 is recruited to its own promoter and interestingly is overlapping with ChIP of E2F1 at the same genomic locus. This indicates that p130 through recruitment to E2F1 site may upregulate its own expression. Thus, the interaction of p130 with AR at protein level in the presence of an AR ligand, SAL or C28, may stabilize p130 protein level that subsequently enhances its own expression. A key finding is that this mechanism to upregulate p130 seems to be a common pathway between AR-antagonist and AR-agonist. Thus, mechanistically, we suggest that AR bound to C28 or SAL stabilizes p130.

Our findings suggest that the activation of DREAM and downregulation of its target genes in various CRPC models is consistent with the DREAM complex role as a negative regulator of cell cycle progression and patient survival [54]. ChIP-seq data reveals the recruitment of p130 to important cell cycle-related genes, including *ATAD2*, *AURKB*, *CCNA2*, *MYBL2* and *FOXM1*. Since we found that these genes are regulated by treatment with AR-ligands, it reinforces the notion that AR-ligands control the DREAM signaling pathway. The convergence of both AR-antagonists and −agonist on this pathway suggests a complex regulation of AR signaling in PCa, where both activation and inhibition of the receptor can lead to similar downstream effects to control cell cycle and induce cellular senescence.

DYRK1A is the upstream factor and key kinase of DREAM complex pathway and controls the assembly of this complex [54]. Using DYRK1A inhibitor (DYRK1A-i) or knockdown (siDYRK1A) suggests that C28 through AR induces cellular senescence by the DYRK1A-DREAM pathway. These results align with studies indicating that inhibiting DYRK1A disrupts DREAM complex assembly and reduces the percentage of senescent cells in oncogene-induced human fibroblasts [60]. In glioblastoma, which demonstrates significant AR expression and activation, the DYRK1A kinase plays a crucial role in regulating cell cycle and differentiation. The DYRK1A-mediated assembly of DREAM complex promotes cellular quiescence and may act as a tumor suppressor [72].

Although G1/S arrest is the most commonly observed phase of senescence-associated cell cycle arrest in PCa [73], in general, senescence can also occur at other cell cycle phases [74]. For human fibroblasts a G2 arrest-associated senescence was reported in response to DNA damage [74]. Some chemotherapeutic agents induce cell cycle arrest at the S phase by interfering with DNA replication [75]. Here, our data suggest that C28 induces cellular senescence G1/S/G2 cell cycle supporting the notion that the DREAM complex is involved in C28-induced cellular senescence. Our data further suggest that treatment with Dar likely does not induce senescence through DREAM protein complex. Therefore, it is suggested that Dar induces senescence through alternative pathways. Reduction or inhibition of DYRK1A decreased SAL-mediated senescence but did not change the expression of analyzed G2/M DREAM target genes, suggesting that SAL may induce cellular senescence through G1/S targets of the DYRK1A-DREAM pathway. This aligns with a recent report that DREAM complex can mediate growth repression under SAL treatment during the G1/S phase [54].

It is noteworthy, that the inhibition of DYRK1A did not change the *CCNG2* expression and knockdown of CCNG2 did not affect the mRNA levels of DREAM complex-regulated targets suggesting that C28 or SAL induce two independent pathways to mediate cellular senescence. The expression of *CCNG2* encoding an atypical cyclin that inhibits cell cycle progression, is decreased in PCa [63]. ChIP-seq results suggest that p130 and E2F1 are recruited to the promoter region of *CCNG2* (Fig. S15K). We propose that the recruitment of p130 to the regulatory region of *CCNG2* may lead to its upregulation. Of note, *CCNG2* mRNA level is regulated by various miRNAs to control its stability. This includes miR-590-3p, miR-1246, miR-340 [76], miR-1290 [77] that have been identified as negative regulators of *CCNG2* mRNA stability. C28 may upregulate *CCNG2* expression either through AR by stabilizing p130, thereby facilitating its recruitment to *CCNG2* gene loci or by influencing the expression of aforementioned miRNAs. SAL may upregulate *CCNG2* gene expression through non-genomic by stabilizing p130 through p130-AR protein complex formation or at genomic level via recruitment of AR to the *CCNG2* gene locus [24].

Reduction in cellular senescence and p15^INK4b^ mRNA levels following CCNG2 knockdown in cells treated with C28, Dar or SAL suggest that CCNG2 is a common key mediator of senescence response by AR-ligands using either AR-antagonists or androgens at supraphysiological concentrations. Accordingly, the growth promotion observed after *CCNG2* knockdown in C28- or SAL-treated cells supports the notion that cyclin G2 inhibits cell cycle and mediates cellular senescence in CRPC cells. Cyclin G2 induced by C28 and SAL has not been identified as a DREAM complex target [78]. The latter is known to rather repress its target genes. This implies that induction of cyclin G2 by both SAL and C28 or Dar treatment represents another and DREAM-complex-independent molecular pathway. Both the DYRK1A-DREAM pathway and CCNG2 contribute to C28- and SAL-induced cellular senescence. Our findings indicate that these pathways function independently rather than synergistically. The data support the notion that C28 and SAL activate two parallel mechanisms to drive cellular senescence, rather than a single, interconnected pathway.

SAL and various AR-antagonists induce cellular senescence in PCa cells, despite supposedly acting in an opposite manner on AR signaling. The underlying mechanism by which these AR-ligands converge into a similar cellular pathway was puzzling. For SAL, the AR-AKT signaling was shown to partially mediate cellular senescence. The activation of AKT singling was, however, not observed with C28, indicating the involvement of other pathways that might be common between agonist and antagonist. Here, our data suggest that cyclin G2 and the pocket domain protein p130 could be these common factors. Thus, both AR-agonist and –antagonist enhance p130 and *CCNG2* levels as a common mechanism for growth inhibition. Notably, other cancers that express AR, such as triple-negative breast cancer and glioblastoma may also activate one or both pathways [72,79]. However, it is unclear whether these pathways induce cellular senescence in these or other AR-dependent cancer types.

Our data indicates that enzalutamide resistant prostate cancer spheroids exhibit cross-resistance to C28 treatment. This suggest that resistance mechanisms developed against one AR-antagonist may confer resistance to others. Specifically, the expression of constitutively active AR splice variant, AR-V7, has been implicated in resistance to treatment with different AR-antagonist-based therapies [80].

## Conclusion

**Fig. 8:**
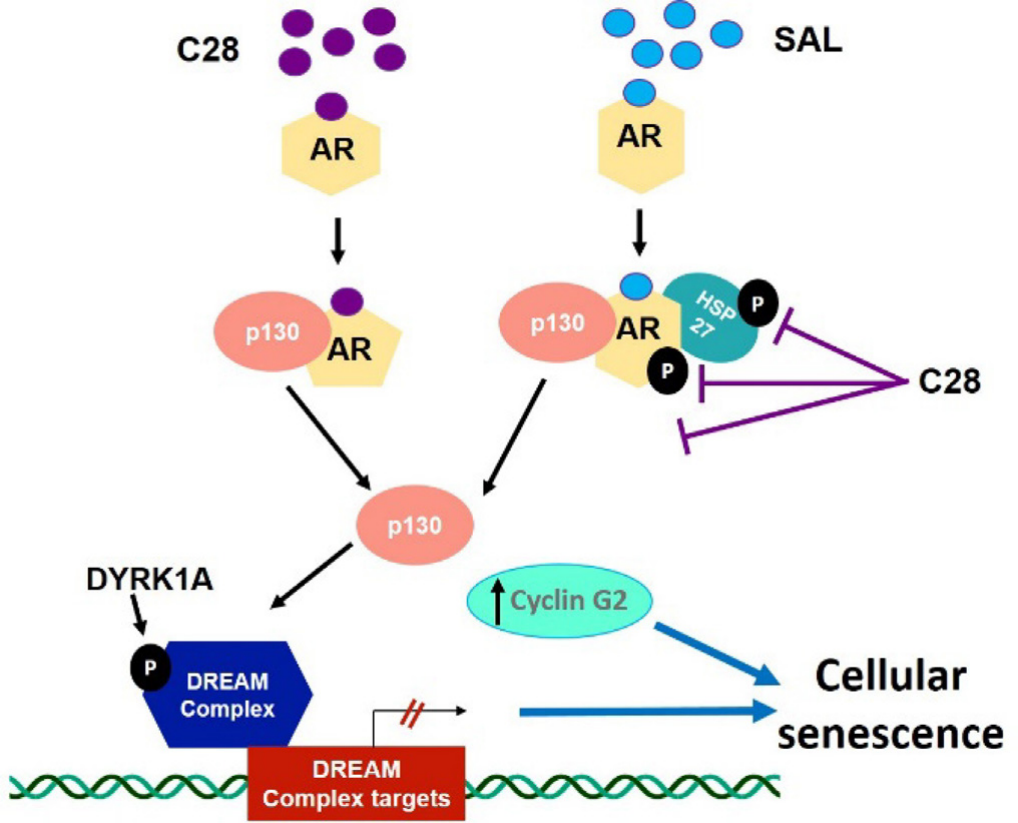
C28 induces cellular senescence via two pathways. On one hand by AR-p130-DYRK1A-DREAM complex and the other hand by upregulation of CCNG2 signaling. C28- or androgen-bound AR, at SAL, interacts with p130 leading to stabilization of p130 and promoting the formation of the p130-AR protein complex. DYRK1A activation leads to DREAM complex assembly to induce cellular senescence. In common, C28 and SAL also upregulate *CCNG2* expression further contributing to the induction of cellular senescence. The figure was in part generated using Biorender.

Taking together, our finding suggest the identification of two novel independent signaling pathways commonly used by the AR-antagonist C28 and SAL to induce cellular senescence: One pathway is through AR-p130-DYRK1A-DREAM signaling and the other by activating the atypical CCNG2 signaling (Fig. 8), which both arrest cells at G1/S/G2 and induce cell senescence.

## Compliance with ethics requirements

*Approval for in vivo mouse experiments was obtained from Thüringer Landesamt für Lebensmittelsicherheit und Verbraucherschutz, Germany (Reg.-Nr.: UKJ-23-013) carried out in accordance with The Code of Ethics of the World Medical Association (Declaration of Helsinki)*.

## CRediT authorship contribution statement

GAR performed experiments, validation and data collection, MHH and JK performed mouse experiments. MHH performed bioinformatic analyses, KS generated and analyzed spheroids. AB designed and supervised the study, and revised manuscript. All the authors read and approved the submitted version.

## Funding

This work was supported by the German Academic Exchange Service (DAAD; Deutscher Akademischer Austauschdienst) [to M.H.H (91795579).; G.A.R (91762223)], Jena University Hospital (to A.B.), and the Deutsche Krebshilfe (German Cancer Aid #70113814 to A.B).

## Declaration of competing interest

*The authors declare that they have no known competing financial interests or personal relationships that could have appeared to influence the work reported in this paper*.

## Data Availability

The datasets obtained and/or analyzed during the current study are available from the corresponding author on reasonable request. Our RNA-seq datasets from C4-2 cells and those from VCaP cells, C4-2 xenografts and PDX xenografts are accessible in Gene Expression Omnibus (GEO) under accession numbers GSE172205, GSE148397, GSE179687, and GSE188174, respectively. The ChIP-Seq datasets for p130, AR, and Rb were obtained from GEO under accession number GSE179684, along with E2F1 ChIP-seq datasets with accession number GSE154191. The GEPIA datasets were used for correlation analysis. Protein atlas database were used for Kaplan-Meier survival plots. Tumor, normal and metastasis PCa samples were retrieved from TNM web tool, updated in June 2023 to analyze expression in normal and patient PCa samples.
