# Supplementary material for: Induction of cellular senescence by androgen receptor agonist or antagonist is mediated via two novel common DYRK1A-DREAM and cyclin G2 signaling pathways in castration-resistant prostate cancer
Source: J Adv Res. 2025 May 12;80:371–92. doi: 10.1016/j.jare.2025.05.019 (PMC12869228; doi:10.1016/j.jare.2025.05.019)
Supplement: Supplementary Data 1 [file mmc1.docx]

| **Table S1. Antibodies** | | | |
| --- | --- | --- | --- |
| Antibody | Dilution | Company | Cat No |
| anti-AR | 1:1000 for WB  1:50 for Co-IP  1:600 for IF | Merck Millipore | #06-680 |
| anti-pAR (S81) | 1:1000 | Merck Millipore | #07-1375 |
| anti-HSP27 | 1:2500 for WB | Enzolifescience | SPA800D |
| anti-pHSP27 | 1:1000 | Epitomics | #1118-1 /032509 |
| anti-pRb | 1:1000 | Abcam | ab6075 |
| anti-phospho (S807/811)- pRb | 1:1000 | Cell Signaling | #1144 |
| anti-p130 | 1:1000 for WB | Santa Cruz | sc-317 |
| anti-p15^INK4b^ | 1:2000 | MyBiosource | MBS821044 |
| anti-p16^INK4a^ | 1:1000 | Cell Signaling | #80772S |
| anti-β-Actin | 1:10000 | Abcam | Ab6276 |
| anti-Ki67 | 1:200 | Abcam | AB243878 |
| anti-Aurora B | 1:1000 for WB  1:500 for IF | Cell Signaling | #28711 |
| anti-mouse IgG HRP | 1:10000 | Cell Signaling | #7076S |
| anti-rabbit IgG HRP | 1:10000 | Cell Signaling | #7074S |
| anti-rabbit IgG Alexa 546 | 1:1000 | Invitrogen | A11035 |

| **Table S2. Sequence of used primers (5’ ... 3’)** | |
| --- | --- |
| *FKBP5* | Fw: GAGGAAACGCCGATGATTGGAGAC  Rev: CATGCCTTGATGACTTGGCCTTTG |
| *NKX3.1* | Fw: CCGAGACGCTGGCAGAGACC  Rev: GCTTAGGGGTTTGGGGAAG |
| *hTERT* | Fw: CGGAAGAGTGTCTGGAGCAA  Rev: GGATGAAGCGGAGTCTGGA |
| *CDKN2B* (p15) | Fw: GAATGCGCGAGGAGAACAAG  Rev: TCATCATGACCTGGATCGCG |
| *CDKN2A* (p16) | Fw: CTTGCCTGGAAAGATACCG  Rev: CCCTCCTCTTTCTTCCTCC |
| *RBL2* (p130) | Fw: ATTTGGCATGGAAACCAGAG  Rev: ATCTGCCCTTTCCAGGTTCT |
| *ATAD2* | Fw: GGAATCCCAAACCACTGGACA  Rev: GGTAGCGTCGTCGTAAAGCACA |
| *AURKB* | Fw: ACAGACGGCTCCATCTGGCCT  Rev: GGCAGCTGTGGGCTGGACATT |
| *CCNA2* (Cyclin A2) | Fw: GGTACTGAAGTCCGGGAACC  Rev: TGAACGCAGGCTGTTTACTG |
| *FOXM1* | Fw: ATACGTGGATTGAGGACCACT  Rev: TCCAATGTCAAGTAGCGGTTG |
| *MYBL2* | Fw: CACCAGAAACGAGCCTGCCTTA  Rev: CTCAGGTCACACCAAGCATCAG |
| *CCNG2* (Cyclin G2) | Fw: GTGTTCCTGAGCTGCCAACGAT  Rev: AGGTGCACTCTTGATCACTGGG |
| *TBP* | Fw: GATCTTTGCAGTGACCCAGCATCA  Rev: CTCCAGCACACTCTTCTCAGC |
| *TUBA* (α-Tubulin) | Fw: TGGAACCCACAGTCATTGATGA  Rev: TGATCTCCTTGCCAATGGTGTA |
| *Pbsn* | Fw: GGTCATCATCCTCCTGCTCA  Rev: AGCTAAGTAAATTGTTTGCCAAGG |
| *Sox9* | Fw: GGTTTCAGATGCAGTGAGGAGC  Rev: CACATACAGTCCAGGCAGACC |
| *Gus* | Fw: TGCCTGTCCCTTCTAGCTTC  Rev: TGTTCCACCACATGAATCCCATTC |
| *Hsd3b6* | Fw: GGACCAGCTGGGATACAGAA  Rev: ACAGTGACCCTGGAGATGGT |
| *Rhox5* | Fw: GGAGCAGGAACAAAATGAGC  Rev: TGGACTCCAGTTCCCTCAGT |
| *Gstp1* | Fw: ACCATACACCATTGTCTACTTCCC  Rev: GGTAAAGGGTGAGGTCTCCAT |
| *Rpl13a* | Fw: CTGAAGCCTACCAGAAAGTTTGC  Rev: CGTCCTGTTTTCCGTAACCTCAAG |

| **Table S3. Predicted pharmacokinetic and toxicity properties of C28** | | |
| --- | --- | --- |
| Classification | Prediction | Probability score |
| Gastrointestinal absorption | High |  |
| Skin permeability | Low | -6.13 cm/s ^#^ |
| Blood-brain barrier permeation | Yes | 0.79* |
| Cytochrome P450 1A2 (CYP1A2) inhibitor | No | 0.67* |
| Cytochrome P450 2C19 (CYP2C19) inhibitor | No | 0.75* |
| Cytochrome P450 2D6 (CYP2D6) inhibitor | No | 0.56* |
| Cytochrome P450 3A4 (CYP3A4) inhibitor | No | 0.85* |
| Cytochrome P450 2C9 (CYP2C9) inhibitor | Yes | 0.68* |
| Cardiotoxicity | No | 0.87* |
| Immunotoxicity | No | 0.97* |
| Cytotoxicity | No | 0.64* |
| Mutagenicity | No | 0.64* |

^#^ Log *K_p_* is a logarithmic value of the skin permeability coefficient (*K_p_*). Typical ranges of skin permeability are divided to three groups, Log *K_p_* > -3.0: High skin permeation, Log *K_p_* between -3.0 and -6.0: Moderate skin permeation, Log *K_p_* < -6.0: Low skin permeation.

^*^The range of probability is between 0 and 1, with 1 representing the highest probability.
