# Supplementary material for: Induction of cellular senescence by androgen receptor agonist or antagonist is mediated via two novel common DYRK1A-DREAM and cyclin G2 signaling pathways in castration-resistant prostate cancer
Source: J Adv Res. 2025 May 12;80:371–92. doi: 10.1016/j.jare.2025.05.019 (PMC12869228; doi:10.1016/j.jare.2025.05.019)
Supplement: Supplementary Data 2 [file mmc2.pptx]

## Slide 1
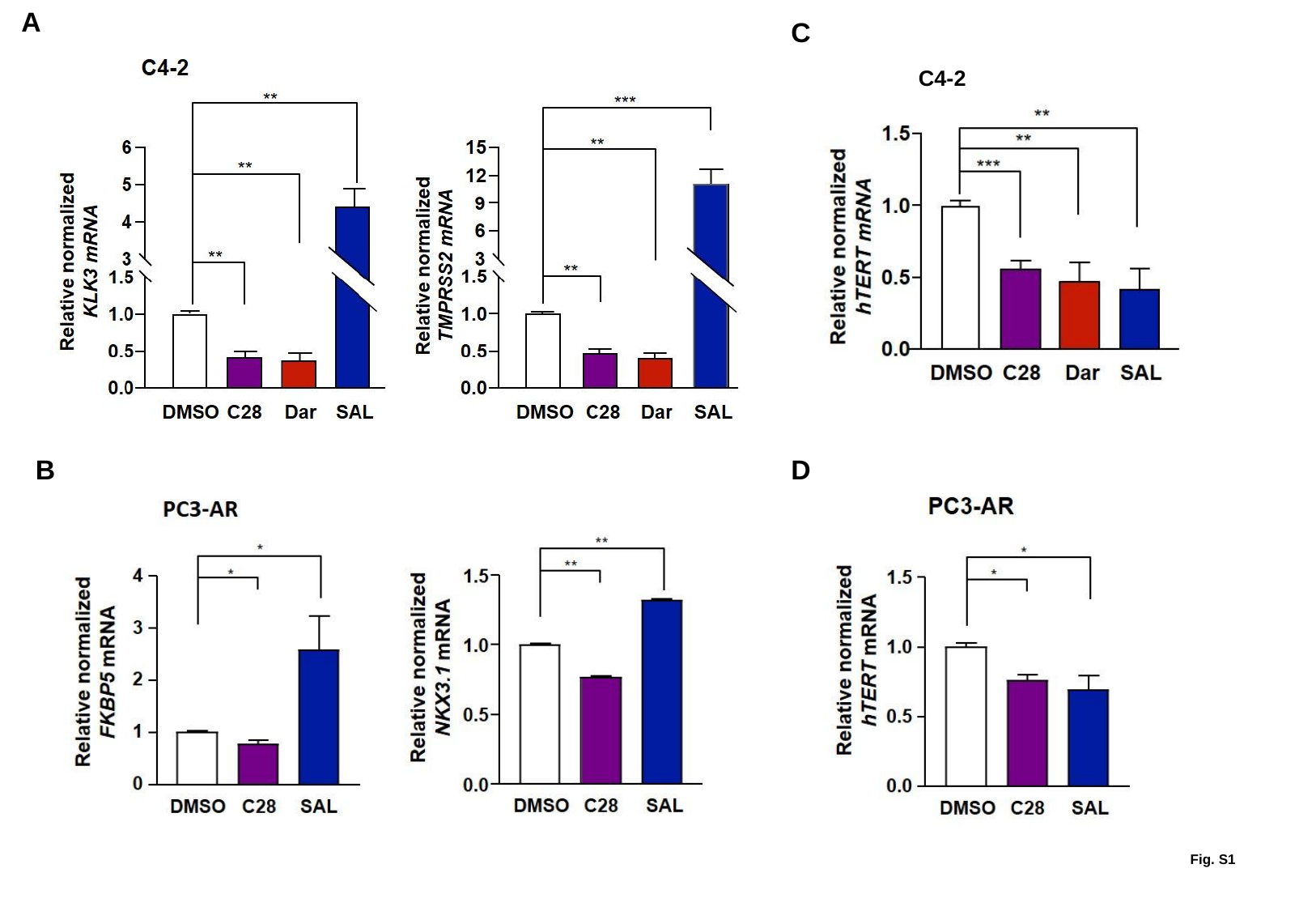

A
C
C4-2
D
B
Fig. S1

## Slide 2
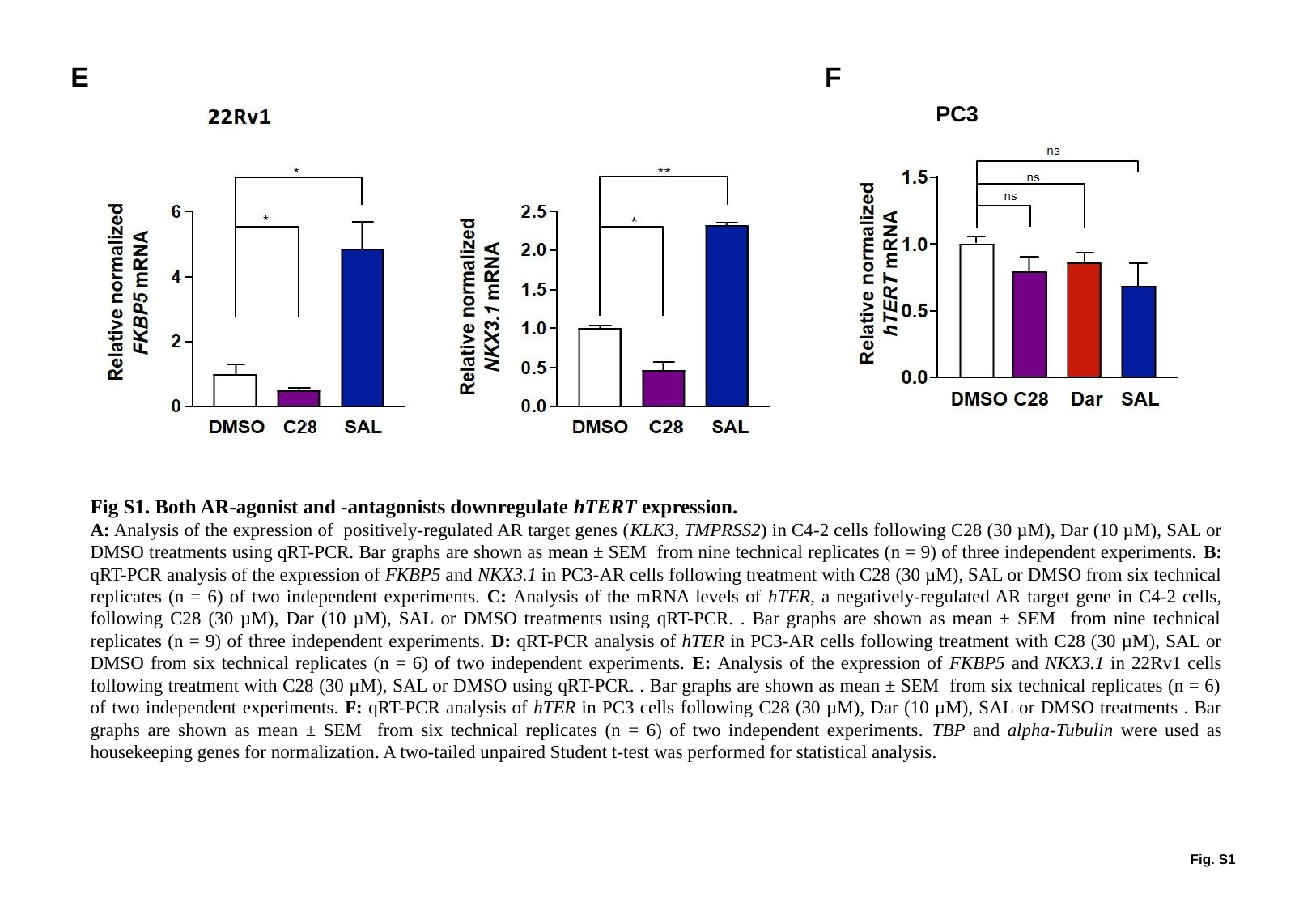

E
F
PC3
Fig S1. Both AR-agonist and -antagonists downregulate hTERT expression.
A: Analysis of the expression of positively-regulated AR target genes (KLK3, TMPRSS2) in C4-2 cells following C28 (30 µM), Dar (10 µM), SAL or DMSO treatments using qRT-PCR. Bar graphs are shown as mean ± SEM from nine technical replicates (n = 9) of three independent experiments. B: qRT-PCR analysis of the expression of FKBP5 and NKX3.1 in PC3-AR cells following treatment with C28 (30 µM), SAL or DMSO from six technical replicates (n = 6) of two independent experiments. C: Analysis of the mRNA levels of hTER, a negatively-regulated AR target gene in C4-2 cells, following C28 (30 µM), Dar (10 µM), SAL or DMSO treatments using qRT-PCR. . Bar graphs are shown as mean ± SEM from nine technical replicates (n = 9) of three independent experiments. D: qRT-PCR analysis of hTER in PC3-AR cells following treatment with C28 (30 µM), SAL or DMSO from six technical replicates (n = 6) of two independent experiments. E: Analysis of the expression of FKBP5 and NKX3.1 in 22Rv1 cells following treatment with C28 (30 µM), SAL or DMSO using qRT-PCR. . Bar graphs are shown as mean ± SEM from six technical replicates (n = 6) of two independent experiments. F: qRT-PCR analysis of hTER in PC3 cells following C28 (30 µM), Dar (10 µM), SAL or DMSO treatments . Bar graphs are shown as mean ± SEM from six technical replicates (n = 6) of two independent experiments. TBP and alpha-Tubulin were used as housekeeping genes for normalization. A two-tailed unpaired Student t-test was performed for statistical analysis.
Fig. S1

## Slide 3
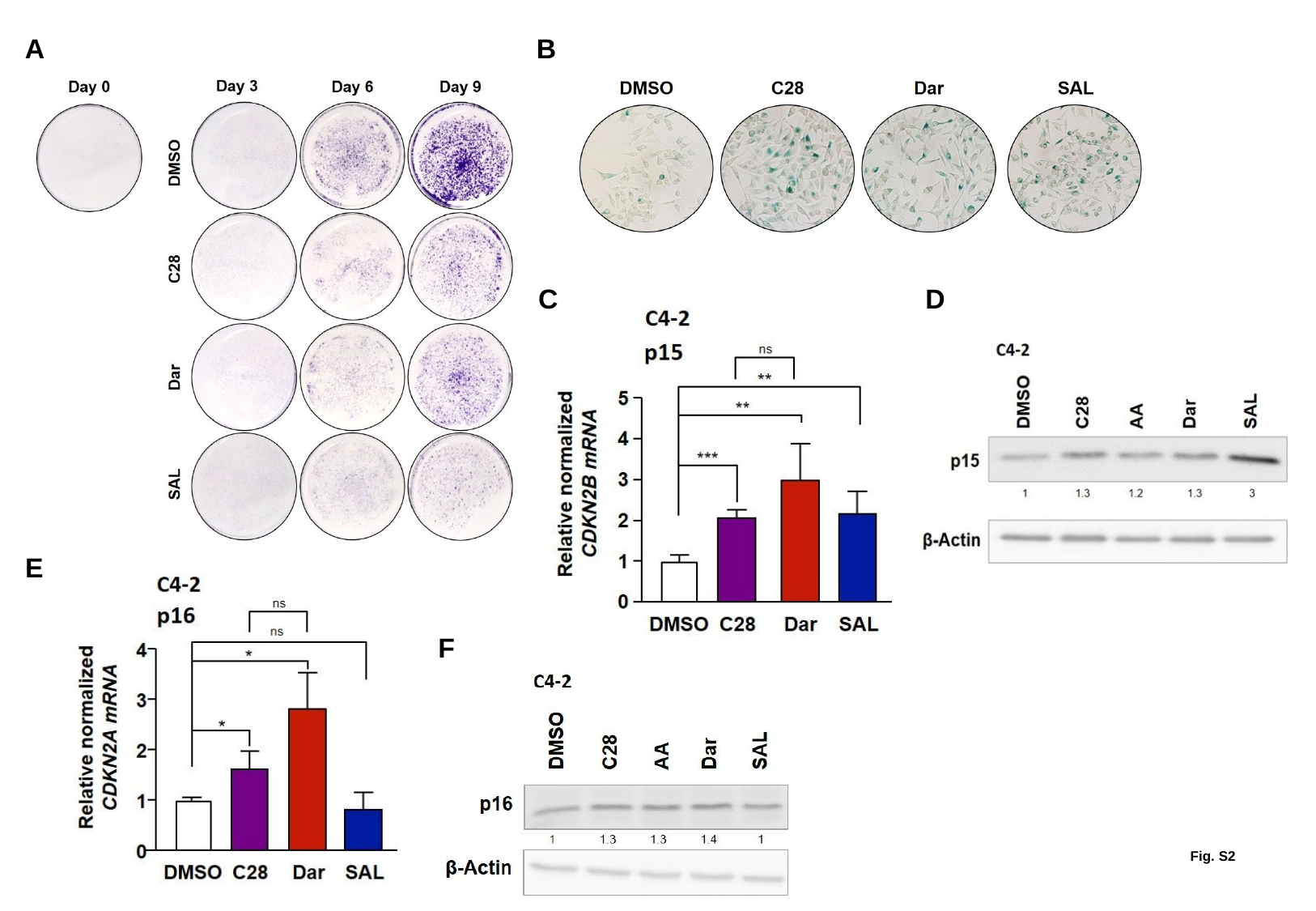

A
B
D
C
E
F
Fig. S2

## Slide 4
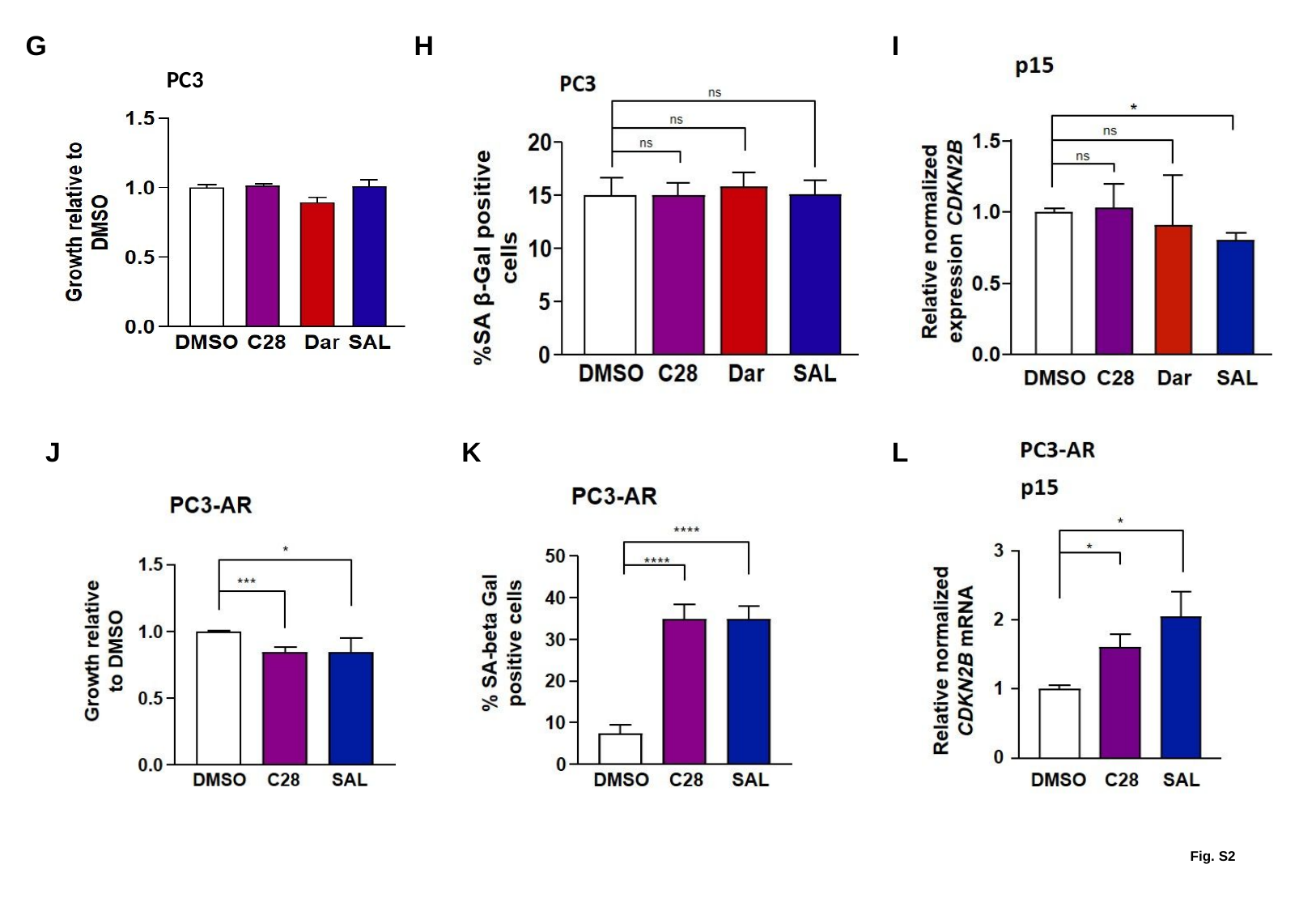

G
H
I
PC3
K
L
J
Fig. S2

## Slide 5
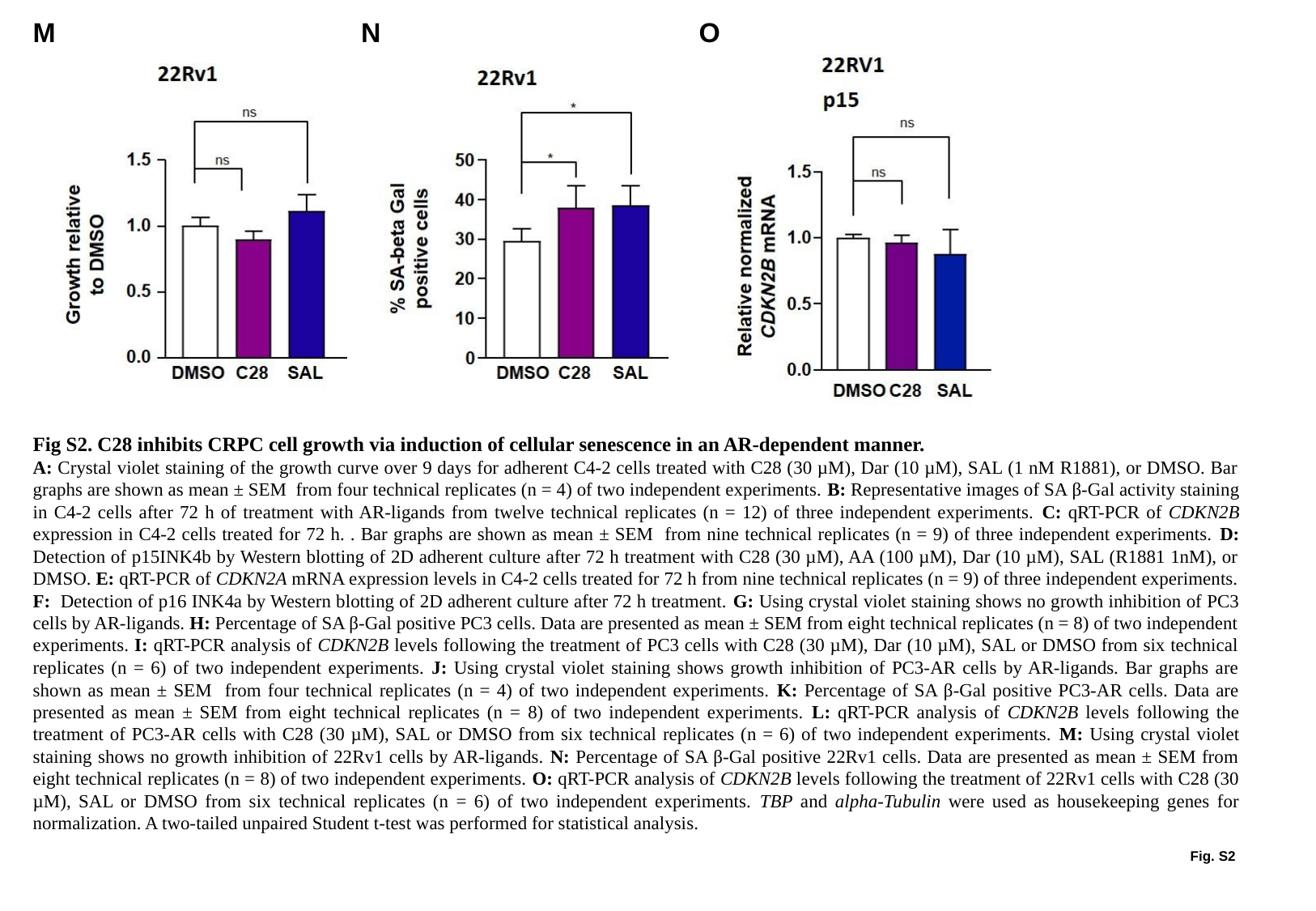

M
N
O
Fig S2. C28 inhibits CRPC cell growth via induction of cellular senescence in an AR-dependent manner.
A: Crystal violet staining of the growth curve over 9 days for adherent C4-2 cells treated with C28 (30 µM), Dar (10 µM), SAL (1 nM R1881), or DMSO. Bar graphs are shown as mean ± SEM from four technical replicates (n = 4) of two independent experiments. B: Representative images of SA β-Gal activity staining in C4-2 cells after 72 h of treatment with AR-ligands from twelve technical replicates (n = 12) of three independent experiments. C: qRT-PCR of CDKN2B expression in C4-2 cells treated for 72 h. . Bar graphs are shown as mean ± SEM from nine technical replicates (n = 9) of three independent experiments. D: Detection of p15INK4b by Western blotting of 2D adherent culture after 72 h treatment with C28 (30 µM), AA (100 µM), Dar (10 µM), SAL (R1881 1nM), or DMSO. E: qRT-PCR of CDKN2A mRNA expression levels in C4-2 cells treated for 72 h from nine technical replicates (n = 9) of three independent experiments. F: Detection of p16 INK4a by Western blotting of 2D adherent culture after 72 h treatment. G: Using crystal violet staining shows no growth inhibition of PC3 cells by AR-ligands. H: Percentage of SA β-Gal positive PC3 cells. Data are presented as mean ± SEM from eight technical replicates (n = 8) of two independent experiments. I: qRT-PCR analysis of CDKN2B levels following the treatment of PC3 cells with C28 (30 µM), Dar (10 µM), SAL or DMSO from six technical replicates (n = 6) of two independent experiments. J: Using crystal violet staining shows growth inhibition of PC3-AR cells by AR-ligands. Bar graphs are shown as mean ± SEM from four technical replicates (n = 4) of two independent experiments. K: Percentage of SA β-Gal positive PC3-AR cells. Data are presented as mean ± SEM from eight technical replicates (n = 8) of two independent experiments. L: qRT-PCR analysis of CDKN2B levels following the treatment of PC3-AR cells with C28 (30 µM), SAL or DMSO from six technical replicates (n = 6) of two independent experiments. M: Using crystal violet staining shows no growth inhibition of 22Rv1 cells by AR-ligands. N: Percentage of SA β-Gal positive 22Rv1 cells. Data are presented as mean ± SEM from eight technical replicates (n = 8) of two independent experiments. O: qRT-PCR analysis of CDKN2B levels following the treatment of 22Rv1 cells with C28 (30 µM), SAL or DMSO from six technical replicates (n = 6) of two independent experiments. TBP and alpha-Tubulin were used as housekeeping genes for normalization. A two-tailed unpaired Student t-test was performed for statistical analysis.
Fig. S2

## Slide 6
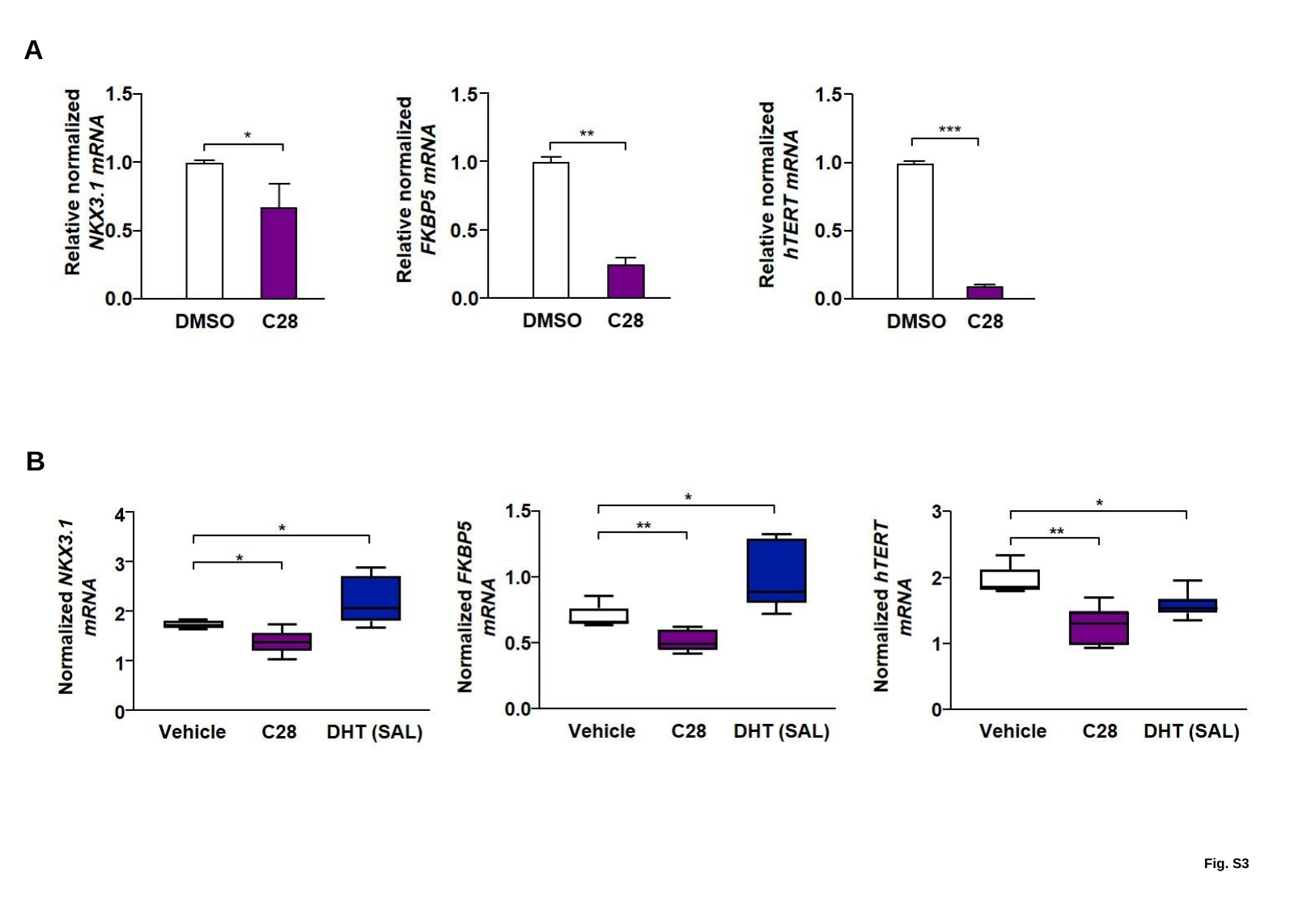

A
B
Fig. S3

## Slide 7
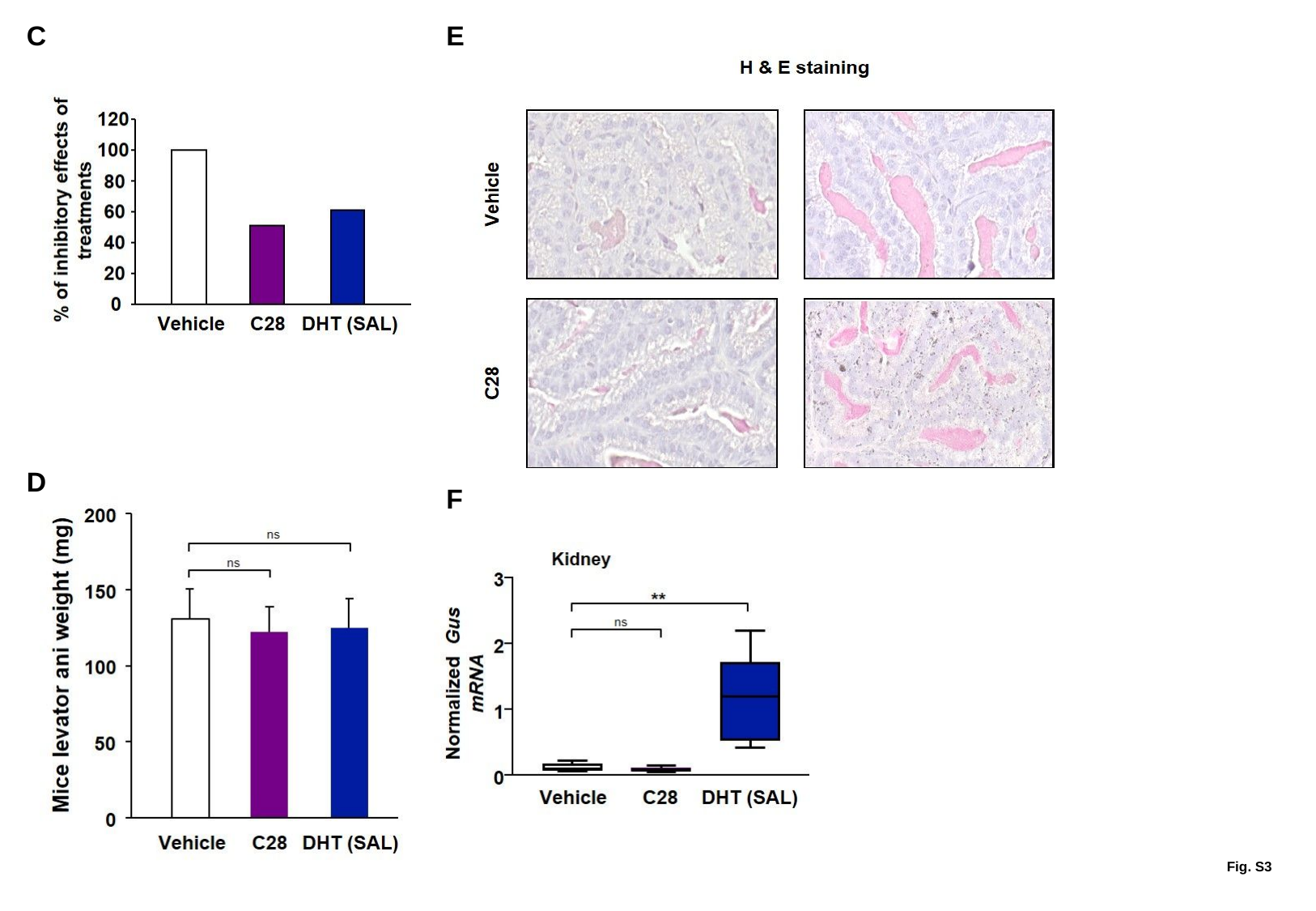

C
E
D
F
Fig. S3

## Slide 8
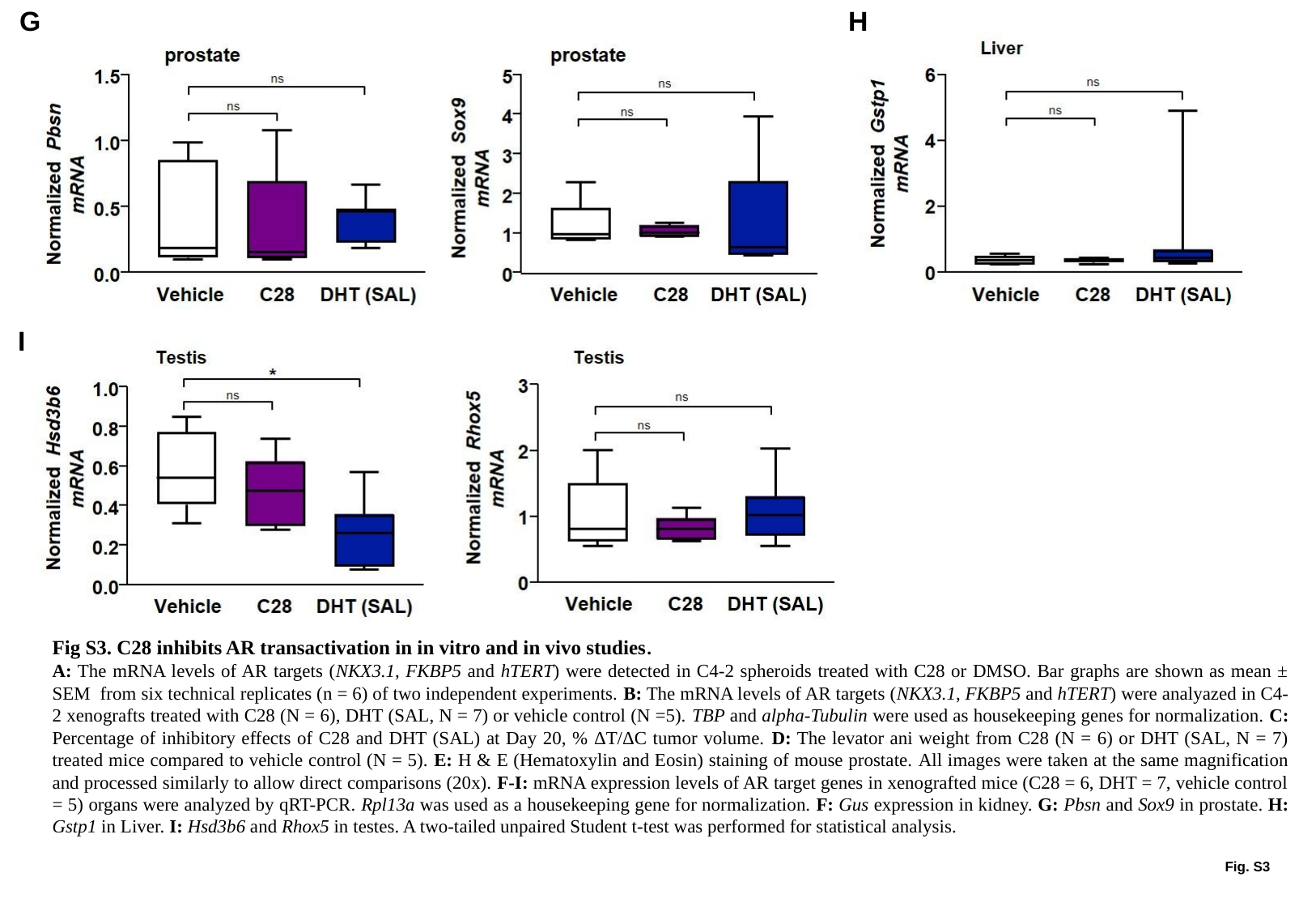

G
H
I
Fig S3. C28 inhibits AR transactivation in in vitro and in vivo studies.
A: The mRNA levels of AR targets (NKX3.1, FKBP5 and hTERT) were detected in C4-2 spheroids treated with C28 or DMSO. Bar graphs are shown as mean ± SEM from six technical replicates (n = 6) of two independent experiments. B: The mRNA levels of AR targets (NKX3.1, FKBP5 and hTERT) were analyazed in C4-2 xenografts treated with C28 (N = 6), DHT (SAL, N = 7) or vehicle control (N =5). TBP and alpha-Tubulin were used as housekeeping genes for normalization. C: Percentage of inhibitory effects of C28 and DHT (SAL) at Day 20, % ΔT/ΔC tumor volume. D: The levator ani weight from C28 (N = 6) or DHT (SAL, N = 7) treated mice compared to vehicle control (N = 5). E: H & E (Hematoxylin and Eosin) staining of mouse prostate. All images were taken at the same magnification and processed similarly to allow direct comparisons (20x). F-I: mRNA expression levels of AR target genes in xenografted mice (C28 = 6, DHT = 7, vehicle control = 5) organs were analyzed by qRT-PCR. Rpl13a was used as a housekeeping gene for normalization. F: Gus expression in kidney. G: Pbsn and Sox9 in prostate. H: Gstp1 in Liver. I: Hsd3b6 and Rhox5 in testes. A two-tailed unpaired Student t-test was performed for statistical analysis.
Fig. S3

## Slide 9
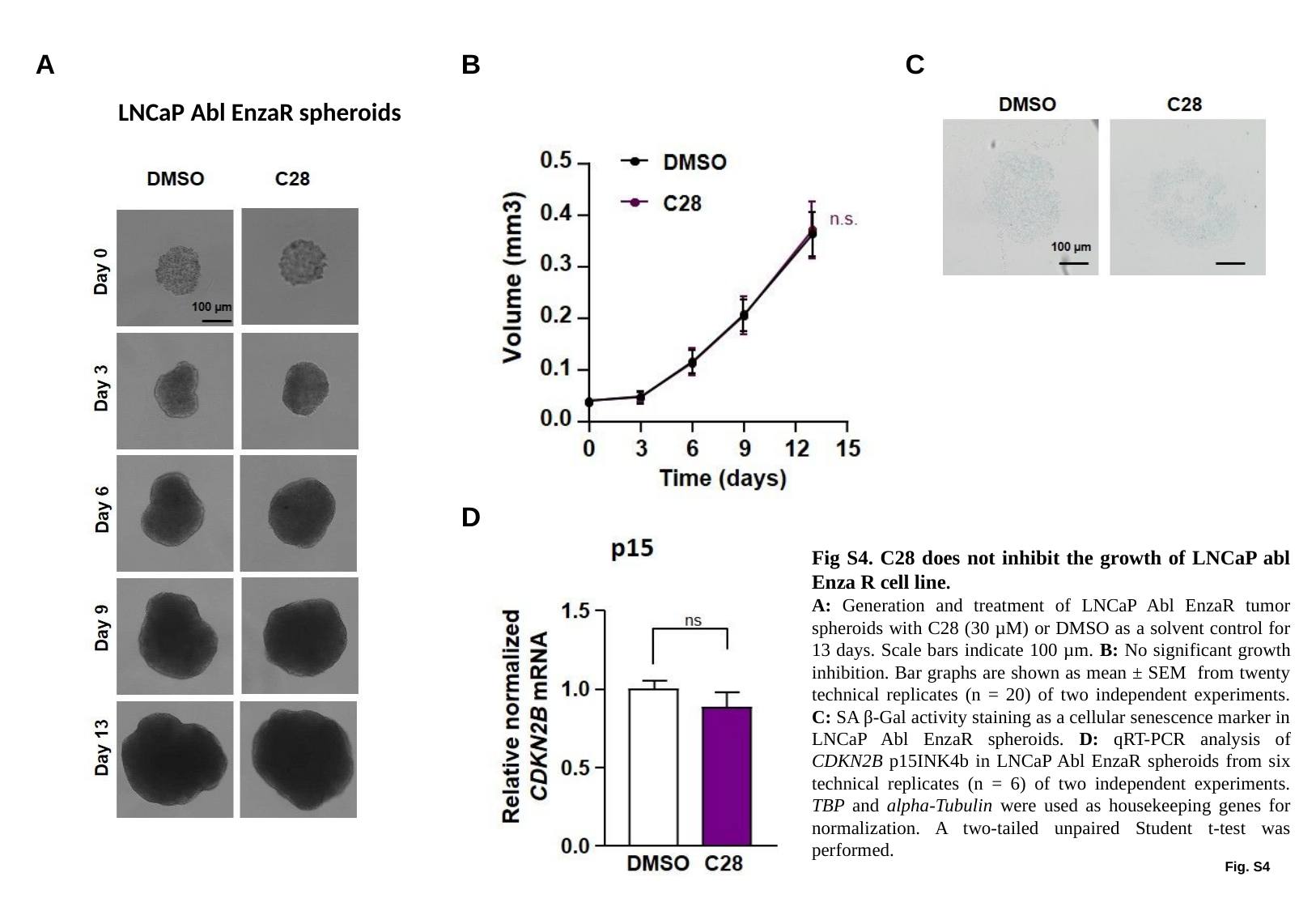

C
A
B
LNCaP Abl EnzaR spheroids
D
Fig S4. C28 does not inhibit the growth of LNCaP abl Enza R cell line.
A: Generation and treatment of LNCaP Abl EnzaR tumor spheroids with C28 (30 µM) or DMSO as a solvent control for 13 days. Scale bars indicate 100 µm. B: No significant growth inhibition. Bar graphs are shown as mean ± SEM from twenty technical replicates (n = 20) of two independent experiments. C: SA β-Gal activity staining as a cellular senescence marker in LNCaP Abl EnzaR spheroids. D: qRT-PCR analysis of CDKN2B p15INK4b in LNCaP Abl EnzaR spheroids from six technical replicates (n = 6) of two independent experiments. TBP and alpha-Tubulin were used as housekeeping genes for normalization. A two-tailed unpaired Student t-test was performed.
Fig. S4

## Slide 10
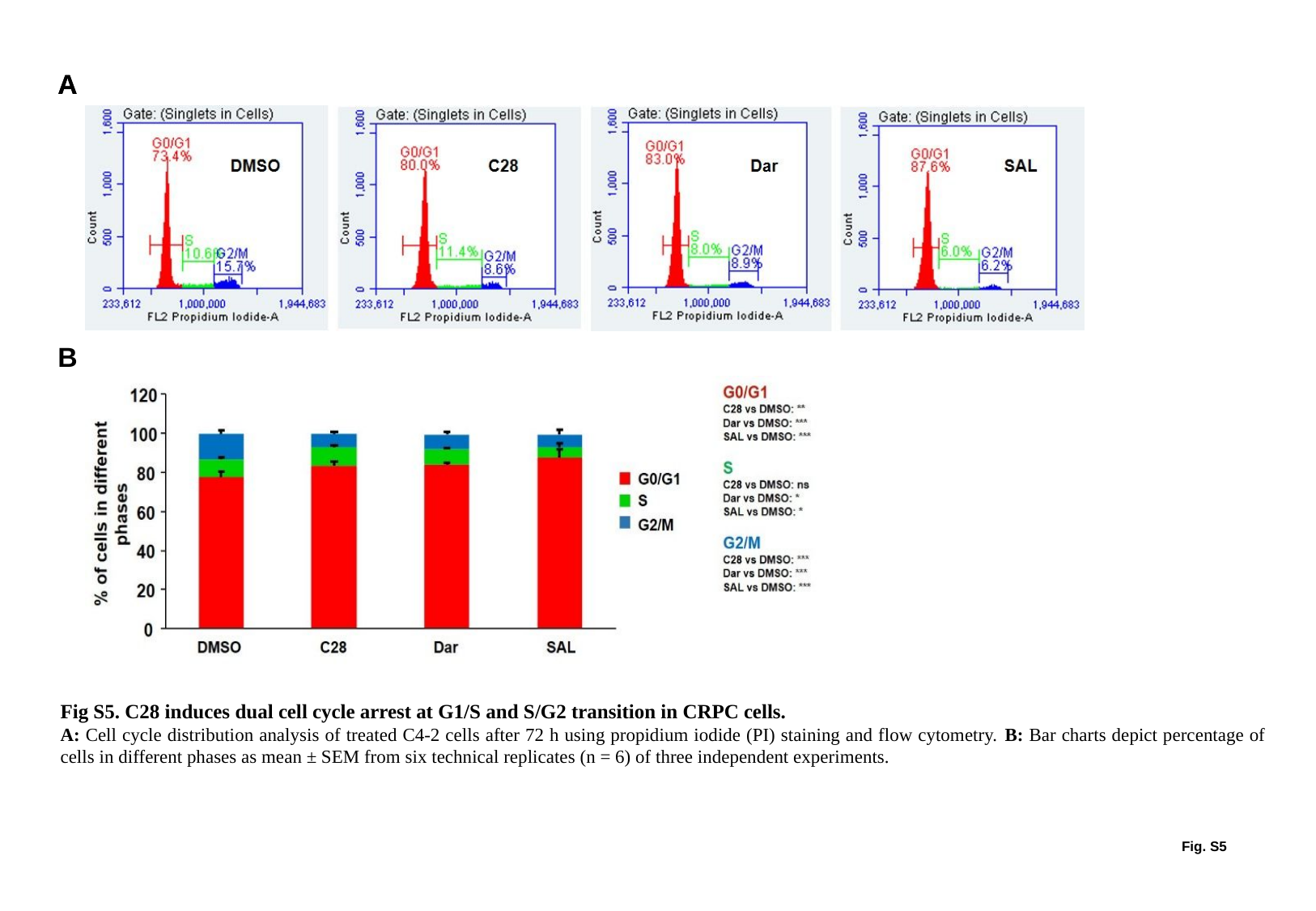

A
A
B
Fig S5. C28 induces dual cell cycle arrest at G1/S and S/G2 transition in CRPC cells.
A: Cell cycle distribution analysis of treated C4-2 cells after 72 h using propidium iodide (PI) staining and flow cytometry. B: Bar charts depict percentage of cells in different phases as mean ± SEM from six technical replicates (n = 6) of three independent experiments.
Fig. S5

## Slide 11
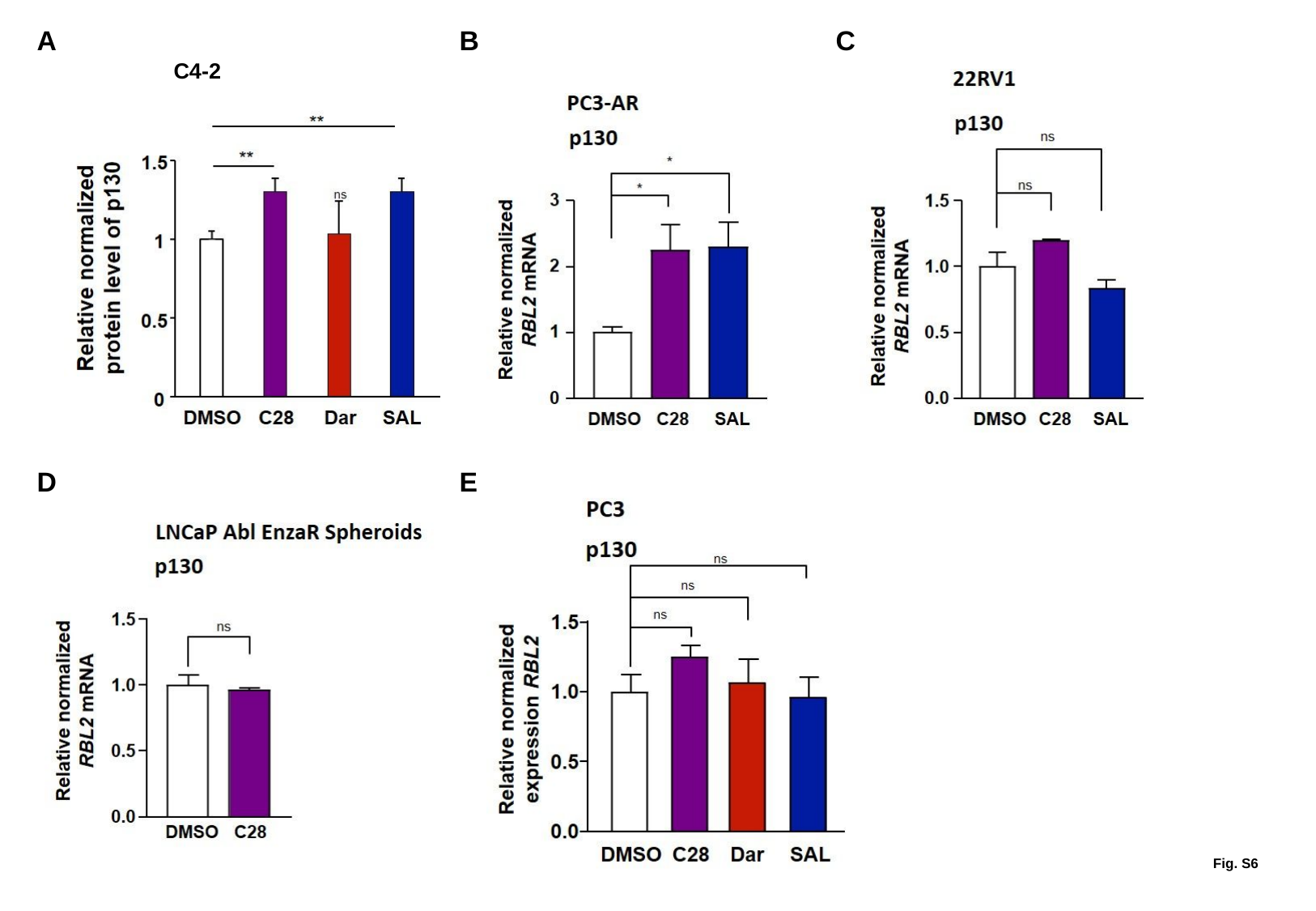

B
C
A
C4-2
E
D
Fig. S6

## Slide 12
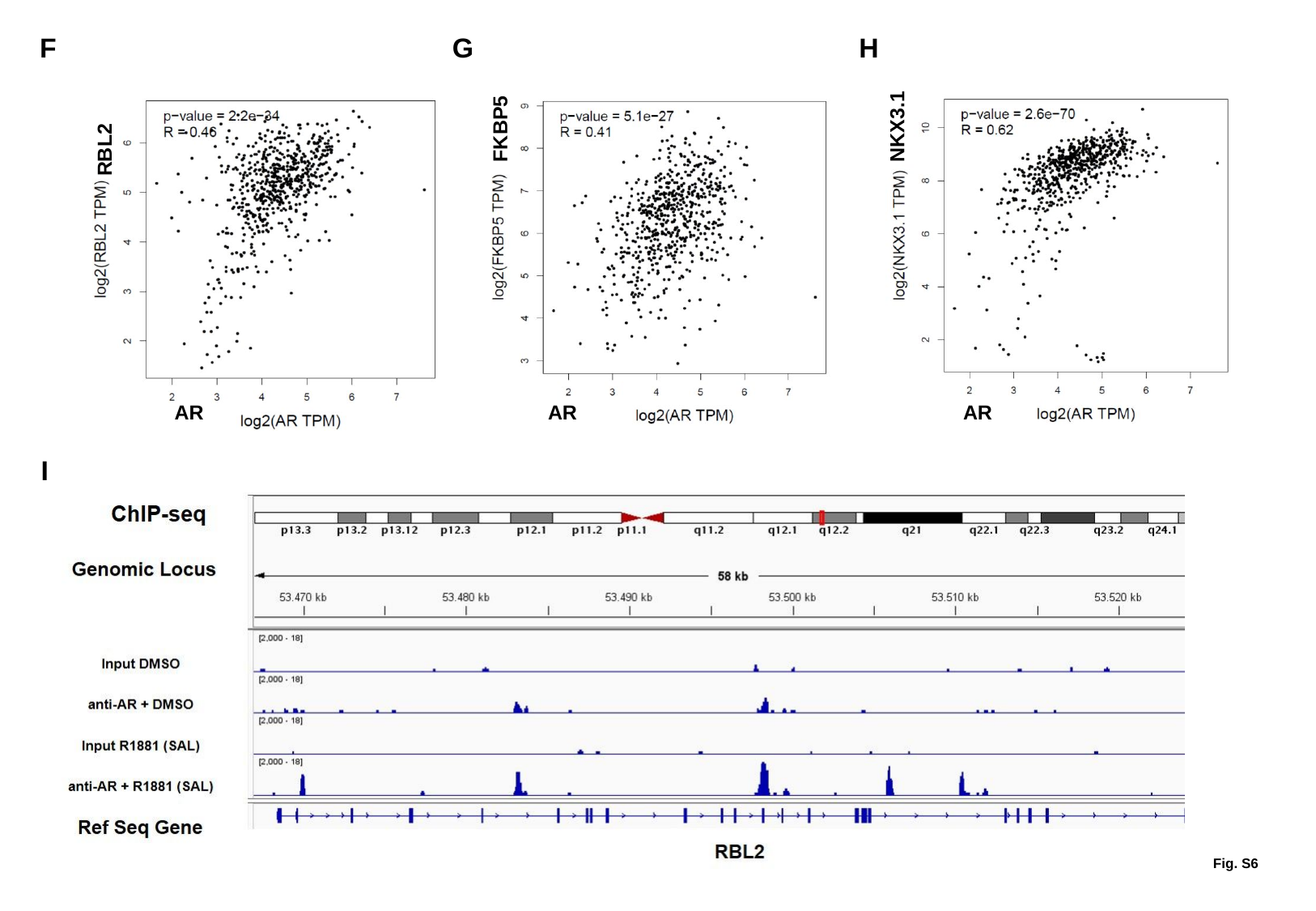

F
G
H
NKX3.1
AR
FKBP5
AR
RBL2
AR
I
Fig. S6

## Slide 13
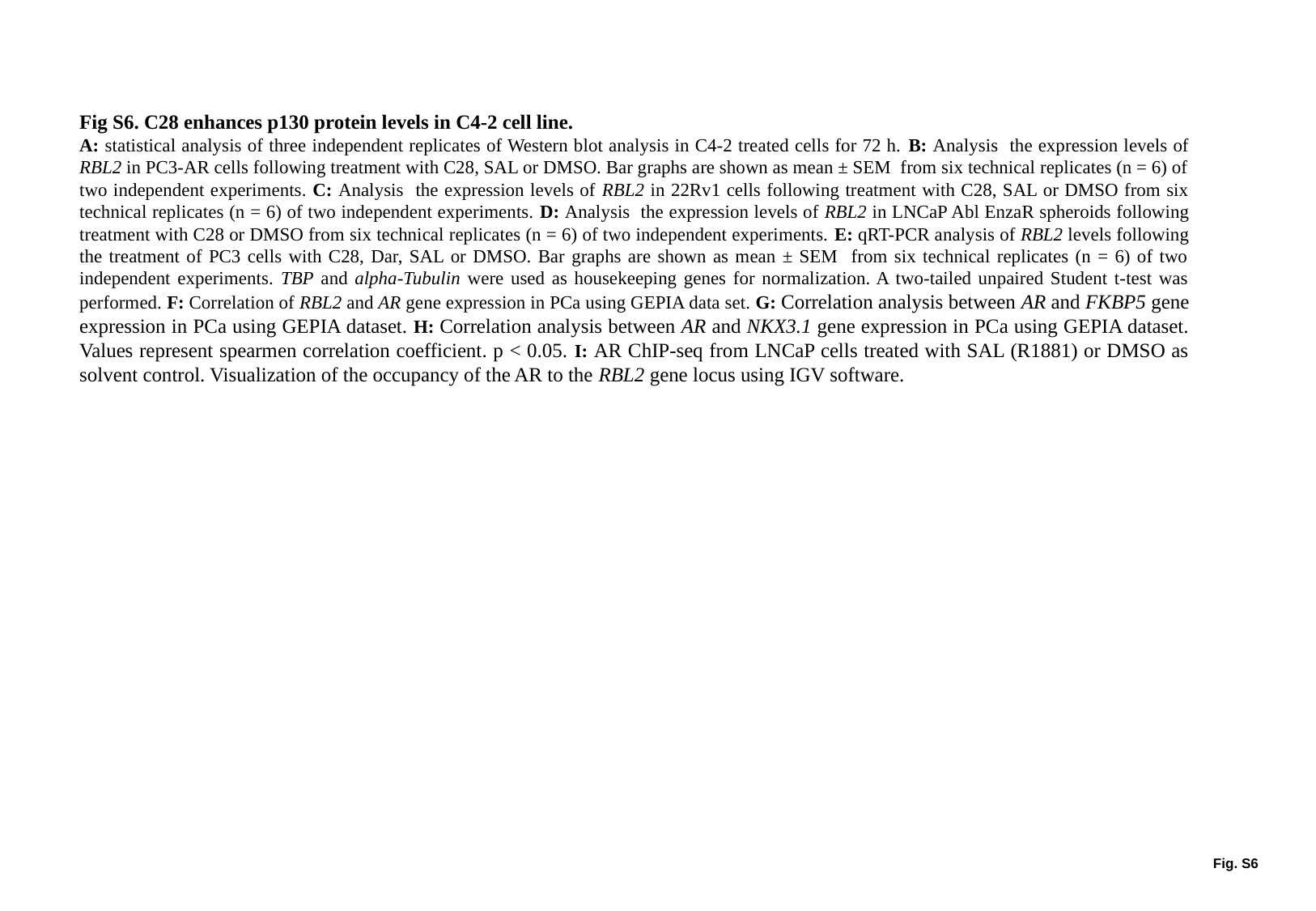

Fig S6. C28 enhances p130 protein levels in C4-2 cell line.
A: statistical analysis of three independent replicates of Western blot analysis in C4-2 treated cells for 72 h. B: Analysis the expression levels of RBL2 in PC3-AR cells following treatment with C28, SAL or DMSO. Bar graphs are shown as mean ± SEM from six technical replicates (n = 6) of two independent experiments. C: Analysis the expression levels of RBL2 in 22Rv1 cells following treatment with C28, SAL or DMSO from six technical replicates (n = 6) of two independent experiments. D: Analysis the expression levels of RBL2 in LNCaP Abl EnzaR spheroids following treatment with C28 or DMSO from six technical replicates (n = 6) of two independent experiments. E: qRT-PCR analysis of RBL2 levels following the treatment of PC3 cells with C28, Dar, SAL or DMSO. Bar graphs are shown as mean ± SEM from six technical replicates (n = 6) of two independent experiments. TBP and alpha-Tubulin were used as housekeeping genes for normalization. A two-tailed unpaired Student t-test was performed. F: Correlation of RBL2 and AR gene expression in PCa using GEPIA data set. G: Correlation analysis between AR and FKBP5 gene expression in PCa using GEPIA dataset. H: Correlation analysis between AR and NKX3.1 gene expression in PCa using GEPIA dataset. Values represent spearmen correlation coefficient. p < 0.05. I: AR ChIP-seq from LNCaP cells treated with SAL (R1881) or DMSO as solvent control. Visualization of the occupancy of the AR to the RBL2 gene locus using IGV software.
Fig. S6

## Slide 14
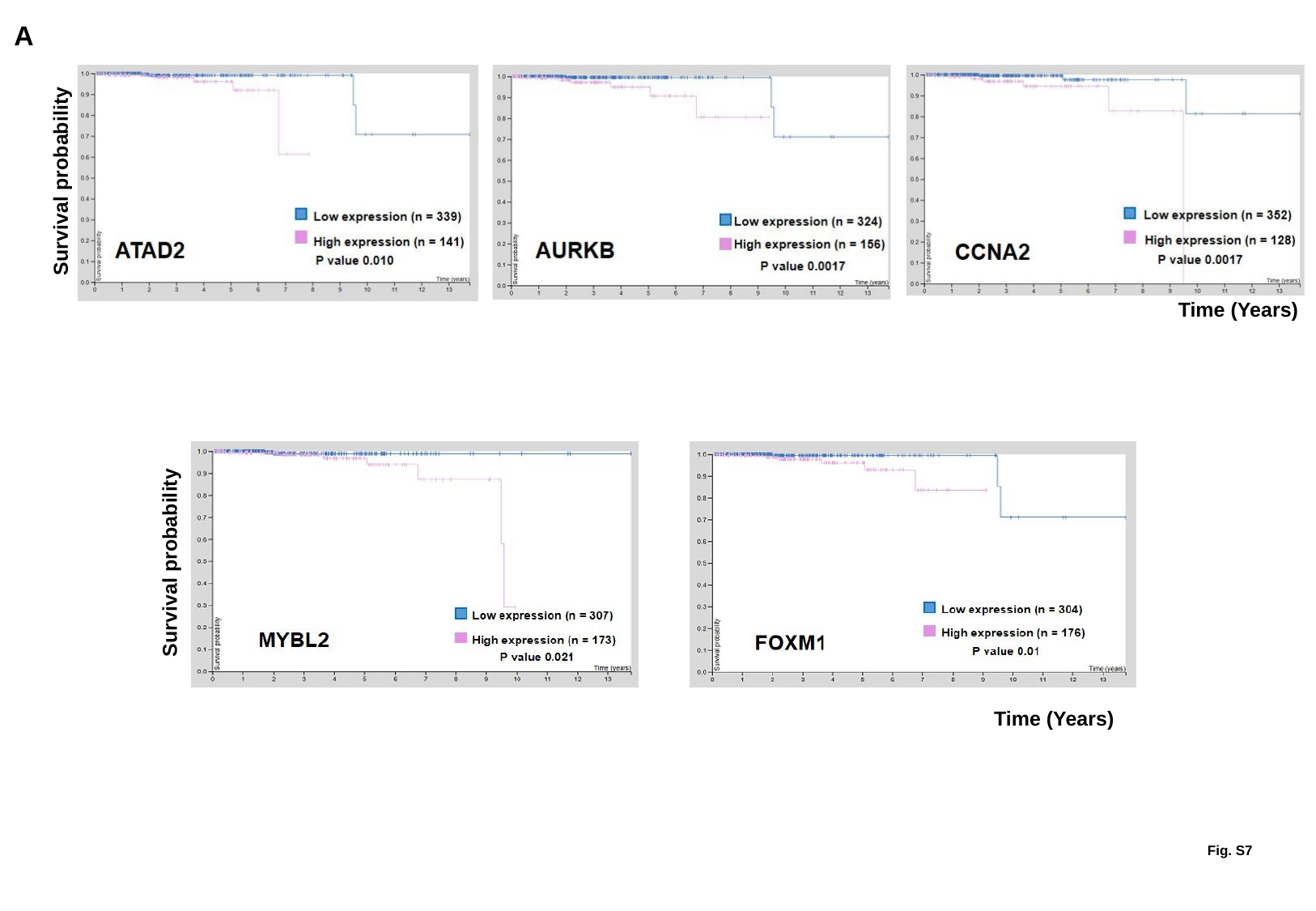

A
Survival probability
Time (Years)
Survival probability
Time (Years)
Fig. S7

## Slide 15
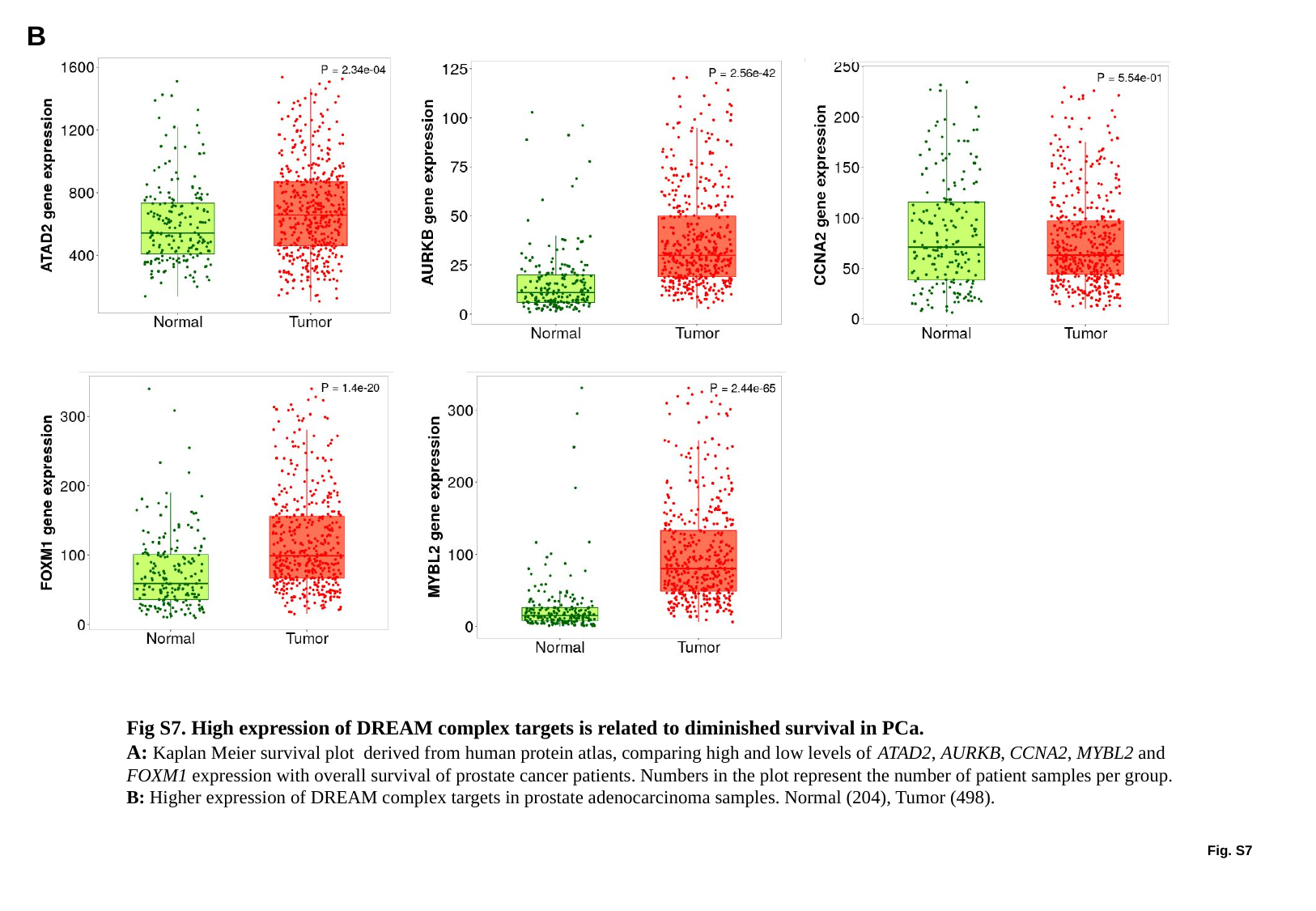

B
Fig S7. High expression of DREAM complex targets is related to diminished survival in PCa.
A: Kaplan Meier survival plot derived from human protein atlas, comparing high and low levels of ATAD2, AURKB, CCNA2, MYBL2 and FOXM1 expression with overall survival of prostate cancer patients. Numbers in the plot represent the number of patient samples per group. B: Higher expression of DREAM complex targets in prostate adenocarcinoma samples. Normal (204), Tumor (498).
Fig. S7

## Slide 16
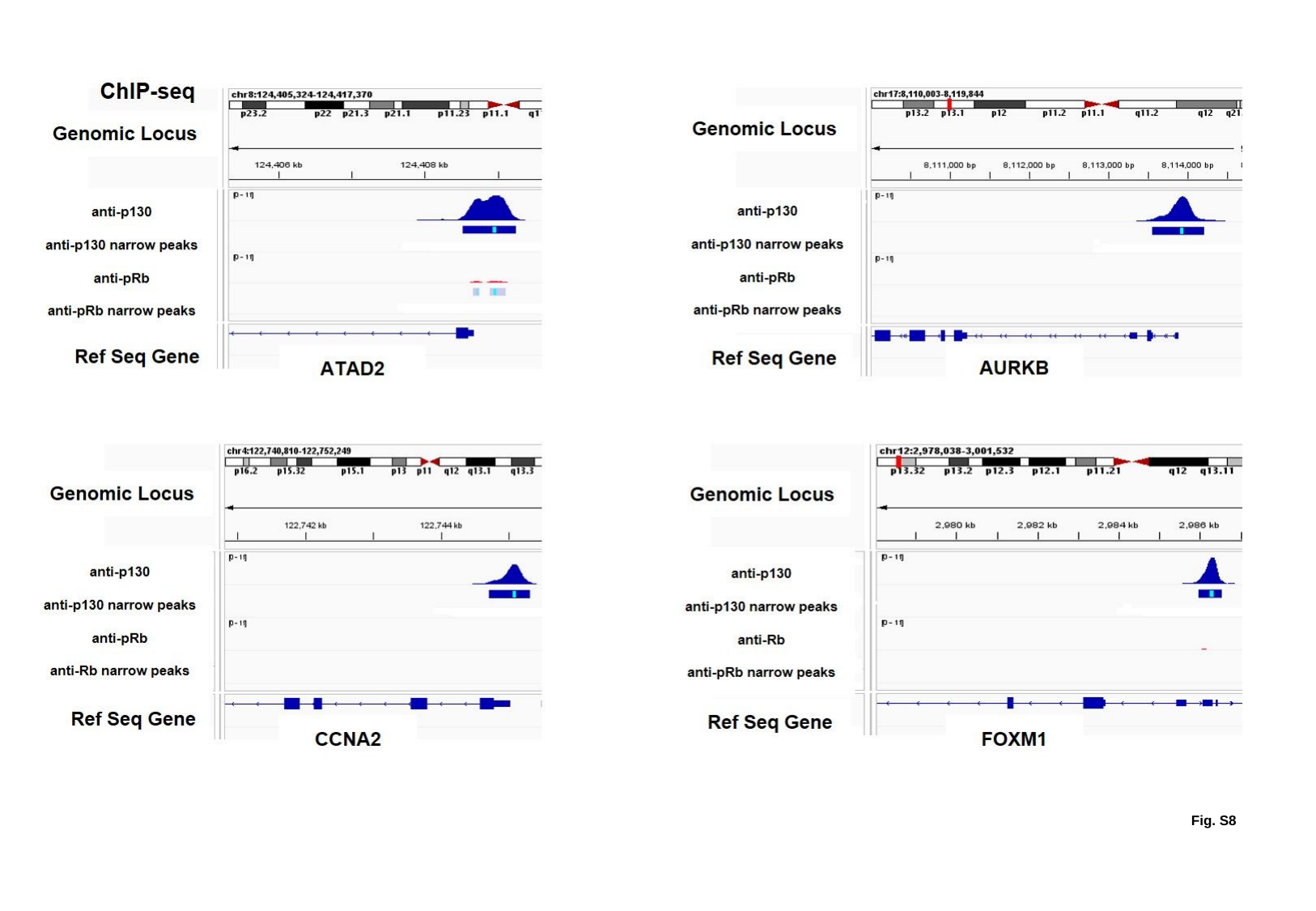

Fig. S8

## Slide 17
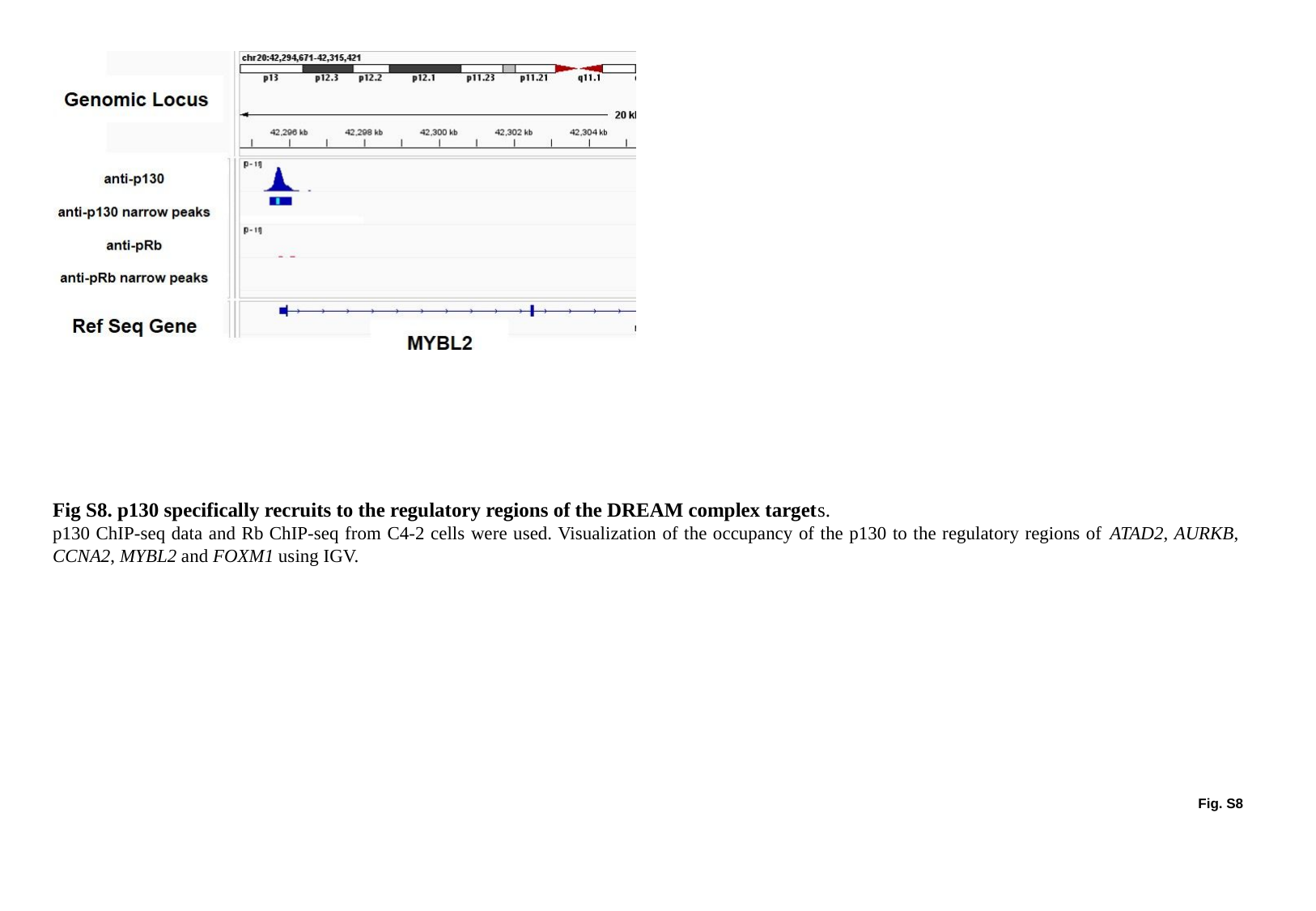

Fig S8. p130 specifically recruits to the regulatory regions of the DREAM complex targets.
p130 ChIP-seq data and Rb ChIP-seq from C4-2 cells were used. Visualization of the occupancy of the p130 to the regulatory regions of ATAD2, AURKB, CCNA2, MYBL2 and FOXM1 using IGV.
Fig. S8

## Slide 18
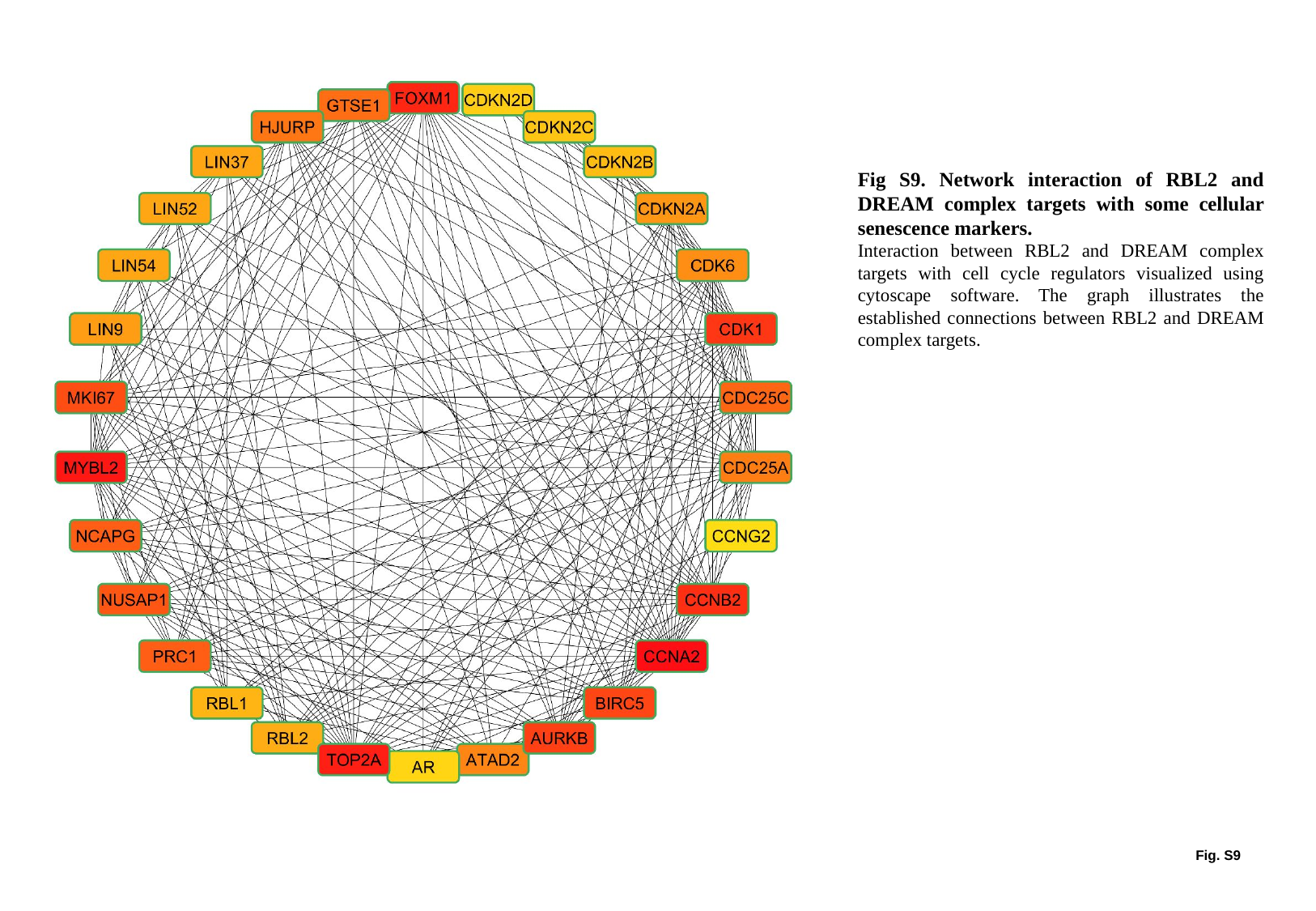

Fig S9. Network interaction of RBL2 and DREAM complex targets with some cellular senescence markers.
Interaction between RBL2 and DREAM complex targets with cell cycle regulators visualized using cytoscape software. The graph illustrates the established connections between RBL2 and DREAM complex targets.
Fig. S9

## Slide 19
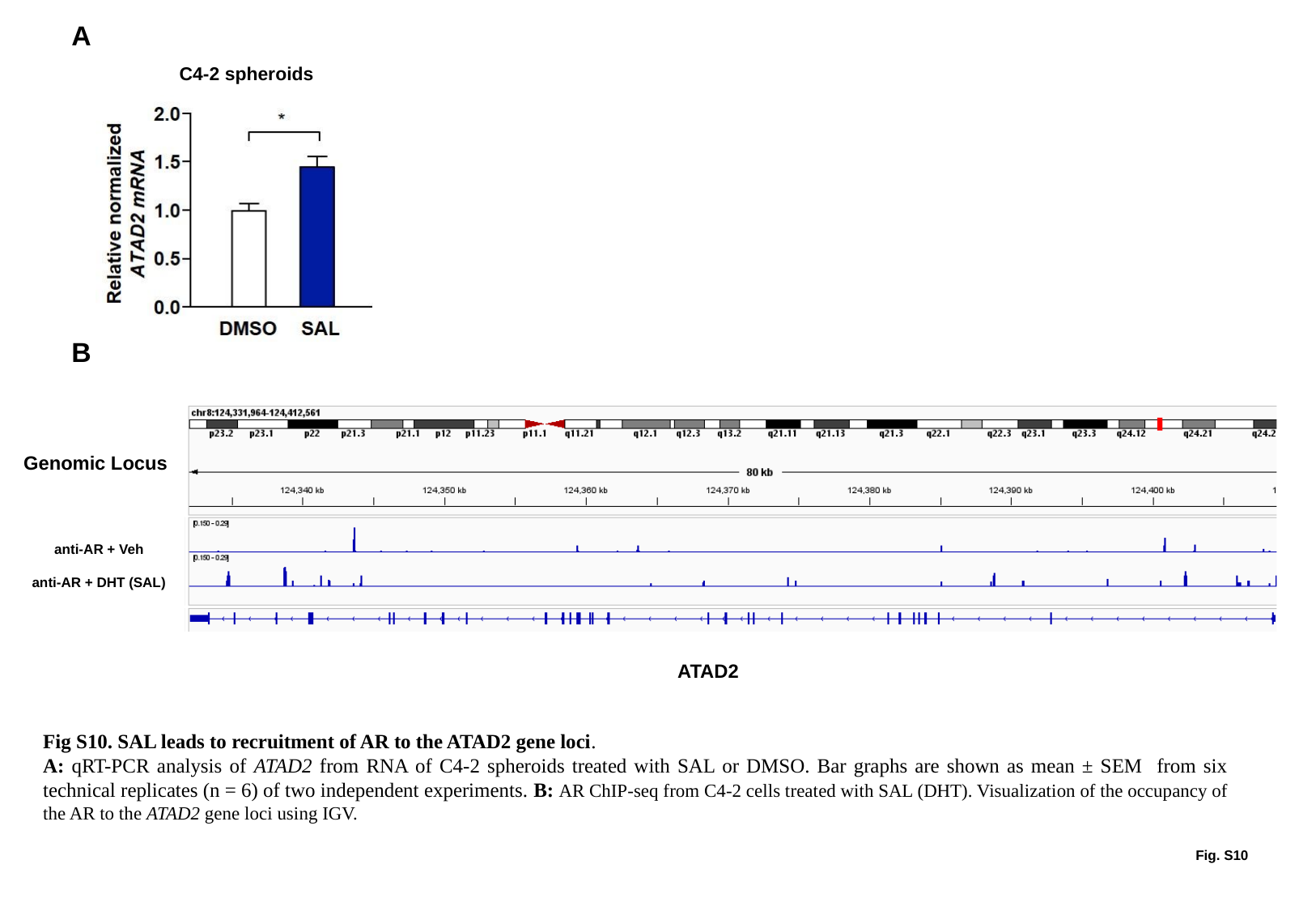

A
C4-2 spheroids
B
Genomic Locus
anti-AR + Veh
anti-AR + DHT (SAL)
ATAD2
Fig S10. SAL leads to recruitment of AR to the ATAD2 gene loci.
A: qRT-PCR analysis of ATAD2 from RNA of C4-2 spheroids treated with SAL or DMSO. Bar graphs are shown as mean ± SEM from six technical replicates (n = 6) of two independent experiments. B: AR ChIP-seq from C4-2 cells treated with SAL (DHT). Visualization of the occupancy of the AR to the ATAD2 gene loci using IGV.
Fig. S10

## Slide 20
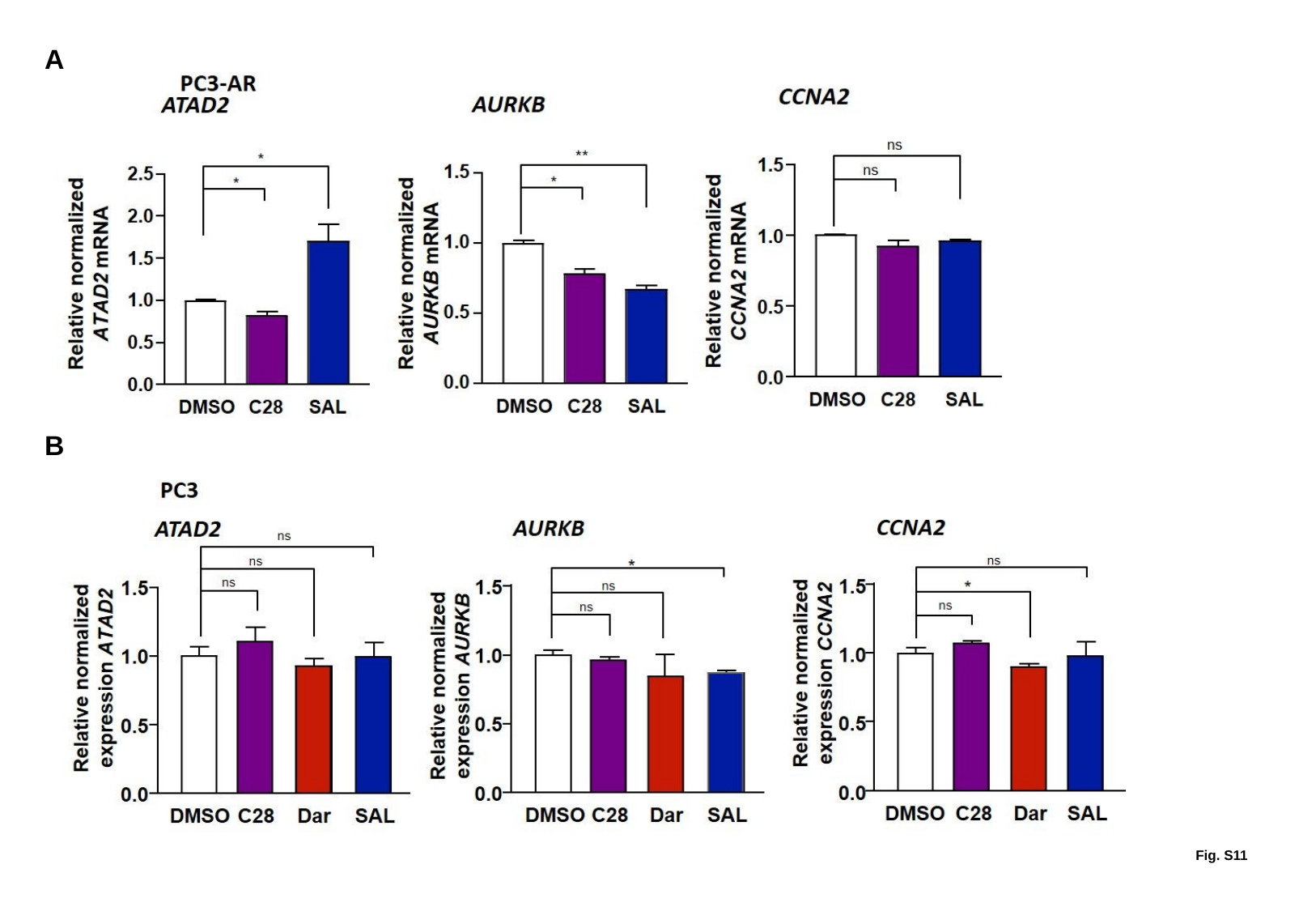

A
B
Fig. S11

## Slide 21
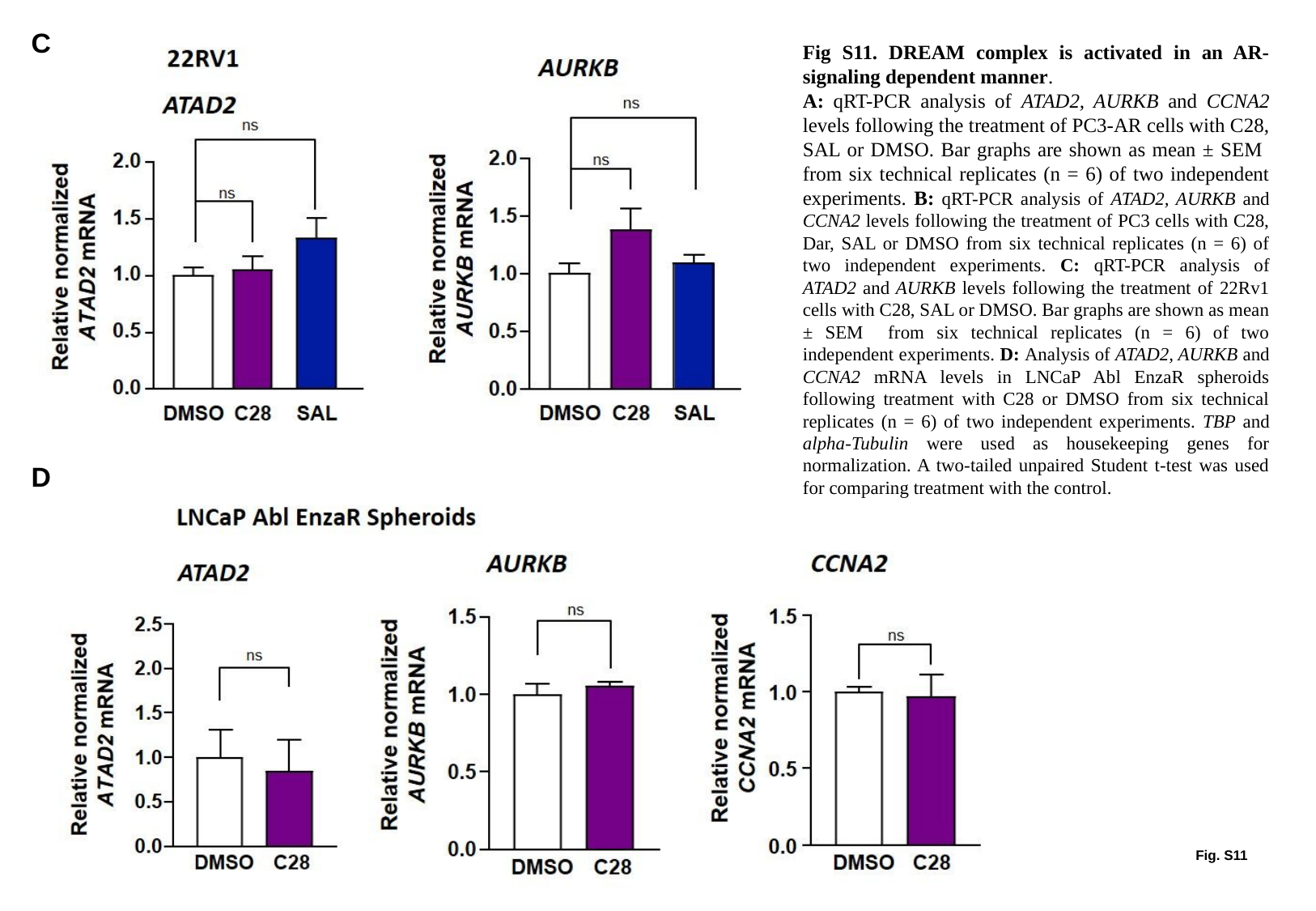

C
Fig S11. DREAM complex is activated in an AR-signaling dependent manner.
A: qRT-PCR analysis of ATAD2, AURKB and CCNA2 levels following the treatment of PC3-AR cells with C28, SAL or DMSO. Bar graphs are shown as mean ± SEM from six technical replicates (n = 6) of two independent experiments. B: qRT-PCR analysis of ATAD2, AURKB and CCNA2 levels following the treatment of PC3 cells with C28, Dar, SAL or DMSO from six technical replicates (n = 6) of two independent experiments. C: qRT-PCR analysis of ATAD2 and AURKB levels following the treatment of 22Rv1 cells with C28, SAL or DMSO. Bar graphs are shown as mean ± SEM from six technical replicates (n = 6) of two independent experiments. D: Analysis of ATAD2, AURKB and CCNA2 mRNA levels in LNCaP Abl EnzaR spheroids following treatment with C28 or DMSO from six technical replicates (n = 6) of two independent experiments. TBP and alpha-Tubulin were used as housekeeping genes for normalization. A two-tailed unpaired Student t-test was used for comparing treatment with the control.
D
Fig. S11

## Slide 22
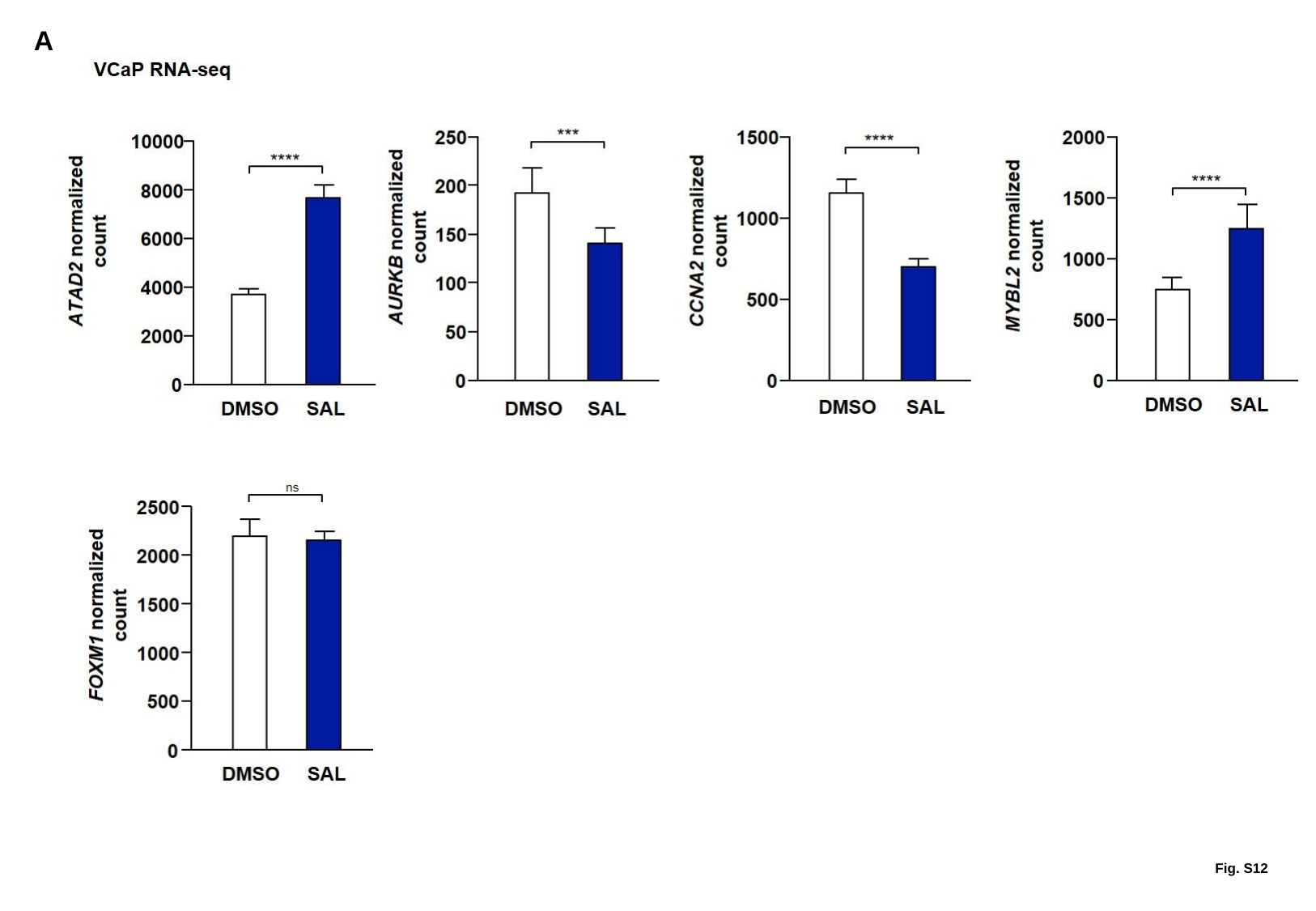

A
Fig. S12

## Slide 23
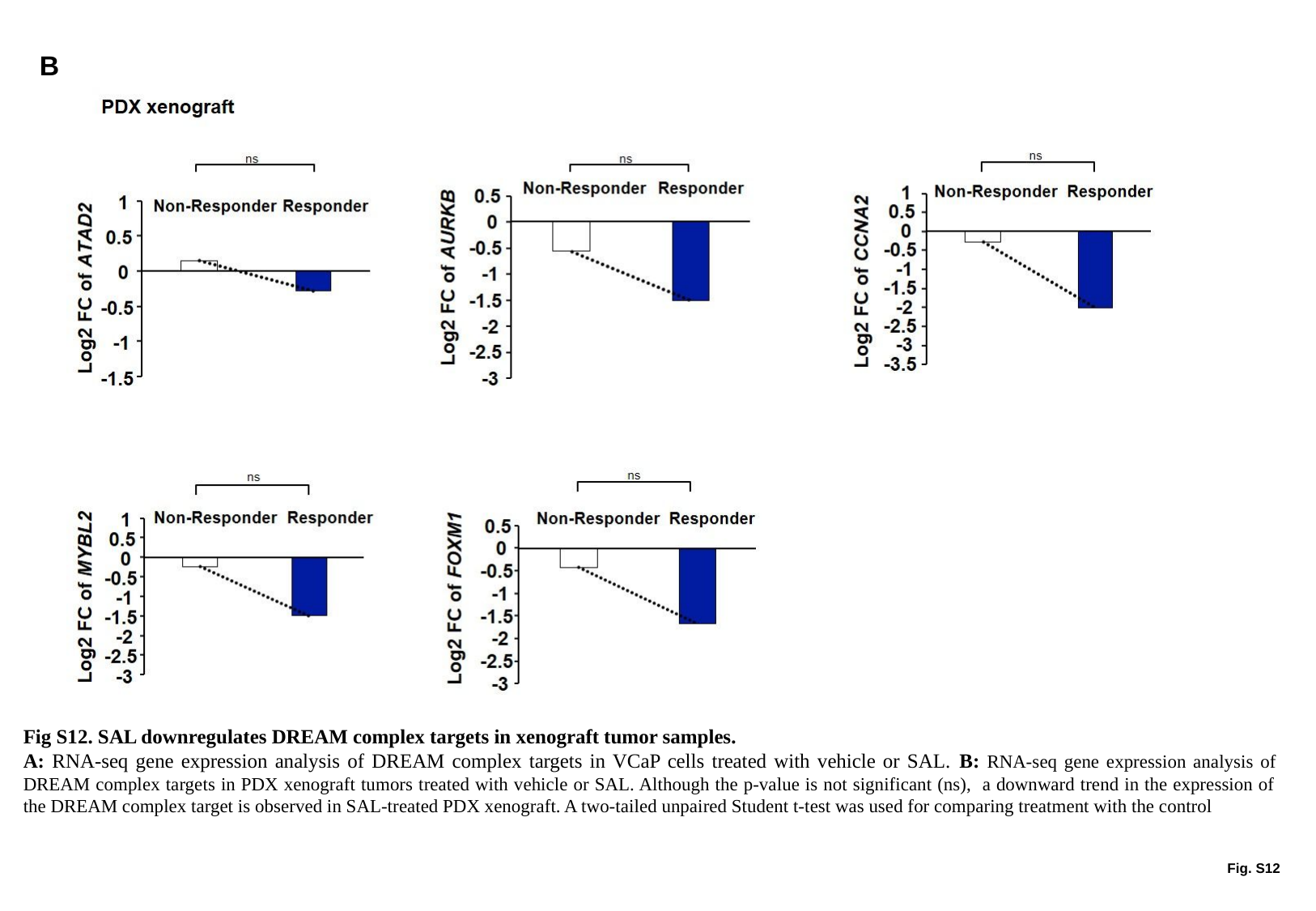

B
Fig S12. SAL downregulates DREAM complex targets in xenograft tumor samples.
A: RNA-seq gene expression analysis of DREAM complex targets in VCaP cells treated with vehicle or SAL. B: RNA-seq gene expression analysis of DREAM complex targets in PDX xenograft tumors treated with vehicle or SAL. Although the p-value is not significant (ns), a downward trend in the expression of the DREAM complex target is observed in SAL-treated PDX xenograft. A two-tailed unpaired Student t-test was used for comparing treatment with the control
Fig. S12

## Slide 24
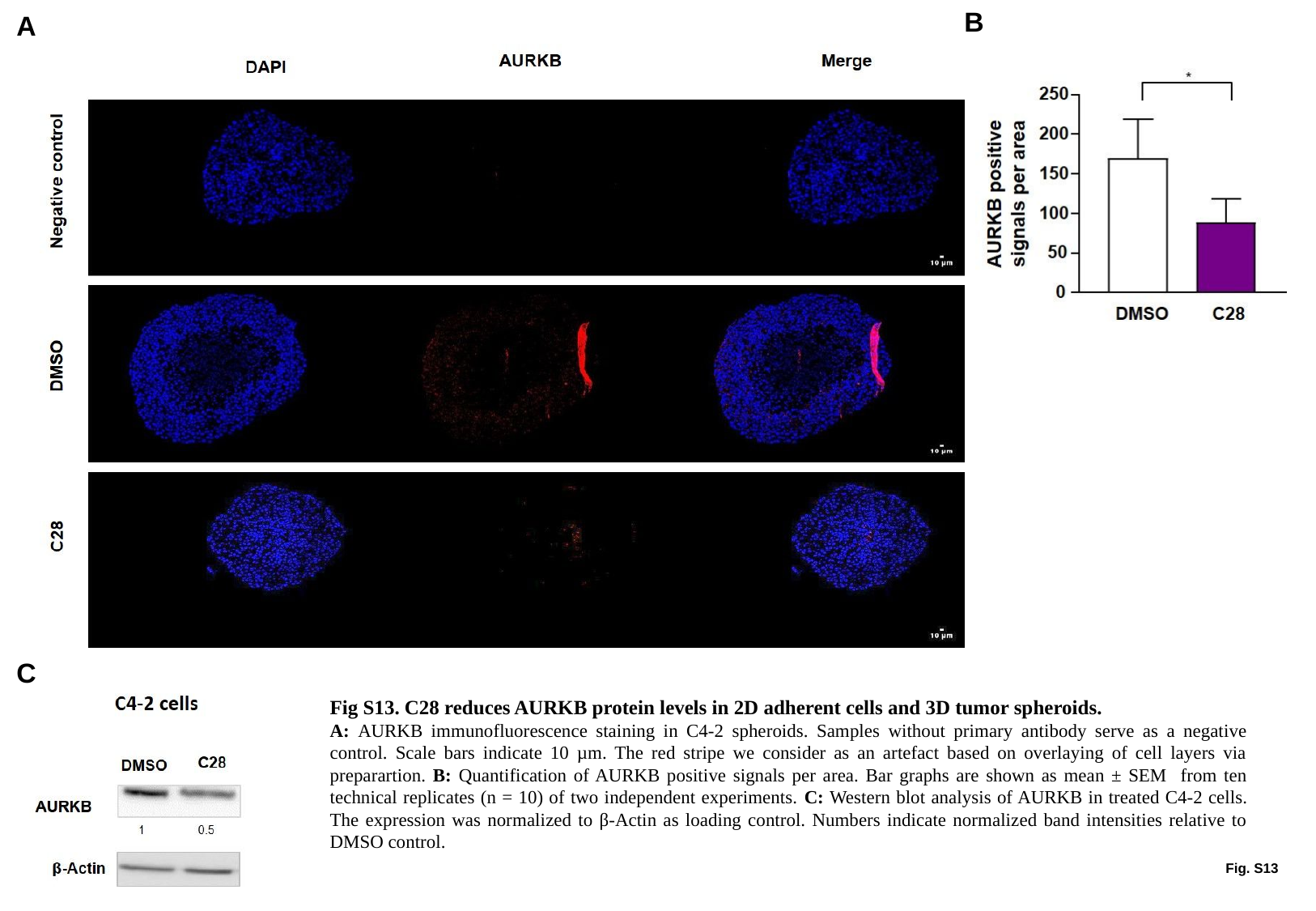

B
A
C
Fig S13. C28 reduces AURKB protein levels in 2D adherent cells and 3D tumor spheroids.
A: AURKB immunofluorescence staining in C4-2 spheroids. Samples without primary antibody serve as a negative control. Scale bars indicate 10 µm. The red stripe we consider as an artefact based on overlaying of cell layers via preparartion. B: Quantification of AURKB positive signals per area. Bar graphs are shown as mean ± SEM from ten technical replicates (n = 10) of two independent experiments. C: Western blot analysis of AURKB in treated C4-2 cells. The expression was normalized to β-Actin as loading control. Numbers indicate normalized band intensities relative to DMSO control.
Fig. S13

## Slide 25
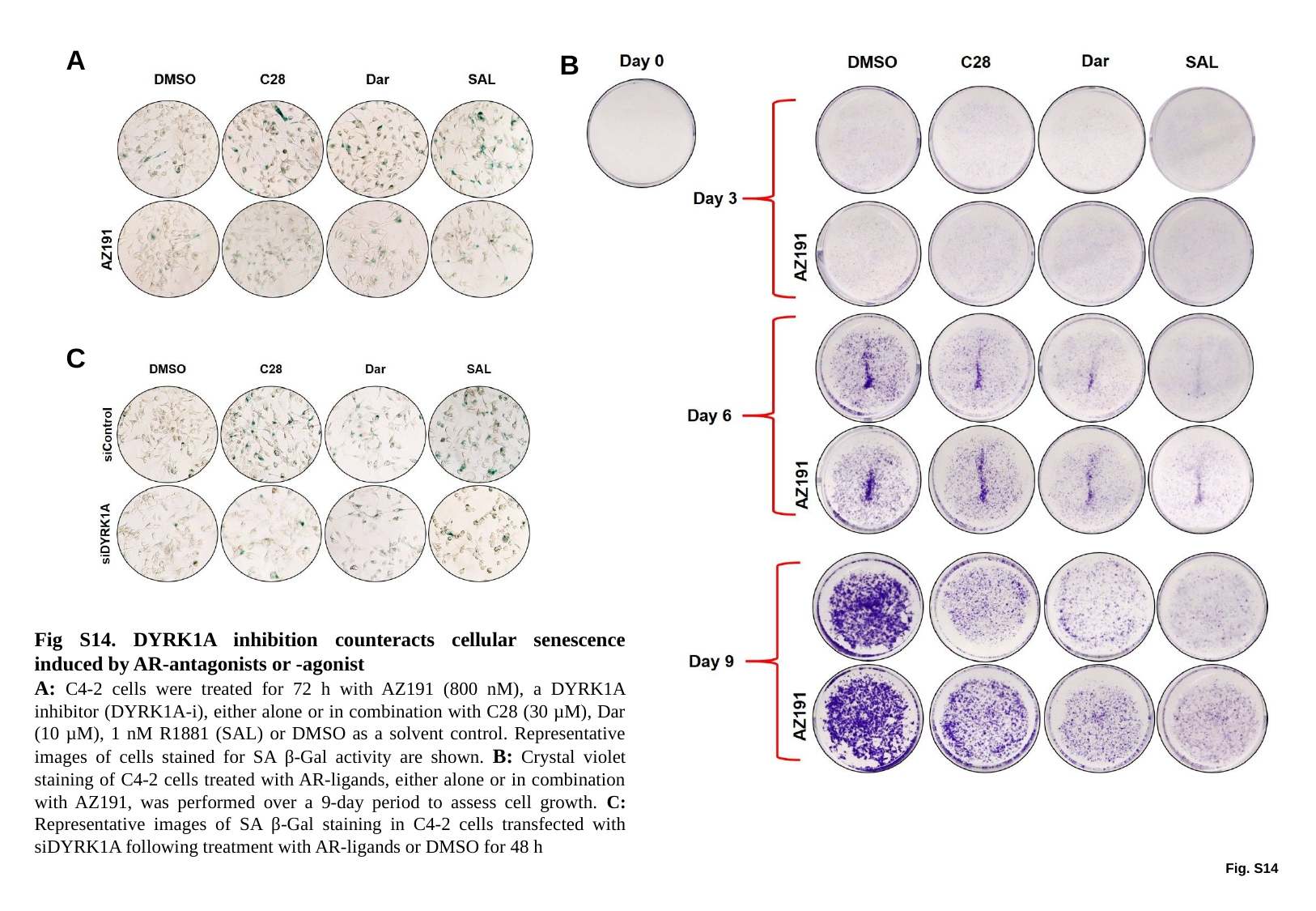

A
B
C
Fig S14. DYRK1A inhibition counteracts cellular senescence induced by AR-antagonists or -agonist
A: C4-2 cells were treated for 72 h with AZ191 (800 nM), a DYRK1A inhibitor (DYRK1A-i), either alone or in combination with C28 (30 µM), Dar (10 µM), 1 nM R1881 (SAL) or DMSO as a solvent control. Representative images of cells stained for SA β-Gal activity are shown. B: Crystal violet staining of C4-2 cells treated with AR-ligands, either alone or in combination with AZ191, was performed over a 9-day period to assess cell growth. C: Representative images of SA β-Gal staining in C4-2 cells transfected with siDYRK1A following treatment with AR-ligands or DMSO for 48 h
Fig. S14

## Slide 26
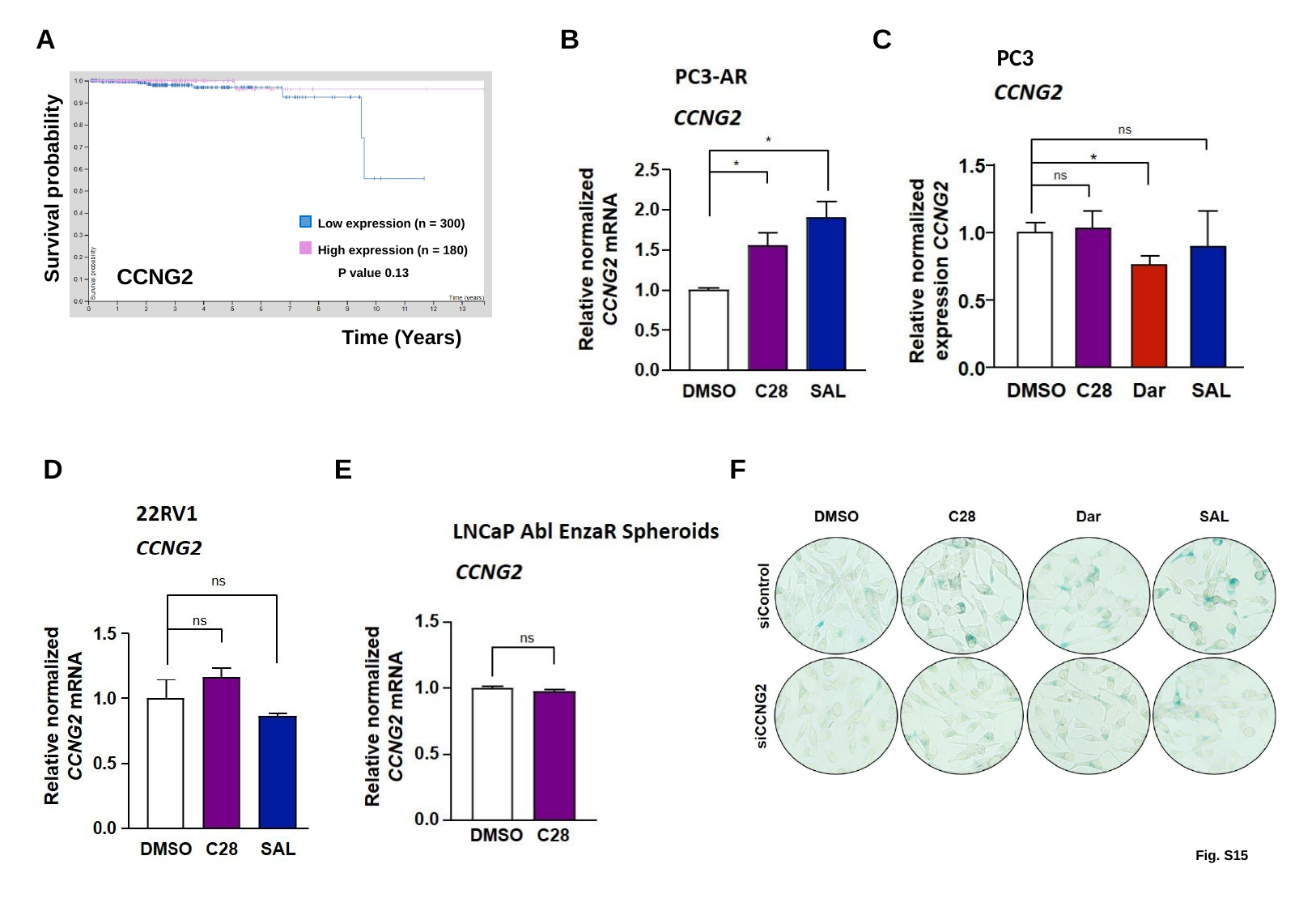

B
C
A
PC3
Survival probability
Low expression (n = 300)
High expression (n = 180)
CCNG2
P value 0.13
Time (Years)
D
E
F
Fig. S15

## Slide 27
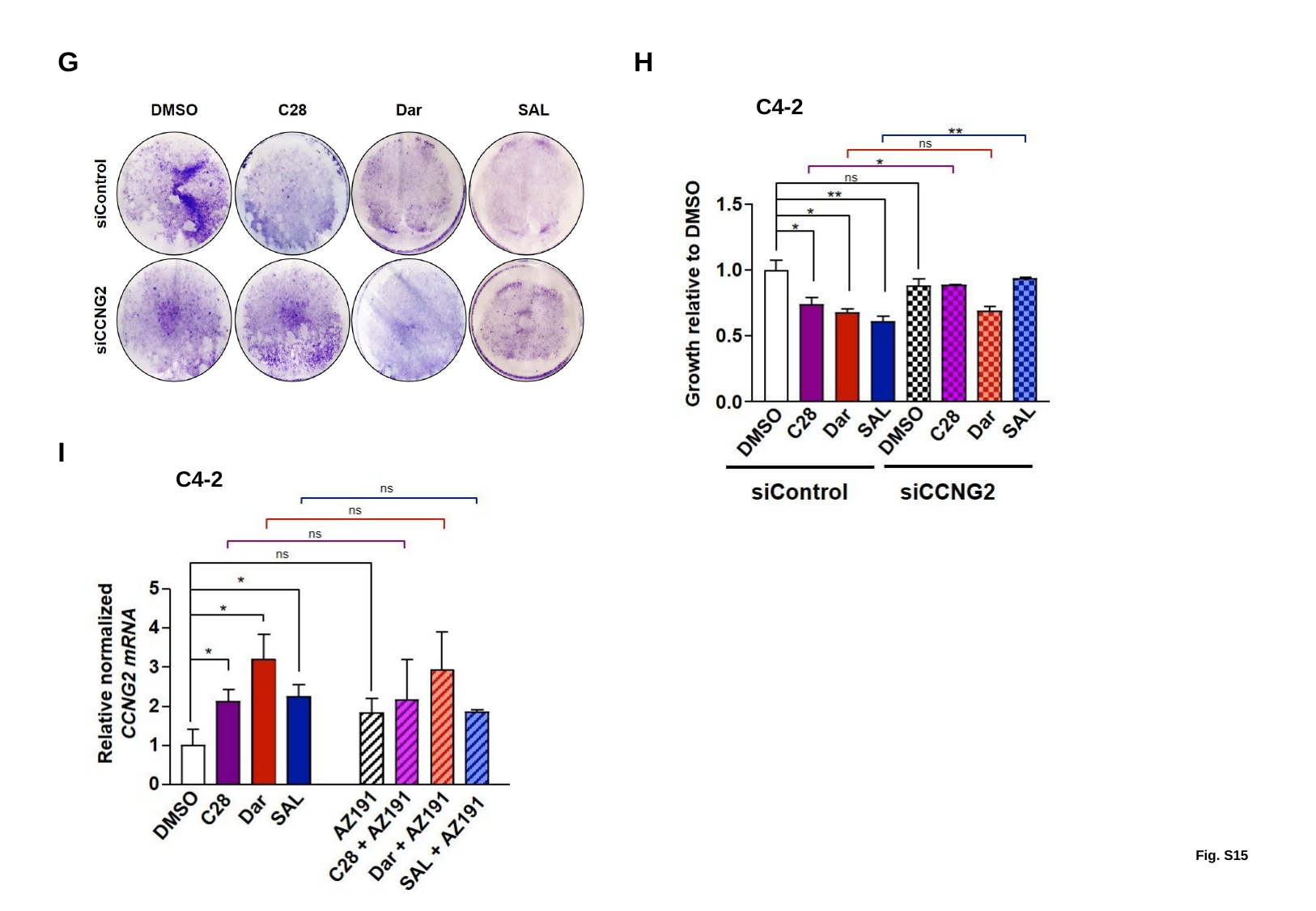

G
H
C4-2
I
C4-2
Fig. S15

## Slide 28
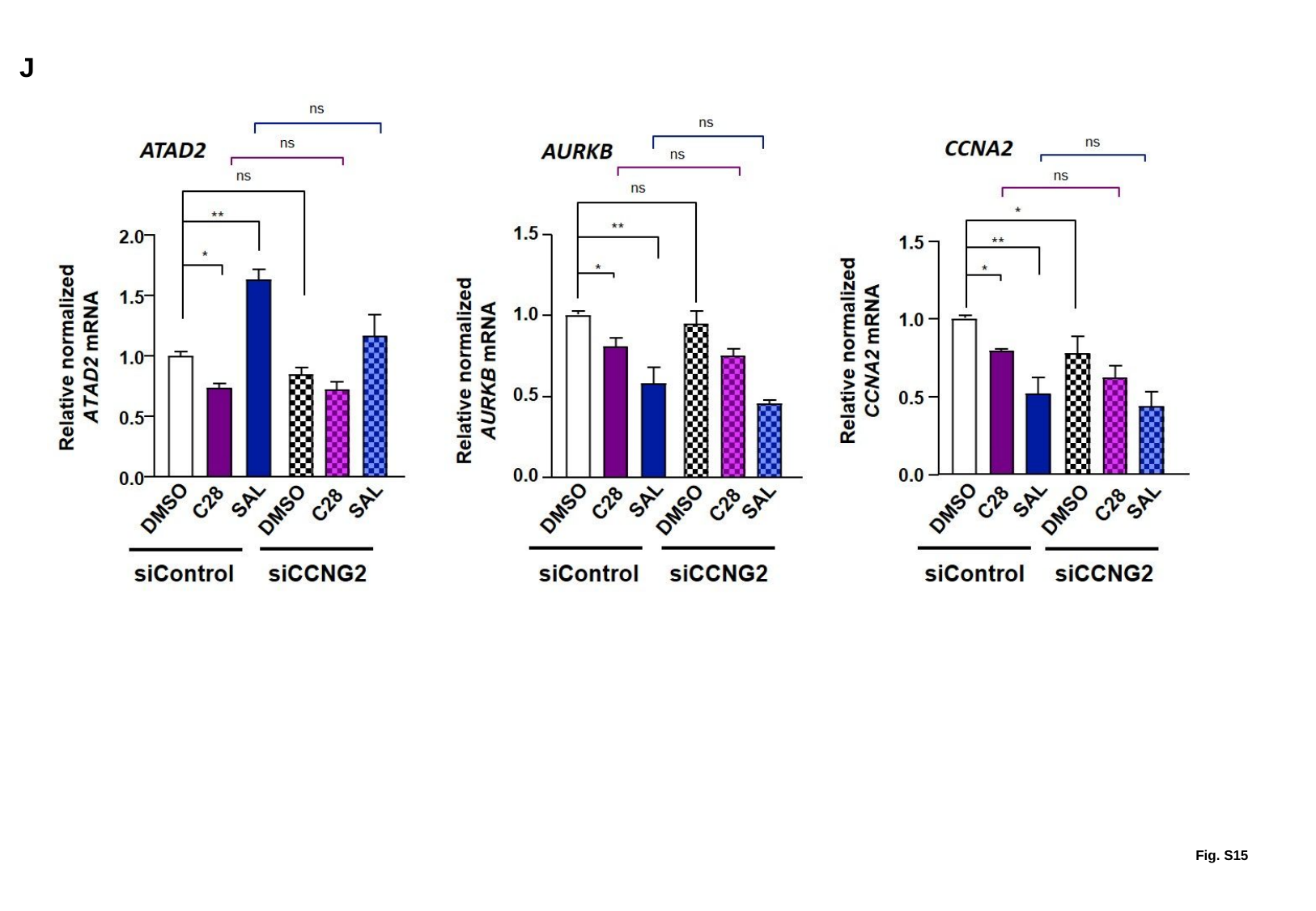

J
Fig. S15

## Slide 29
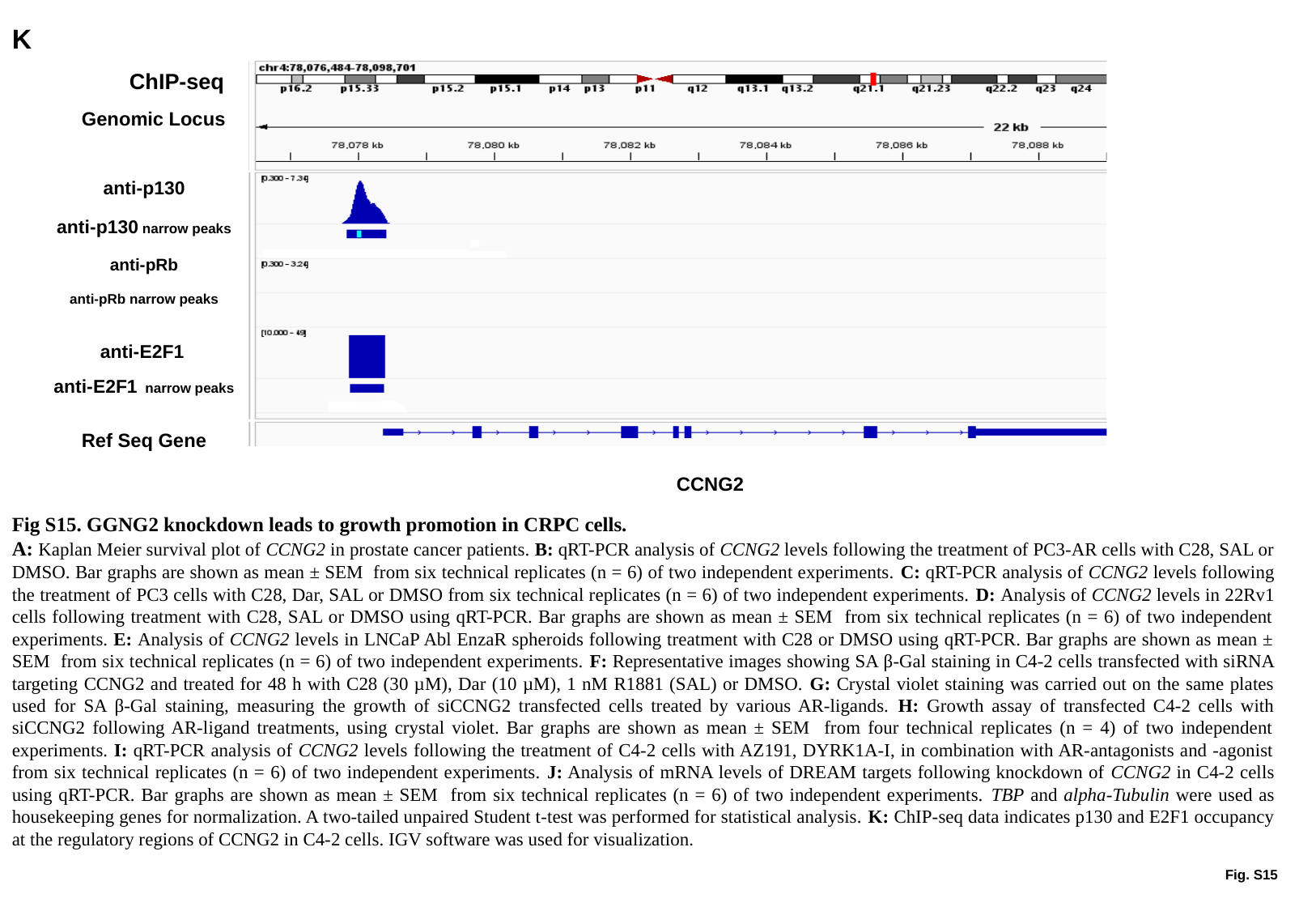

K
ChIP-seq
Genomic Locus
anti-p130
anti-p130 narrow peaks
anti-pRb
anti-pRb narrow peaks
anti-E2F1
anti-E2F1 narrow peaks
Ref Seq Gene
CCNG2
Fig S15. GGNG2 knockdown leads to growth promotion in CRPC cells.
A: Kaplan Meier survival plot of CCNG2 in prostate cancer patients. B: qRT-PCR analysis of CCNG2 levels following the treatment of PC3-AR cells with C28, SAL or DMSO. Bar graphs are shown as mean ± SEM from six technical replicates (n = 6) of two independent experiments. C: qRT-PCR analysis of CCNG2 levels following the treatment of PC3 cells with C28, Dar, SAL or DMSO from six technical replicates (n = 6) of two independent experiments. D: Analysis of CCNG2 levels in 22Rv1 cells following treatment with C28, SAL or DMSO using qRT-PCR. Bar graphs are shown as mean ± SEM from six technical replicates (n = 6) of two independent experiments. E: Analysis of CCNG2 levels in LNCaP Abl EnzaR spheroids following treatment with C28 or DMSO using qRT-PCR. Bar graphs are shown as mean ± SEM from six technical replicates (n = 6) of two independent experiments. F: Representative images showing SA β-Gal staining in C4-2 cells transfected with siRNA targeting CCNG2 and treated for 48 h with C28 (30 µM), Dar (10 µM), 1 nM R1881 (SAL) or DMSO. G: Crystal violet staining was carried out on the same plates used for SA β-Gal staining, measuring the growth of siCCNG2 transfected cells treated by various AR-ligands. H: Growth assay of transfected C4-2 cells with siCCNG2 following AR-ligand treatments, using crystal violet. Bar graphs are shown as mean ± SEM from four technical replicates (n = 4) of two independent experiments. I: qRT-PCR analysis of CCNG2 levels following the treatment of C4-2 cells with AZ191, DYRK1A-I, in combination with AR-antagonists and -agonist from six technical replicates (n = 6) of two independent experiments. J: Analysis of mRNA levels of DREAM targets following knockdown of CCNG2 in C4-2 cells using qRT-PCR. Bar graphs are shown as mean ± SEM from six technical replicates (n = 6) of two independent experiments. TBP and alpha-Tubulin were used as housekeeping genes for normalization. A two-tailed unpaired Student t-test was performed for statistical analysis. K: ChIP-seq data indicates p130 and E2F1 occupancy at the regulatory regions of CCNG2 in C4-2 cells. IGV software was used for visualization.
Fig. S15
