# Supplementary material for: Induction of cellular senescence by androgen receptor agonist or antagonist is mediated via two novel common DYRK1A-DREAM and cyclin G2 signaling pathways in castration-resistant prostate cancer
Source: J Adv Res. 2025 May 12;80:371–92. doi: 10.1016/j.jare.2025.05.019 (PMC12869228; doi:10.1016/j.jare.2025.05.019)
Supplement: Supplementary Data 3 [file mmc3.docx]

**Immunofluorescence staining**

15000 C4-2 cells were seeded in Chambered Borosilicate Cover glass from Lab-Tek. After 48 h, cells were treated with DMSO, C28 or SAL. Following a 72 h incubation, the cells were washed three times with HBSS buffer (Gibco, 14025-050) and subsequently fixed with 4% formalin for 15 min at room temperature (RT). Post-fixation, the cells underwent three additional washes and were incubated with wheat germ agglutinin (WGA) to stain the plasma membrane. After another wash with HBSS buffer, the cells were permeabilized using 0.2% Triton X100. Following two more washes, the cells were blocked with 5% normal goat serum for 1 h at RT. The cells were then incubated overnight at 4 °C with primary antibodies against AR (1:600, Millipore, #06-680). The next day, the cells were washed and incubated with secondary anti-rabbit IgG Alexa 546 antibody for 1 h at RT in a dark room. Nuclei were stained by incubating the cells with Hoechst (1:10,000, Invitrogen, H3569) for 5 min, followed by a final wash with 1x PBS. The coverslips were then mounted on glass slides using Fluoromount G (BIOZOL, SBA-0100-01). After drying, images were captured using a Zeiss LSM 880 with Airyscan scanning fluorescence microscope equipped with a Plan-Apochromat 63x/1.4 oil DIC M27 objective at super resolution. Quantification was performed using Fiji software ([16](#_ENREF_16)). To quantify fluorescence intensity 10 images per condition were analyzed. Images were first split into individual channels, and the DAPI channel was used to segment nuclei. Tresholding and binary conversion were applied to generate a nuclear mask, which was refined using the watershed option to separate touching nuclei. The region of interest (ROI) was then saved and applied to the protein of interest channel. After adjusting the threshold, fluorescence intensity within the selected nuclei was measured using the analyze and then measure function. Details on statistical analysis are provided in the statistical analysis section.

**Protein extraction and Western blotting**

Cells were lysed using 80 μl of a buffer containing 20 mM Tris-HCl (pH 8.0), 100 mM NaCl, 1mM EDTA, 1% NP-40 and 1% Tergitol, 50mM NaF, 100 μM Na3VO4, and 10mM β-Glycerophosphate , supplemented with a complete protease inhibitor cocktail (Roche). The lysates were centrifuged at 12,000 × g for 10 min at 4 ºC to obtain the cell extracts. Protein concentration was measured using a Nanodrop ND-1000 Spectrophotometer. For analysis, 30µg of protein samples were separated on a 12% SDS polyacrylamide gel. Post-electrophoresis, proteins were transferred onto a PVDF membrane, which was then blocked with skim milk. The membrane was incubated overnight at 4 ºC with specific primary antibodies, washed with1x TBST, and then incubated with corresponding secondary antibodies (Table S1). Anti-beta Actin antibody was used as a loading control.

**RNA extraction and qRT-PCR**

In short: cDNA was synthesized using the High-Capacity cDNA Reverse Transcription Kit (Applied Biosystems; Lithuania; 4368814). Gene expression levels were measured using the SsoAdvanced Universal SYBR Green Supermix (Bio-Rad; USA; 1725271) on a Bio-Rad CFX Duet real-time PCR system. The primers used are listed in supplementary Table S2. The data plots show the mean of the expression levels with error bars representing the SEM.

**Senescence-associated β-galactosidase (SA β-gal) staining**

48 h after seeding cells were treated for 72 h. The cells were washed with 1x PBS and then fixed with 1.5 ml of 1% glutaraldehyde per well for 5 min. After fixation, the cells were washed again with 1x PBS and subsequently stained with 2 ml per well of freshly prepared Senescence associated β-galactosidase (SA β-Gal) staining solution at pH 6.0 for 24 h in a CO_2_ free incubator at 37°C. SA β-Gal activity staining was conducted to assess cellular senescence. For analysis, at least two areas per well were randomly selected, and a minimum of 200 cells per area were counted. The mean percentage of stained cells was calculated from three independent experiments.
